# Discovery of Novel Small-Molecule Inhibitors of PD-1/PD-L1 Interaction via Structural Simplification Strategy

**DOI:** 10.3390/molecules26113347

**Published:** 2021-06-02

**Authors:** Hongbo Zhang, Yu Xia, Chunqiu Yu, Huijie Du, Jinchang Liu, Hui Li, Shihui Huang, Qihua Zhu, Yungen Xu, Yi Zou

**Affiliations:** 1 State Key Laboratory of Natural Medicines, China Pharmaceutical University, Nanjing 210009, China; zhanghongbo397@sina.com (H.Z.); zhuqihua@vip.126.com (Q.Z.); 2 Jiangsu Key Laboratory of Drug Design and Optimization, Department of Medicinal Chemistry, China Pharmaceutical University, Nanjing 210009, China; xiayujacob0213@gmail.com (Y.X.); ycq20151025@163.com (C.Y.); duhuijie1210@163.com (H.D.); 13022509809@163.com (J.L.); 15536369056@163.com (H.L.); hsh17314973301@126.com (S.H.)

**Keywords:** small molecule PD-1/PD-L1 inhibitor, PD-1/PD-L1, structural simplification strategy

## Abstract

Blockade of the programmed cell death 1 (PD-1)/programmed cell death-ligand 1 (PD-L1) interaction is currently the focus in the field of cancer immunotherapy, and so far, several monoclonal antibodies (mAbs) have achieved encouraging outcomes in cancer treatment. Despite this achievement, mAbs-based therapies are struggling with limitations including poor tissue and tumor penetration, long half-life time, poor oral bioavailability, and expensive production costs, which prompted a shift towards the development of the small-molecule inhibitors of PD-1/PD-L1 pathways. Even though many small-molecule inhibitors targeting PD-1/PD-L1 interaction have been reported, their development lags behind the corresponding mAb, partly due to the challenges of developing drug-like small molecules. Herein, we report the discovery of a series of novel inhibitors targeting PD-1/PD-L1 interaction via structural simplification strategy by using BMS-1058 as a starting point. Among them, compound **A9** stands out as the most promising candidate with excellent PD-L1 inhibitory activity (IC_50_ = 0.93 nM, LE = 0.43) and high binding affinity to hPD-L1 (*K*_D_ = 3.64 nM, LE = 0.40). Furthermore, **A9** can significantly promote the production of IFN-γ in a dose-dependent manner by rescuing PD-L1 mediated T-cell inhibition in Hep3B/OS-8/hPD-L1 and CD3-positive T cells co-culture assay. Taken together, these results suggest that **A9** is a promising inhibitor of PD-1/PD-L1 interaction and is worthy for further study.

## 1. Introduction

Programmed cell-death receptor (PD-1) and programmed cell death-ligand 1 (PD-L1) inhibitors have achieved significant success in the oncology community both preclinically and clinically [1,2,3]. PD-L1, typically expressed on the surface of tumor cells, is bound to PD-1 on tumor-infiltrating lymphocytes, thereby inhibiting T-cell functions and consecutively inducing tumor immune escape [4,5,6,7]. Consequently, blocking PD-1/PD-L1 interactions has become a prospective approach in cancer treatment, which can restore immune system and elicit a favorable tumor-specific T cells response [8]. In the past few years, several monoclonal antibodies (mAbs) targeting PD-1 or PD-L1 have been approved in clinical use by the U.S. Food and Drug Administration (FDA) [9], exhibiting significant benefits with durable clinical responses and acceptable treatment-related toxicities in several types of solid tumors [10,11,12]. Although these mAbs have transformed cancer immunotherapy forever, they still exhibit several disadvantages such as very long half-life, immune-related adverse effects (irAEs), low permeability, immunogenicity, complex production process, and exorbitant treatment costs from another perspective [13]. Among the reported immunological adverse events, the overall incidences of all-grade adverse events were 66.0% and of grade 3 or higher adverse events were 14.0%. The most common side effects are endocrinological disturbances, dermatologic manifestations, and gastrointestinal disturbances. Apart from this, the most common grade 3 or higher adverse events were fatigue, anemia, and aspartate aminotransferase increase [14,15]. Compared to mAbs, small-molecule drugs have attracted tremendous attentions in immuno-oncology in recent years, due to their better pharmacokinetic properties and diffusion rates and convenient manufacture [16].

**Figure 1:**
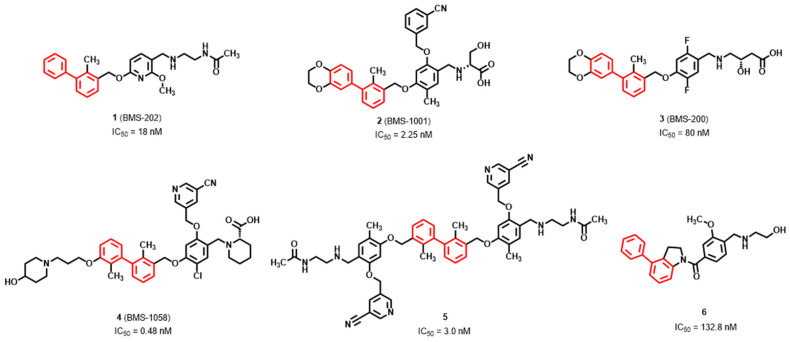
Structures of the reported compounds with binding PD-1/PD-L1 interaction. Biphenyl moiety is colored by red. IC_50_ values are established by the HTRF binding assay.

Recently, several small molecules derived from 2-methyl-3-biphenyl methanol scaffold were disclosed as effective inhibitors of PD-1/PD-L1 interaction (Figure 1) [17,18,19,20,21]. Most of the inhibitors show moderate to excellent activities of blocking PD-1/PD-L1 protein–protein interaction (PPI) with IC_50_ values between picomolar to nanomolar level through europium (Eu)−allophycocyanin (APC) homogeneous time-resolved fluorescence (HTRF) binding assay. The first co-crystal complex structure of small-molecule BMS-202 and hPD-L1 reveals that this compound induces and stabilize the formation of PD-L1 homodimers, leading to the blockade of PD-1/PD-L1 interaction [20,22,23].

Since small molecules for immuno-oncology therapeutics have not been approved yet, future research should continue to focus on the discovery of novel small molecules activating the immune system with orally bioavailable compounds to combat cancer. Although some compounds bearing biphenyl moiety and their bivalent derivatives can inhibit the interaction of PD-1 with PD-L1 with IC_50_ values of ≤10 nM [22,23,24], most of the inhibitors have inherent disadvantages such as high molecular weight (MW), molecular polarity, and degree of dissociation, which might be responsible for the poor pharmacokinetic (PK) profile in preclinical and clinical studies [20,25,26]. Therefore, it still needs further studies to design small-molecule inhibitors of PD-1/PD-L1 interaction with better physicochemical properties. In the present study, we designed and synthesized a novel series of 2-(2-methyl-[1,1′-biphenyl]-3-yl) pyridine derivatives targeting PD-1/PD-L1 interaction via structural simplification strategy. During our design process, several structural indices, including MW, ALogP, and polar surface area (PSA), were considered to monitor their potential drug ability. The ligand efficiency (LE), a measure for the activity normalized by the number of non-H atoms, was also used as a valuable criterion for evaluating the quality of molecular design. Note that utilization of this metric during the overall drug design process to control the balance of molecular size and potency will significantly improve success rates [27]. Fortunately, our research leads to the identification of compound **A9** that shows wonderful inhibitory activity at molecular level with a high LE value (HTRF IC_50_ = 0.93 nM, LE = 0.43; surface plasmon resonance (SPR) *K*_D_ = 3.64 nM, LE = 0.40). In addition, **A9** potently induces the release of IFN-γ in a Hep3B/OS-8/hPD-L1 and primary T cell co-culture system, deserving further investigation on its role in restoring immune surveillance.

## 2. Results and Discussion

### 2.1. Design and Screening

**Figure 2:**
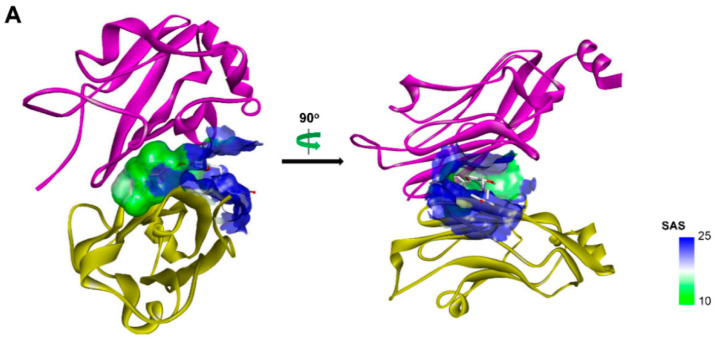

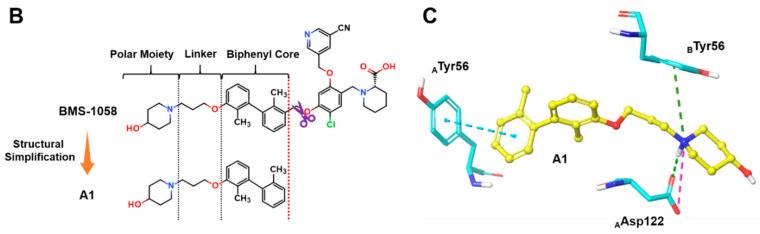
(**A**) Crystal structure of PD-L1 (chain A, yellow; chain B, purple)/BMS-202(white) complex (PDB code: 5J89). Receptor surfaces, covering atoms that are close to the current ligand, are colored by solvent accessibility surface (SAS). The default color spectrum used is green–white–blue, and small values (green) correspond to buried residues, whereas large values (blue) correspond to exposed residues. This picture was drawn by Discovery Studio Visualizer [29]. (**B**) Strctural simplification strategy of this work. (**C**) Putative binding modes of **A1** in the binding pocket of PDL1 dimer structure. Dashed lines represent the inter-interaction between **A1** and protein, specifically, green ones indicate hydrogen bond, dark green ones indicate cation–π interaction, purple ones indicate ionic interaction, and sky-blue ones indicate π–π interaction.

In our pursuit of novel small molecule inhibitors targeting the PD-1/PD-L1 pathway, we focused our effort on designing molecules with better drug-likeness property. BMS-1058 (IC_50_ = 0.48 nM) and BMS-202 (IC_50_ = 18 nM), which inhibited the PD-1/PD-L1 interaction in HTRF binding assay, were chosen as the template compounds for structural optimization. Structural analysis of the X-ray crystal complex of BMS-202 and hPD-L1 showed that it comprises three essential pharmacophores: (1) The biphenyl core, the minimal fragment essential for the hPD-L1 binding; (2) the polar moiety, the fragment interacting with the polar residues in solvent-oriented region; (3) the linker connecting these two regions (Figure 2A). We noticed that BMS-1058, with excellent IC_50_ values of 0.48 nM but possessing high MW and PSA value, has two polar parts, located at opposite ends of this molecule. Thus, it provided us a clue to design compounds by removing one side of the polar parts of BMS-1058, leading to the discovery of compound **A1** (Figure 2B). It has moderate IC_50_ value of 113.6 nM but shows higher ligand-binding efficiency (LE = 0.38) than that of BMS-1058 (LE = 0.24) and BMS-202 (LE = 0.33) (Table 1), which gave us a valuable starting point for further optimization. In order to rationally design more potent inhibitors without reducing LE value, the in-silico docking prediction was conducted using Glide module in Schrödinger [28]. As shown in Figure 2C, the biphenyl core of **A1** anchors at the bottom of the pocket, and two methyl groups form hydrophobic interactions with neighboring hydrophobic residues. In addition, one of the phenyl rings forms a T-stacking interaction with the sidechain of _A_Tyr56. The protonated piperidin-4-ol moiety faces solvent-exposed environment and interacts with _A_Asp122 and _B_Tyr56 by ionic and cation-π interactions, respectively. On the basis of this analysis, optimization of **A1** would be employed by the following considerations: Substitutes on the distal phenyl ring, the linker, and the polar group in the tail.

**Figure 3:**
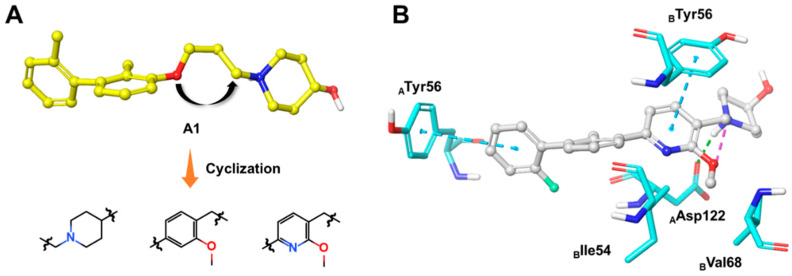
(**A**) The optimization strategy of the linker part. (**B**) Putative binding modes of **A4** in the binding pocket of PD-L1 dimer structure (PDB code: 5J89). Dashed lines represent the inter-interaction between **A4** and protein, specifically, green ones indicate hydrogen bond, purple ones indicate ionic interaction, and sky-blue ones indicate π–π interaction.

On closer inspection, however, it becomes clear that the binding conformation of aliphatic chain linker may be unfavorable for ligand binding owing to the adverse conformational entropy effects [30]. Thus, as the initial step, compounds with several cyclic linkers were prepared, where the methyl group of the distal phenyl ring were also replaced by the fluoro group in order to reduce its electron density (Table 1). Although the replacement using piperidine linker leads to a significant drop in potency, compounds with methoxy substituted benzene and pyridine rings do exhibit a significant improvement in activity as well as LE values (**A3** IC_50_: 18.4 nM, LE: 0.35; **A4** IC_50_: 21.9 nM, LE: 0.36). Docking studies suggest that **A4** establishes an additional–π interaction with _B_Tyr56 compared with **A1**, and the methoxy group fills the hydrophobic cavity defined by _B_Ile54 and _B_Val68 (Figure 3), which may contribute to the ligand binding. Although **A3** and **A4** show a certainly decrease in activity compared with BMS-1058, they have higher LE values and proper drug-likeness properties such as ALogP and PSA, which encouraged us to make further modifications. Given the lower electron density of the central pyridine ring and solubility property, **A4** was chosen to explore new molecules blocking PD-1/PD-L1 axis.

**Figure 4:**
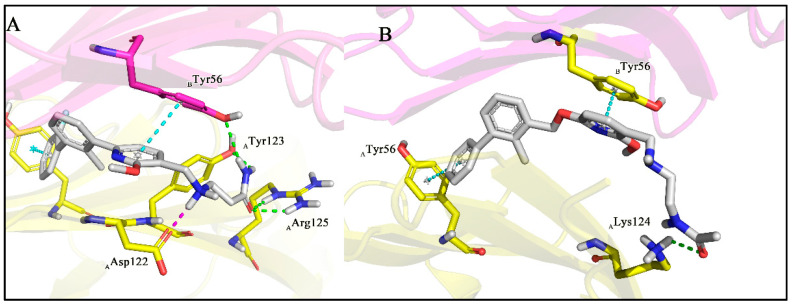
(**A**) Putative binding modes of **A9** in the binding pocket of PD-L1 dimer structure (PDB code: 5J89); (**B**) binding modes of BMS-202 in the binding pocket of PD-L1 dimer structure (PDB code: 5J89). Dashed lines represent the inter-interaction between **A9** or BMS-202 and protein, specifically, green ones indicate hydrogen bond, purple ones indicate ionic interaction, and sky-blue ones indicate π-π interaction.

Continuing to search for ways to improve the affinity, we turned our attention to the modification of the polar moiety in the tail, and a series of compounds were designed and synthesized. The results of biological activities are summarized in Table 2. Compounds **A5**, **A7,** and **A8**, decorated with 4-aminocyclohexan-1-ol, piperidine-4-carboxamide, and piperidine-4-carboxylic acid, respectively, are somewhat better tolerated than compound **A6** with piperidine-3-carboxamide moiety. To our delight, the introduction of liner β-alaninamide to the polar tail (**A9**, IC_50_ = 0.93 nM) leads to an increase in the activity of more than 23-fold as compared with **A4**. Our CADD studies demonstrate that secondary aliphatic amine in the β-alaninamide, which is protonated at pH 7.4, forms a strong attractive charge interaction with _A_Asp122. This kind of strong interaction was observed in all the top-ranked docking poses. The amine group of an amide simultaneously forms two hydrogen bonds with _B_Tyr56 and _A_Tyr123, and its carbonyl oxygen also interacts with _A_Arg125 by a hydrogen bonding interaction, possibly contributing to the high affinity (Figure 4A). Compared with BMS-202 (Figure 4B), **A9** had similar spatial tendencies within the binding interface of dimer-PD-L1. However, more residues have participated in the formation of strong polar interactions between **A9** and PD-L1. Based on the biological data results of **A9**, β-alaninamide group has been proved to be the most suitable R_3_ substituent.

Decorating the distal phenyl ring with various substituents provided further structure-activity relationships (SARs). The replacement of 2-fluoro by the bulkier 2-methyl (**A10**, IC_50_ = 10.54 nM) or 2-trifluoromethyl group (**A12**, IC_50_ = 51.5 nM) leads to a decrease in the activity of more than 10-fold as compared with **A9**. The chloro substituted compound (**A11**, IC_50_ = 6.18 nM) exhibited slightly higher inhibitory activity compared to **A10**. As expected, the unsubstituted phenyl ring **A13** (**A13**, IC_50_ = 1.94 nM) yields an approximately 3-fold increase in potency over compound **A11**, which, however, does not reach the potency of 2-fluoro derivative **A9**. Moving the 2-fluoro to the 3-or 4-position shows comparable activity with **A9**, which indicates that the position of mono substituted fluoro has no significant effect on activity. This may be attributed by the more favorable π–π interaction between the electron-deficient fluorobenzene of these compounds and the residue _A_Tyr56. However, the activity is dramatically reduced as for disubstituted derivatives **A16**–**A18** (**A16**, IC_50_ = 60.0 nM; **A17**, IC_50_ = 866.0 nM; **A18**, IC_50_ = 790.9 nM). This is probably due to the unfavorable steric interactions between atom pairs in close contacts. Although the conformation of _A_Tyr56 can be moved to accommodate to the size of substituent group on the distal phenyl ring, the binding affinity cannot be compensated for the energy loss by conformational change of _A_Tyr56. In general, we have obtained the target compound **A9**, which has potential activity and moderate water solubility (126.37 μg/mL at pH 7.4 vs. BMS-202 89.99 μg/mL at pH 7.4), and we would conduct subsequent biological activity evaluations of **A9**.

### 2.2. Biophysical Characterization of Compounds Binding to Recombinant Human PD-L1 (hPD-L1)

**Figure 5:**
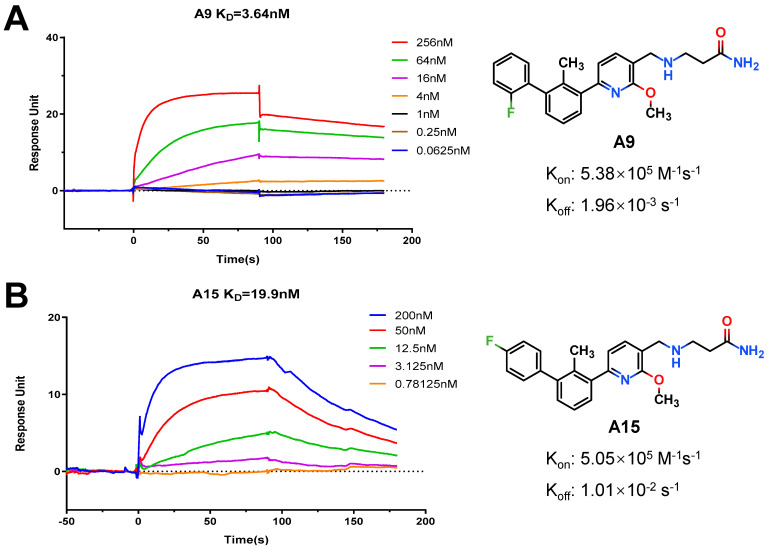
Association and dissociation kinetic curves of compounds **A9** (**A**) and **A15** (**B**) at different concentrations with hPD-L1.

To provide definitive evidence of direct binding to PD-L1 with small-molecule inhibitors, an SPR-based binding assay was performed using Biacore T200 instrument, which facilitates measurement of the kinetic parameters of ligand–protein complex formation. In this determination, hPD-L1/Fc was loaded on a Series S Sensor Chip Protein A. **A9** and **A15**, with similar bioactivities in HTRF assay, were gradually subjected to this assay, and their kinetic parameters and the binding affinities were determined with the aid of Biacore evaluation software. Analyses of the resulting data reveals that both **A9** and **A15** can efficiently interact with the immobilized hPD-L1 protein, as demonstrated by the concentration-dependent responses for association and dissociation, respectively (Figure 5, Table S1). The equilibrium dissociation constant (*K*_D_) could be calculated and shows that compound **A9** binds hPD-L1 with an excellent *K*_D_ value of 3.64 nM, 5-fold greater than that of **A15** (*K*_D_ = 19.9 nM). Noteworthily, their kinetic measurements demonstrate that **A9** and **A15** have nearly the same association rate but show conspicuous differences in dissociation rates that may lead to selection of **A9** with a slower off-rate as a more effective blocker of PD-1/PD-L1 interaction.

### 2.3. Non-Specific Toxicity of (2-Methyl-[1,1′-Biphenyl]-3-yl)Pyridine Derivatives

Considering that the toxicity of the compound might strongly interfere with the assay if the tested compounds could block PD-1/PD-L1 interaction to activate T cells for killing tumors, we examined their cell toxicities of MDA-MB-231 and Jurkat T cells in MTT assay. As shown in Table 3, compounds **A9**, **A11**, **A13**, **A14,** and **A15** do not directly kill tumor cells and normal T cells. Furthermore, Jurkat T cells were used to simulate primary T cells for investigating the immune toxicity of the treated compounds with the regular concentrations within 48 h. This assay also shows that **A9**, **A11,** and **A15** exhibit lower cell toxicity, in which their EC_50_ values are much higher than their respective IC_50_ values at the molecular level.

### 2.4. IFN-γ Production Effect of ***A9*** in T-Cell Function Assay

Considering the excellent potency in biochemical assays and acceptable drug-likeness properties, we concluded that **A9** is optimal, and would be chosen for further study at T cell function assay. It is well-known that the expression of interferon-γ (IFN-γ) in the tumor microenvironment is increased by a productive T-cell response against tumor-associated antigens [6,31,32,33]. However, it will be prevented once PD-L1 is overexpressed on tumor surface through PD-1/PD-L1 interaction. In the presence of PD-1/PD-L1 blockers, the interaction between PD-1 and PD-L1 will be destroyed, and the expression of IFN-γ would be restored by activated T cells.

**Figure 6:**
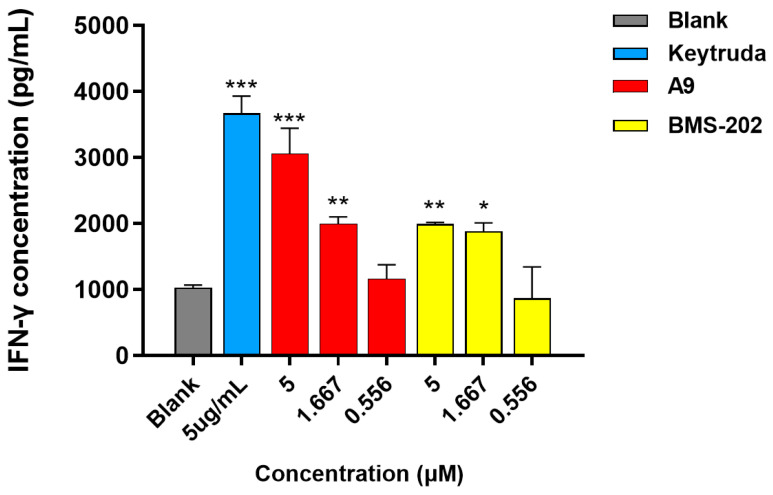
Effects of **A9** on IFN-γ expression in a Hep3B/OS-8/hPD-L1 and CD3^+^ T-cell co-culture assay. **A9** and BMS-202, and three independent experiments for blank and Keytruda. Data are shown as mean ± SD (**A9** and BMS-202: *n* = 2; Blank and Keytruda: *n* = 3), * *p* < 0.05, ** *p* < 0.01, *** *p* < 0.001 vs. blank group.

To evaluate if compound **A9** could restore the T cells-mediated immunity responses, which was previously repressed by the PD-1/PD-L1 pathway, Hep3B cells, engineered to stably express OS-8 and hPD-L1, were co-cultured with primary CD3^+^ T Cells in the presence of many PD-1/PD-L1 blockers (Figure 6). A PD-1 monoclonal antibody Keytruda and small molecule BMS-202 were synchronously used as positive controls. Obviously, **A9** can promote the dose-dependent release of IFN-γ in this co-culture system. Impressively, the promoting effect on IFN-γ production of **A9** at 5 μM was comparable to that of Keytruda at 5 μg/mL and is significantly higher than that of BMS-202. This result implies that **A9** can restore T cells-mediated immune responses by blocking the PD-1/PD-L1 interaction in a tumor microenvironment.

### 2.5. Chemistry

**Scheme 1:**
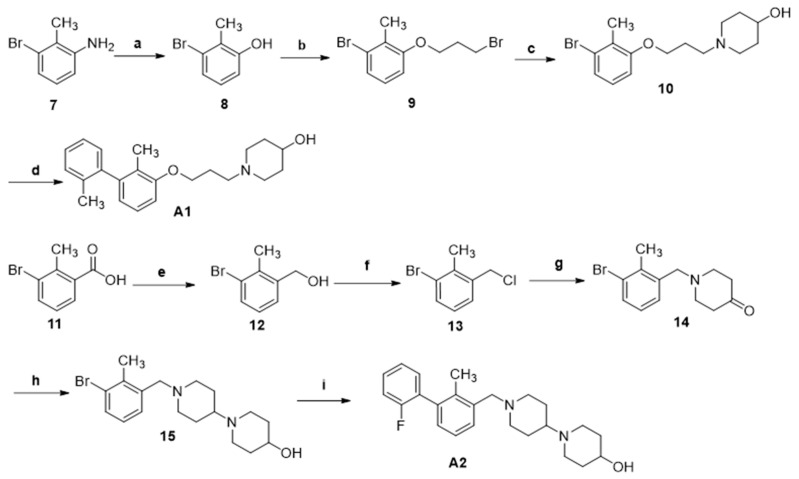
Regents and conditions: (a) 1M H_2_SO_4_, 30% NaNO_2_, Toluene, 0–100 °C, 1 h, 89.6%; (b) Br(CH_2_)_3_Br, K_2_CO_3_, acetone, 65 °C, overnight, 81.7%; (c) piperidin-4-ol, acetonitrile, DIPEA, 80 °C, overnight, 80.2%; (d) 2-methylphenylboronic acid, Pd(PPh_3_)_4_, K_2_CO_3_, 1,4-dioxane/H_2_O (V:V = 10/1), 80 °C, 8 h, 82%; (e) BH_3_-THF, THF, 0 °C then r.t, 12 h, 97.0%; (f) SOCl_2_, reflux, 2 h, 98.0%; (g) 4-piperidinone, acetonitrile, DIPEA, 80 °C, overnight, 72.1%; (h) piperidin-4-ol, HOAc, CH_2_Cl_2_/MeOH, r.t, 2 h; NaBH(OAc)_3_, r.t., 8 h, 58.3%; (i) 2-fluorophenylboronic acid, Pd(PPh_3_)_4_, K_2_CO_3_, 1,4-dioxane/H_2_O(V:V = 10:1), 80 °C, 8 h, 89.3%.

The synthesis of compounds **A1** and **A2** is shown in Scheme 1. The key intermediate **8** was prepared through the Sandmeyer reaction of the starting material **7** and reacted with Br(CH_2_)_3_Br to obtain intermediate **9** by Williamson ether synthesis. Then, the intermediate **10** was obtained by *N*-alkylation reaction with piperidin-4-ol, which was converted to the desired compound **A1** through Suzuki coupling reaction with 2-methylphenyl boronic acid.

Intermediate **12**, which was prepared from **11** by BH_3_-THF mediated reductive reaction, was reacted with SOCl_2_ to afford **13**, followed by the reaction with piperidone to provide **14**. Then, **14** was converted into **15** by NaBH(OAc)_3_-mediated reductive amination reaction. Finally, the Suzuki-coupling reaction was conducted between **15** and 2-fluorophenylboronic acid to give the target compound **A2**.

**Scheme 2:**
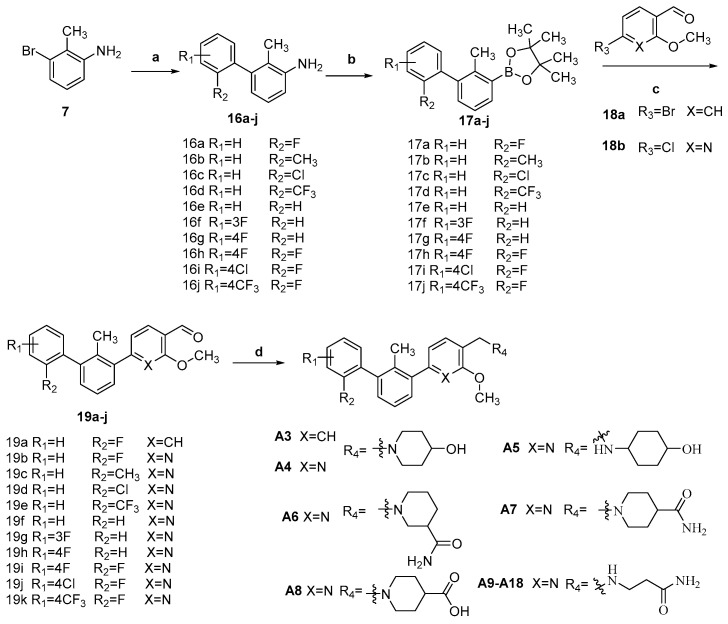
Regents and conditions: (**a**) Substituted phenylboronic acid, Pd(PPh_3_)_4_, K_2_CO_3_, 1,4-dioxane/H_2_O (V:V = 10/1), 80 °C, 8 h, 80.2–99.2%; (**b**) 3M HCl, 30% NaNO_2_, B_2_pin_2_, MeOH, water, 0 °C ~rt, 40.1–68.8%; (**c**) **18a** or **18b**, Pd(PPh_3_)_4_, K_2_CO_3_, 1,4-dioxane/H_2_O (V:V = 10/1), 80–90 °C, 12 h, 20.8–95.3%; (**d**) alkyl amine, HOAc, CH_2_Cl_2_/MeOH (V:V = 3/1), r.t., 4–12 h; NaBH(OAc)_3_, r.t., 8 h, 19.9–64.4%.

The target compounds **A3**–**19** were prepared according to the methods summarized in Scheme 2. Commercially, material **7** was coupled with various substituted phenylboronic acid to give **16a**–**j** by Suzuki coupling reaction, which was then converted to **17a**–**j** by the Sandmayer reaction. The resulting intermediates **17a**–**j** were directly used to synthesize the intermediates **19a**–**k** with **18** via Suzuki coupling reaction, which were finally reacted with appropriate amines to yield target compounds **A3**–**19** through the NaBH(OAc)_3_ mediated reductive amination reaction.

## 3. Materials and Methods

### 3.1. Synthesis

Starting materials and solvents were purchased from commercial suppliers and used without any additional purification. ^1^H NMR and ^13^C NMR spectra were obtained on a Bruker AV-300 nuclear instrument or Bruker AVANCE NEO 400 MHz in CDCl_3_ or DMSO-*d_6_* using TMS as internal standard, operating at 300 MHz or 400 MHz and 75 MHz, respectively. Chemical shifts (δ) are expressed in ppm and coupling constants *J* are given in Hz. Analytical thin layer chromatography (TLC) was purchased from Yantai Chemical Industry Research Institute (Cat. no. HSGF254, Yantai, China). All the reactions were monitored by thin layer chromatography in UV absorbance (254 nm). Flash chromatography was performed using silica gel (200–300 or 300–400 mesh). Melting points were measured with an RY-I melting point apparatus. The HPLC analysis was performed on a Shimadzu LC-20AT machine with a BDS Hypersil C18 column, and the column temperature was at 31 °C. Mobile phase B (100% Acetonitrile) and mobile phase A (NaH_2_PO_4_ and H_3_PO_4_ buffer solution, pH = 7.5) were used in a gradient elution program (0 min: 25% (B), 5 min: 25% (B), 12 min: 75% (B), 20 min: 75% (B), 23 min: 25% (B), 25 min: 25% (B)) with a flow rate of 1.0 mL/min at 254 nM.

#### 3.1.1. 3-Bromo-2-Methylphenol (**8**)

A mixture of 3-bromo-2-methylaniline and H_2_SO_4_ (1M, 65 mL) was stirred for 30 min at room temperature, then 30% NaNO_2_ (2.25 g, 32.60 mmol) was added to the mixture dropwise at 0–5 °C. After 30 min, toluene (50 mL) was then added to the reaction mixture and allowed to stir at 100 °C for approximately 1 h. The mixture was extracted with ethyl acetate (50 mL × 3) and washed with brine (50 mL × 2). The combined organic layer was dried over anhydrous Na_2_SO_4_, filtered, and concentrated in vacuum. The residue was purified by silica gel chromatography (Petroleum ether/ethyl acetate = 100/1–60/1) to afford compound **8** (white solid, 4.50 g, yield: 89.6%). m.p. 95.0–98.0 °C. ^1^H NMR (300 MHz, Chloroform-*d*) δ 7.19 (dd, *J* = 8.0, 1.2 Hz, 1H, ArH), 6.97 (t, *J* = 8.0 Hz, 1H, ArH), 6.76 (d, *J* = 7.9 Hz, 1H, ArH), 4.82 (s, 1H, OH), 2.38 (s, 3H, CH_3_).

#### 3.1.2. 1-Bromo-3-(3-Bromopropoxy)-2-Methylbenzene (**9**)

A mixture of **8** (5.00 g, 26.90 mmol), K_2_CO_3_ (9.28 g, 67.25 mmol), and 1,3-dibromopropane (10.75 g, 53.80 mmol) in acetone (30 mL) was refluxed for 8 h. The mixture was diluted with water (100 mL), extracted with ethyl acetate (50 mL × 3), and washed with brine (50 mL × 3). The combined organic layer was dried over anhydrous Na_2_SO_4_, filtered, and concentrated in vacuum. The residue was purified by silica gel chromatography (Petroleum ether = 100%) to afford compound **9** (colorless oil, 6.72 g, yield: 81.7%).^1^H NMR (300 MHz, Chloroform-*d*) δ 7.16 (dd, *J* = 8.1, 1.2 Hz, 1H, ArH), 7.00 (td, *J* = 8.1, 0.6 Hz, 1H, ArH), 6.79 (dd, *J* = 8.2, 1.2 Hz, 1H, ArH), 4.10 (t, *J* = 5.7 Hz, 2H, OCH_2_), 3.62 (t, *J* = 6.4 Hz, 2H, BrCH_2_), 2.39–2.32 (m, 2H, CH_2_CH_2_CH_2_), 2.31 (s, 3H, CH_3_).

#### 3.1.3. 1-(3-(3-Bromo-2-Methylphenoxy)Propyl)Piperidin-4-ol (**10**)

A mixture of **9** (1.00 g, 3.27 mmol), DIPEA (1.10 mL, 6.54 mmol), and 4-hydroxypiperidine (0.97 g, 3.27 mmol) in MeCN (10 mL) was refluxed for 12 h. The formed precipitates were filtrated and washed with petroleum ether and less water to obtain compound **10** (yellow solid, 0.86 g, yield: 80.8%). m.p.138.0–140.0 °C. ^1^H NMR (300 MHz, DMSO-*d*_6_) δ 7.20 (dd, *J* = 8.0, 1.3 Hz, 1H, ArH), 7.13 (t, *J* = 8.1 Hz, 1H, ArH), 6.99 (d, *J* = 7.9 Hz, 1H, ArH), 5.02–4.96 (m, 1H, OH), 4.09 (t, *J* = 5.9 Hz, 2H, OCH_2_), 4.01–3.92 (m, 1H, CHOH), 3.77–3.40 (m, 2H, CH_2_N), 3.20–2.86 (m, 4H, NCH_2_), 2.28 (s, 3H, CH_3_), 2.23–2.08 (m, 2H, CH_2_CH_2_CH_2_), 2.08–1.69 (m, 4H, CH_2_CHOH).

#### 3.1.4. (3-Bromo-2-Methylphenyl)Methanol (**12**)

BH_3_-THF (1 M, 35 mL) was dropwise added to a solution of 3-bromo-2-methylbenzoic acid (5.00 g, 23.20 mmol) in dry THF (25 mL) at 0 °C under N_2_. The mixture was stirred at an ambient temperature for 12 h. The reaction quenched with 1 M HCl (30 mL), and then extracted with ethyl acetate (20 mL × 3), followed by brine (20 mL × 3). The combined organic layer was dried over anhydrous Na_2_SO_4_, filtered, and concentrated in vacuum to give **12** without any further purification (white solid, 4.5 g, yield, 97.0%). m.p. 103.0–104.0 °C. ^1^H NMR (300 MHz, DMSO-*d*_6_) δ 7.49 (d, *J* = 7.9 Hz, 1H, ArH), 7.39 (d, *J* = 7.8 Hz, 1H, ArH), 7.12 (t, *J* = 8.1 Hz, 1H, ArH), 5.27 (br, 1H, OH), 4.52 (s, 2H, CH_2_), 2.30 (s, 3H, ArCH_3_).

#### 3.1.5. 1-Bromo-3-(Chloromethyl)-2-Methylbenzene (**13**)

A solution of **12** (4.50 g, 22.50 mmol) in SOCl_2_ (10 mL) was stirred at 80 °C for 2 h. The solvent was removed in vacuum to obtain compound **13** (colorless oil, 4.80 g, yield, 98.0%). ^1^H NMR (300 MHz, Chloroform-*d*) δ 7.47 (d, *J* = 7.0 Hz, 1H, ArH), 7.25 (d, *J* = 6.8 Hz, 1H, ArH), 7.04 (t, *J* = 8.1 Hz, 1H, ArH), 3.90 (s, 2H, CH_2_), 2.43 (s, 3H, CH_3_).

#### 3.1.6. 1-(3-Bromo-2-Methylbenzyl)Piperidin-4-One (**14**)

This compound was prepared following the synthetic procedure similar to that of **10**. Compound **14** (colorless oil 0.93 g, yield, 72.1%). ^1^H NMR (300 MHz, Chloroform-*d*) δ 7.52 (dd, *J* = 8.0, 1.6 Hz, 1H, ArH), 7.23 (d, *J* = 6.4 Hz, 1H, ArH), 7.03 (t, *J* = 7.8 Hz, 1H, ArH) 3.61 (s, 2H, CH_2_N), 2.77 (t, *J* = 6.1 Hz, 4H, 2NCH_2_), 2.51 (s, 3H, CH_3_), 2.46 (t, *J* = 6.2 Hz, 4H, 2CH_2_CO).

#### 3.1.7. 1′-(3-Bromo-2-Methylbenzyl)-[1,4′-Bipiperidin]-4-ol (**15**)

A mixture of **14** (400 mg, 1.42 mmol) and 4-hydroxypiperidine (172 mg, 1.71 mmol) in DCM (10 mL) was stirred for 2 h at room temperature. NaBH(OAc)_3_ (2.30 mmol) was added to the mixture slowly at 0 °C and stirred for another 8 h. The reaction was adjusted to pH 7–8 with saturated NaHCO_3_ aqueous solution. The mixture was extracted with DCM (10 mL × 3) and washed with brine (10 mL × 3). The combined organic layer was dried over anhydrous Na_2_SO_4_, filtered, and concentrated in vacuum. The residue was purified by silica gel chromatography (dichloromethane/methanol = 100/1–30/1) to afford compound **15** (yellow solid, yield, 58.3%). m.p. 102.0–105.0 °C. ^1^H NMR (300 MHz, Chloroform-*d*) δ 7.51 (d, *J* =7.9Hz, 1H, ArH), 7.23 (d, *J* = 7.4 Hz, 1H, ArH), 7.03 (t, *J* = 7.7 Hz, 1H, ArH), 4.34–4.27 (m, 1H, CHOH), 3.79–3.66 (m, 1H, CHN), 3.47 (s, 2H, ArCH_2_), 3.02–2.80 (m, 4H, NCH_2_), 2.46 (s, 3H, ArCH_3_), 2.44–2.28 (m, 4H, NCH_2_), 2.05–1.92 (m, 4H, 2CH_2_), 1.55–1.70 (m, 4H, 2CH_2_).

#### 3.1.8. The Synthesis of Compounds **16a**–**j**

Compounds **16a**–**j** were synthesized by the following procedure. To a solution of 3-bromo-2-methylaniline (1.0 mmol), phenylboronic acid (1.2 mmol), K_2_CO_3_ (2.8 mmol) in 1,4-dioxane/water (1.0 mL/0.1 mL), Pd(PPh_3_)_4_ (0.05 mmol) was added. The mixture was stirred at 80 °C for 8 h under N_2_ atmosphere. The undissolved solid was filtered off, and the filtrate was concentrated under vacuum. The residue was dissolved in ethyl acetate, and washed with water, followed by brine. The organic layer was dried over anhydrous Na_2_SO_4_, filtered, and concentrated. The residue was purified by silica gel chromatography (petroleum ether/ethyl acetate = 100/1–80/1) to afford compounds **16a**-**j**.

2′-Fluoro-2-methyl-[1,1′-biphenyl]-3-amine (**16a**)

Brown solid. Yield, 95.6%. m.p. 61.0–62.0 °C. ^1^H NMR (300 MHz, DMSO-*d*_6_) δ 7.48–7.35 (m, 1H, ArH), 7.34 – 7.22 (m, 3H, ArH), 6.98 (t, *J* = 7.7 Hz, 1H, ArH), 6.70 (dd, *J* = 8.0, 1.4 Hz, 1H, ArH), 6.42 (dd, *J* = 7.5, 1.3 Hz, 1H, ArH), 5.00 (s, 2H, NH_2_), 1.85 (d, *J* = 1.8 Hz, 3H, ArCH_3_).

2,2′-Dimethyl-[1,1′-biphenyl]-3-amine (**16b**)

Yellow oil. Yield, 90%. m.p. 52.0–55.0 °C. ^1^H NMR (400 MHz, Chloroform-*d*) δ 7.34 – 7.29 (m, 1H, ArH), 7.28–7.22 (m, 2H, ArH), 7.15 (d, *J* = 7.0 Hz, 1H, ArH), 7.10 (t, *J* = 7.7 Hz, 1H, ArH), 6.75 (dd, *J* = 7.9, 1.4 Hz, 1H, ArH), 6.63 (dd, *J* = 7.5, 1.4 Hz, 1H, ArH), 3.75 (s, 2H, ArNH_2_), 2.11 (s, 3H, ArCH_3_), 1.91 (s, 3H, ArCH_3_).

2′-Chloro-2-methyl-[1,1′-biphenyl]-3-amine (**16c**)

Yellow oil. Yield, 93.1%. m.p. 71.5–74.5 °C. ^1^H NMR (300 MHz, DMSO-*d*_6_) δ 7.58–7.46 (m, 1H, ArH), 7.43–7.30 (m, 2H, ArH), 7.30–7.18 (m, 1H, ArH), 6.94 (t, *J* = 7.6 Hz, 1H, ArH), 6.67 (dd, *J* = 8.0, 1.4 Hz, 1H, ArH), 6.32 (dd, *J* = 7.5, 1.4 Hz, 1H, ArH), 4.96 (s, 2H, NH_2_), 1.76 (s, 3H, ArCH_3_).

2′-Chloro-2-methyl-[1,1′-biphenyl]-3-amine (**16d**)

Yellow solid. Yield, 82.1%. m.p. 87.5–90.0 °C. ^1^H NMR (300 MHz, DMSO-*d*_6_) δ 7.79 (dd, *J* = 7.9, 1.6 Hz, 1H, ArH), 7.67 (td, *J* = 7.6, 1.7 Hz, 1H, ArH), 7.56 (t, *J* = 7.6, 1H, ArH), 7.25 (d, *J* = 7.5 Hz, 1H, ArH), 6.90 (t, *J* = 7.7 Hz, 1H, ArH), 6.67 (dd, *J* = 8.0, 1.4 Hz, 1H, ArH), 6.31 (d, *J* = 7.3 Hz, 1H, ArH), 4.98 (s, 2H, NH_2_), 1.68 (s, 3H, ArCH_3_).

2-Methyl-[1,1′-biphenyl]-3-amine (**16e**)

White solid. Yield, 89.6%. ^1^H NMR (400 MHz, Chloroform-*d*) δ 7.46–7.40 (m, 2H, ArH), 7.40–7.32 (m, 3H, ArH), 7.11 (t, *J* = 7.8 Hz, 1H, ArH), 6.75 (d, *J* = 7.8 Hz, 2H, ArH), 3.82 (s, 2H, NH_2_), 2.10 (s, 3H, ArCH_3_).

3′-Fluoro-2-methyl-[1,1′-biphenyl]-3-amine (**16f**)

Yellow oil. Yield, 88.0%.^1^H NMR (400 MHz, DMSO-*d*_6_) δ 7.44 (td, *J* = 8.1, 6.1 Hz, 1H, ArH), 7.25–7.15 (m, 1H, ArH), 7.12–7.04 (m, 2H, ArH), 6.94 (t, *J* = 7.8 Hz, 1H, ArH), 6.67 (dd, *J* = 8.0, 1.5 Hz, 1H, ArH), 6.42 (dd, *J* = 7.5, 1.4 Hz, 1H, ArH), 5.05 (s, 2H, NH_2_), 1.93 (d, *J* = 1.9 Hz, 3H, CH_3_).

4′-Fluoro-2-methyl-[1,1′-biphenyl]-3-amine (**16g**)

White solid. Yield, 88.0%. m.p. 76.5–78.0 °C. 1H NMR (300 MHz, DMSO-*d*_6_) δ 7.32–7.23 (m, 3H, ArH), 7.20 (d, *J* = 8.8 Hz, 1H, ArH), 6.93 (t, *J* = 7.7 Hz, 1H, ArH), 6.66 (d, *J* = 7.9 Hz, 1H, ArH), 6.40 (d, *J* = 7.5 Hz, 1H, ArH), 4.94 (s, 2H, NH2), 1.92 (s, 3H, ArCH3).

2′,4′-Difluoro-2-methyl-[1,1′-biphenyl]-3-amine (**16h**)

Yellow solid. Yield, 88.9%. m.p. 81.5–83.0 °C. ^1^H NMR (300 MHz, DMSO-*d*_6_) δ 7.38–7.27 (m, 2H, ArH), 7.16 (td, *J* = 8.5, 2.2 Hz, 1H, ArH), 6.97 (t, *J* = 7.8 Hz, 1H, ArH), 6.70 (dd, *J* = 8.1, 1.3 Hz, 1H, ArH), 6.40 (d, *J* = 7.4 Hz, 1H, ArH), 5.01 (s, 2H, NH_2_), 1.84 (d, *J* = 1.8 Hz, 3H, ArCH_3_).

4′-Chloro-2′-fluoro-2-methyl-[1,1′-biphenyl]-3-amine (**16i**)

Yellow oil. Yield, 99.2%. ^1^H NMR (300 MHz, DMSO-*d*_6_) δ 7.52 (dd, *J* = 9.8, 1.9 Hz, 1H, ArH), 7.37 (dd, *J* = 8.3, 2.0 Hz, 1H, ArH), 7.35–7.28 (m, 1H, ArH), 6.99 (t, *J* = 7.7 Hz, 1H, ArH), 6.72 (dd, *J* = 8.0, 1.4 Hz, 1H, ArH), 6.41 (dd, *J* = 7.5, 1.3 Hz, 1H, ArH), 5.03(s, 2H, NH_2_), 1.85 (d, *J* = 1.8 Hz, 3H, ArCH_3_).

2. ′-Fluoro-2-methyl-4′-(trifluoromethyl)-[1,1′-biphenyl]-3-amine (**16j**)

Yellow oil. Yield, 80.6%. ^1^H NMR (400 MHz, DMSO-*d*_6_) δ 7.74 (dd, *J* = 9.8, 2.1 Hz, 1H, ArH), 7.64 (dd, *J* = 8.0, 2.1 Hz, 1H, ArH), 7.51 (t, *J* = 7.6 Hz, 1H, ArH), 6.99 (t, *J* = 7.8 Hz, 1H, ArH), 6.73 (d, *J* = 8.0 Hz, 1H, ArH), 6.42 (dd, *J* = 7.5, 1.4 Hz, 1H, ArH), 5.04 (bs, 2H, NH_2_), 1.84 (d, *J* = 1.9 Hz, 3H, ArCH_3_).

#### 3.1.9. The Synthesis of Compounds **17a–j**

Compounds **17a–j** were synthesized by the following procedure. HCl (24.84 mL, 3 M) was added to a solution of **16a** (4.00 g, 19.9 mmol) in MeOH (40 mL), followed by the addition of H_2_O (20 mL) at room temperature. The mixture was stirred for 30 min, and NaNO_2_ (9.92 mL, 2.2 M) was added dropwise very slowly at 0–5 °C. After being stirred for another 30 min, B_2_pin_2_ (15.12 g, 59.7 mmol) in MeOH (40 mL) was added to the mixture solution and warmed to room temperature for 2 h. Then the mixture was extracted with DCM (50 mL × 3), washed with brine (50 mL × 2), and dried over anhydrous Na_2_SO_4_. The organic layer was concentrated in vacuum and purified by column chromatography on silica gel (petroleum ether/ethyl acetate = 250/1–150/1) to the target compound.

2-(2′-Fluoro-2-methyl-[1,1′-biphenyl]-3-yl)-4,4,5,5-tetramethyl-1,3,2-dioxaborolane (**17a**)

Yellow solid. Yield, 60.4%. m.p. 94.0–95.0 °C. ^1^H NMR (300 MHz, DMSO-*d*_6_) δ 7.70 (dd, *J* = 5.4, 3.7 Hz, 1H, ArH), 7.53–7.36 (m, 1H, ArH), 7.34–7.25 (m, 5H, ArH), 2.27 (d, *J* = 1.4 Hz, 3H, ArCH_3_), 1.31 (s, 12H, CH_3_).

2-(2,2′-Dimethyl-[1,1′-biphenyl]-3-yl)-4,4,5,5-tetramethyl-1,3,2-dioxaborolane (**17b**)

Colorless oil. Yield, 40.1%. ^1^H NMR (300 MHz, DMSO-*d*_6_) δ 7.67 (dd, *J* = 7.4, 1.8 Hz, 1H, ArH), 7.34–7.21 (m, 4H, ArH), 7.15 (dd, *J* = 7.5, 1.8 Hz, 1H, ArH), 7.10–6.95 (m, 1H, ArH), 2.17 (s, 3H, ArCH_3_), 1.99 (s, 3H, ArCH_3_), 1.33 (s, 12H, CCH_3_).

2-(2′-Chloro-2-methyl-[1,1′-biphenyl]-3-yl)-4,4,5,5-tetramethyl-1,3,2-dioxaborolane (**17c**)

Light yellow solid. Yield, 62.9%. m.p. 101.0–103.0 °C. ^1^H NMR (300 MHz, DMSO-*d*_6_) δ 7.71 (dd, *J* = 7.3, 1.8 Hz, 1H, ArH), 7.60 (dd, *J* = 5.8, 3.5 Hz, 1H, ArH), 7.48–7.41 (m, 2H, ArH), 7.33–7.25 (m, 2H, ArH), 7.21 (dd, *J* = 7.6, 1.8 Hz, 1H, ArH), 2.22 (s, 3H, ArCH_3_), 1.34 (s, 12H, CH_3_).

4,4,5,5-Tetramethyl-2-(2-methyl-2′-(trifluoromethyl)-[1,1′-biphenyl]-3-yl)-1,3,2-dioxaborolane (**17d**)

Colorless oil. Yield, 68.8%. ^1^H NMR (300 MHz, DMSO-*d*_6_) δ 7.83 (dd, *J* = 7.9, 1.6 Hz, 1H, ArH), 7.79–7.70 (m, 2H, ArH), 7.68–7.61 (m, 1H, ArH), 7.29 (d, *J* = 7.5 Hz, 1H), 7.25–7.13 (m, 2H, ArH), 2.12 (s, 3H, ArH), 1.31 (s, 12H, CH_3_).

4,4,5,5-Tetramethyl-2-(2-methyl-[1,1′-biphenyl]-3-yl)-1,3,2-dioxaborolane (**17e**)

Colorless oil. Yield, 55.3%. ^1^H NMR (400 MHz, DMSO-*d*_6_) δ 7.69 (d, *J* = 7.8 Hz, 1H, ArH), 7.57–7.40 (m, 1H, ArH), 7.39–7.20 (m, 4H, ArH), 7.03 (s, 1H, ArH), 6.99 (d, *J* = 7.9 Hz, 1H, ArH), 2.26 (d, *J* = 1.3 Hz, 3H, ArCH_3_), 1.32 (s, 12H, CH_3_).

2-(2′-Fluoro-2-methyl-[1,1′-biphenyl]-3-yl)-4,4,5,5-tetramethyl-1,3,2-dioxaborolane (**17f**)

White solid. Yield, 56.8%. ^1^H NMR (300 MHz, DMSO-*d*_6_) δ 7.69 (dd, *J* = 6.4, 2.5 Hz, 1H, ArH), 7.50 (td, *J* = 8.1, 6.2 Hz, 1H, ArH), 7.34–7.26 (m, 2H, ArH), 7.26–7.21 (m, 1H, ArH), 7.19–7.16 (m, 2H, ArH), 2.37 (s, 3H, ArCH_3_), 1.34 (s, 12H, CH_3_).

2-(4′-Fluoro-2-methyl-[1,1′-biphenyl]-3-yl)-4,4,5,5-tetramethyl-1,3,2-dioxaborolane (**17g**)

Yellow solid. Yield, 60.0%. ^1^H NMR (300 MHz, DMSO-*d*_6_) δ 7.71–7.63 (m, 1H, ArH), 7.38–7.34 (m, 1H, ArH), 7.34–7.29 (m, 2H, ArH), 7.29–7.23 (m, 3H, ArH), 2.35 (s, 3H, ArCH_3_), 1.33 (s, 12H, CH_3_).

2-(2′,4′-Difluoro-2-methyl-[1,1′-biphenyl]-3-yl)-4,4,5,5-tetramethyl-1,3,2-dioxaborolane (**17h**)

Light yellow solid. Yield, 67.5%. m.p. 59.5–61.5 °C. ^1^H NMR (300 MHz, DMSO-*d*_6_) δ 7.79–7.68 (m, 1H, ArH), 7.43–7.32 (m, 2H, ArH), 7.28 (d, *J* = 4.4 Hz, 2H, ArH), 7.19 (td, *J* = 8.5, 2.7 Hz, 1H, ArH), 2.28 (d, *J* = 1.3 Hz, 3H, ArCH_3_), 1.33 (s, 12H, CH_3_).

2-(2′,4′-Difluoro-2-methyl-[1,1′-biphenyl]-3-yl)-4,4,5,5-tetramethyl-1,3,2-dioxaborolane (**17i**)

Light yellow solid. Yield, 63.5%. m.p. 91.5–94.5 °C. ^1^H NMR (300 MHz, DMSO-*d*_6_) δ 7.72 (q, *J* = 4.0 Hz, 1H, ArH), 7.57 (dd, *J* = 9.8, 2.0 Hz, 1H, ArH), 7.41 (dd, *J* = 8.2, 2.0 Hz, 1H, ArH), 7.37 (d, *J* = 7.7 Hz, 1H, ArH), 7.30 (d, *J* = 4.5 Hz, 2H, ArH), 2.28 (s 3H, ArCH_3_), 1.34 (s, 12H, CH_3_).

2-(2′-Fluoro-2-methyl-4′-(trifluoromethyl)-[1,1′-biphenyl]-3-yl)-4,4,5,5-tetramethyl-1,3,2-dioxaborolane (**17j**)

Brown solid. Yield, 63.5%. m.p. 76.5–78.5 °C. ^1^H NMR (400 MHz, DMSO-*d*_6_) δ 7.80 (d, *J* = 9.8Hz, 1H, ArH), 7.74 (d, *J* = 6.3Hz, 1H, ArH), 7.70–7.66 (m, 1H, ArH), 7.59–7.52 (m, 1H, ArH), 7.33–7.28 (m, 2H, ArH), 2.27 (s, 3H, CH_3_), 1.31 (s, 12H, CH_3_).

#### 3.1.10. 2″-Fluoro-3-Methoxy-2′-Methyl-[1,1′:3′,1″-Terphenyl]-4-Carbaldehyde (**19a**)

To a solution of **17** (2.50 g, 8.01 mmol), **18a** (1.51 g, 8.81 mmol), and K_2_CO_3_ (3.10 g, 22.4 mmol) in 1,4-dioxane/water (25 mL/2.5 mL), Pd(PPh_3_)_4_ (0.78 g, 0.67 mmol) was added, and the mixture was allowed to stir at 90 °C for 8 h under N_2_ atmosphere. The undissolved solid was filtered off, and the filtrate was concentrated under vacuum. The residue was dissolved in ethyl acetate (25 mL × 3) and washed with water (25 mL × 2), followed by brine (25 mL × 2). The organic layer was dried over anhydrous Na_2_SO_4_, filtered, and concentrated in vacuum. The residue was purified by silica gel chromatography (Petroleum ether/ ethyl acetate = 150/1–80/1) to afford compound **19a** (white solid 2.45 g, yield: 95.3%). m.p. 82.0–83.5 °C. ^1^H NMR (400 MHz, DMSO-d_6_) δ 10.39 (s, 1H, CHO), 7.77 (d, *J* = 7.9 Hz, 1H, ArH), 7.50–7.43 (m, 1H, ArH), 7.41–7.36 (m, 2H, ArH), 7.36–7.30 (m, 3H, ArH), 7.30–7.24 (m, 1H, ArH), 7.20 (s, 1H, ArH), 7.08 (dt, *J* = 7.9, 1.0 Hz, 1H, ArH), 3.96 (s, 3H, OCH_3_), 2.21 (s, 3H, ArCH_3_).

#### 3.1.11. The Synthesis of Compounds **19b–k**

Compounds **19b**–**k** were prepared analogously to compound **16a**.

6-(2′-Fluoro-2-methyl-[1,1′-biphenyl]-3-yl)-2-methoxynicotinaldehyde (**19b**)

White solid powder. Yield, 72.1%. m.p. 78.5–81.5 °C. ^1^H NMR (300 MHz, DMSO-*d*_6_) δ 10.32 (s, 1H, CHO), 8.22 (d, *J* = 7.7 Hz, 1H, ArH), 7.59–7.54 (m, 1H, ArH), 7.51 (d, *J* = 7.8 Hz, 1H, ArH), 7.46 (d, *J* = 7.3 Hz, 1H, ArH), 7.42 (d, *J* = 5.4 Hz, 1H, ArH), 7.40–7.31 (m, 4H, ArH), 4.07 (s, 3H, OCH_3_), 2.18 (s, 3H, ArCH_3_).

6-(2,2′-Dimethyl-[1,1′-biphenyl]-3-yl)-2-methoxynicotinaldehyde (**19c**)

Colorless oil. Yield, 74.5%. ^1^H NMR (400 MHz, DMSO-*d*_6_) δ 10.30 (s, 1H, CHO), 8.19 (d, *J* = 7.7 Hz, 1H, ArH), 7.50 (dd, *J* = 7.8, 1.5 Hz, 1H, ArH), 7.42–7.36 (m, 2H, ArH), 7.35–7.24 (m, 3H, ArH), 7.20 (dd, *J* = 7.5, 1.5 Hz, 1H, ArH), 7.14 (dd, *J* = 6.9, 2.0 Hz, 1H, ArH), 4.04 (s, 3H, OCH_3_), 2.05 (s, 3H, ArCH_3_), 2.04 (s, 3H, ArCH3).

6-(2′-Chloro-2-methyl-[1,1′-biphenyl]-3-yl)-2-methoxynicotinaldehyde (**19d**)

Colorless oil, Yield, 61.7%. ^1^H NMR (300 MHz, DMSO-*d*_6_) δ 10.32 (s, 1H, CHO), 8.22 (d, *J* = 7.7 Hz, 1H, ArH), 7.65–7.61 (m, 1H, ArH), 7.57 (dd, *J* = 7.8, 1.7 Hz, 1H, ArH), 7.54–7.46 (m, 2H, ArH), 7.46–7.37 (m, 3H, ArH), 7.28 (dd, *J* = 7.6, 1.5 Hz, 1H, ArH), 4.07 (s, 3H, OCH_3_), 2.12 (s, 3H, ArCH_3_).

2-Methoxy-6-(2-methyl-2′-(trifluoromethyl)-[1,1′-biphenyl]-3-yl)nicotinaldehyde (**19e**)

White solid. Yield, 48.4%. ^1^H NMR (300 MHz, DMSO-*d*_6_) δ 10.31 (s, 1H, CHO), 8.20 (d, *J* = 7.7 Hz, 1H, ArH), 7.89 (d, *J* = 6.6 Hz, 1H, ArH), 7.77 (t, *J* = 6.7 Hz, 1H, ArH), 7.66 (t, *J* = 7.7 Hz, 1H, ArH), 7.56 (dd, *J* = 7.8, 1.5 Hz, 1H, ArH), 7.52–7.43 (m, 3H, ArH), 7.25 (d, *J* = 7.8 Hz, 1H, ArH), 4.05 (s, 3H, OCH_3_), 2.03 (s, 3H, ArCH_3_).

2-Methoxy-6-(2-methyl-[1,1′-biphenyl]-3-yl)nicotinaldehyde (**19f**)

White solid. Yield, 56.8%. ^1^H NMR (300 MHz, DMSO-*d*_6_) δ 10.42 (s, 1H, CHO), 7.79 (d, *J* = 7.8 Hz, 1H, ArH), 7.54–7.45 (m, 1H, ArH), 7.44–7.25 (m, 6H, ArH), 7.23 (s, 1H, ArH), 7.10 (d, *J* = 7.9 Hz, 1H, ArH), 3.99 (s, 3H, OCH_3_), 2.04 (s, 3H, ArCH_3_).

6-(3′-Fluoro-2-methyl-[1,1′-biphenyl]-3-yl)-2-methoxynicotinaldehyde (**19g**)

White solid. Yield, 68.8%. m.p. 131.0–132.0 °C. ^1^H NMR (300 MHz, DMSO-*d*_6_) δ 10.31 (s, 1H, CHO), 8.22 (dd, *J* = 8.2, 5.4 Hz, 1H, ArH), 7.52 (dq, *J* = 8.9, 3.7, 3.1 Hz, 2H, ArH), 7.46–7.31 (m, 3H, ArH), 7.26 (t, *J* = 7.5 Hz, 3H, ArH), 4.06 (s, 3H, OCH_3_), 2.24 (s, 3H, ArCH_3_).

6-(3′-Fluoro-2-methyl-[1,1′-biphenyl]-3-yl)-2-methoxynicotinaldehyde (**19h**)

White solid. Yield, 72.3%. m.p. 46.0–48.0 °C. ^1^H NMR (300 MHz, DMSO-*d*_6_) δ 10.18 (s, 1H, ArH), 8.12 (d, *J* = 7.9 Hz, 1H, ArH), 7.64 (dd, *J* = 5.7, 3.3 Hz, 1H, ArH), 7.42–7.10 (m, 7H, ArH), 4.01 (s, 3H, OCH_3_), 2.33 (s, 3H, ArCH_3_).

6-(2′,4′-Difluoro-2-methyl-[1,1′-biphenyl]-3-yl)-2-methoxynicotinaldehyde (**19i**)

White solid. Yield, 20.8%. m.p. 119.5–222.0 °C. ^1^H NMR (400 MHz, DMSO-*d*_6_) δ 10.30 (s, 1H, CHO), 8.20 (d, *J* = 7.6 Hz, 1H, ArH), 7.54 (dd, *J* = 7.8, 1.6 Hz, 1H, ArH), 7.48–7.41(m, 2H, ArH), 7.40–7.35 (m, 2H, ArH), 7.33 (dd, *J* = 7.6, 1.6 Hz, 1H, ArH), 7.22 (td, *J* = 8.5, 2.3 Hz, 1H, ArH), 4.04 (s, 3H, OCH_3_), 2.14 (s, 3H, ArCH_3_).

6-(4′-Chloro-2′-fluoro-2-methyl-[1,1′-biphenyl]-3-yl)-2-methoxynicotinaldehyde (**19j**)

White solid. Yield, 29.8%. m.p. 132.0–134.0 °C. ^1^H NMR (400 MHz, DMSO-*d*_6_) δ 10.30 (s, 1H, CHO), 8.20 (d, *J* = 7.8 Hz, 1H, ArH), 7.61–7.53 (m, 2H, ArH), 7.47–7.40 (m, 3H, ArH), 7.37 (d, *J* = 7.6 Hz, 1H, ArH), 7.34 (dd, *J* = 7.6, 1.6 Hz, 1H, ArH), 4.04 (s, 3H, OCH_3_), 2.14 (s, 3H, ArCH_3_).

6-(2′-Fluoro-2-methyl-4′-(trifluoromethyl)-[1,1′-biphenyl]-3-yl)-2-methoxy nicotinaldehyde (**19k**)

White solid. Yield, 47.5%. m.p. 62.0–64.0 °C. ^1^H NMR (400 MHz, DMSO-*d*_6_) δ 10.30 (s, 1H, CHO), 8.20 (d, *J* = 7.6 Hz, 1H, ArH), 7.84 (dd, *J* = 9.9, 1.9 Hz, 1H, ArH), 7.74–7.69 (m, 1H, ArH), 7.65 (t, *J* = 7.6 Hz, 1H, ArH), 7.58 (dd, *J* = 7.6, 1.6 Hz, 1H, ArH), 7.46 (t, *J* = 7.5 Hz, 1H, ArH), 7.41-7.32 (m, 2H, ArH), 4.04 (s, 3H, OCH_3_), 2.15 (s, 3H, ArCH_3_).

#### 3.1.12. The Synthesis of Compounds **A1** and **A2**

Compounds **A1** and **A2** were prepared analogous to compounds **16a**–**j**.

1-(3-((2,2′-Dimethyl-[1,1′-biphenyl]-3-yl)oxy)propyl)piperidin-4-ol hydrochloride (**A1**).

White solid. Yield, 82.0%. m.p. 118.0–201.0 °C. ^1^H NMR (400 MHz, DMSO-*d*_6_) δ 7.27 (dd, *J* = 6.6, 1.5 Hz, 2H, ArH), 7.26–7.22 (m, 1H, ArH), 7.19 (t, *J* = 7.8 Hz, 1H, ArH), 7.09–7.00 (m, 1H, ArH), 6.94 (d, *J* = 8.1 Hz, 1H, ArH), 6.66 (d, *J* = 7.4 Hz, 1H, ArH), 4.60 (s, 1H, OH), 4.09–3.99 (m, 3H, OCH_3_), 3.54–3.42 (m, 2H, NCH_2_), 2.87–2.72 (m, 2H, NCH_2_-piperidinol), 2.14 (m, 2H, CH_2_CH_2_CH_2_), 1.99 (s, 3H, ArCH_3_), 1.94 (s, 2H, NCH_2_-piperidinol), 1.83 (s, 3H, ArCH_3_), 1.73 (s, 2H, CH_2_-piperidinol), 1.44 (s, 2H, CH_2_-piperidinol). ^13^C NMR (75 MHz, DMSO-*d*_6_) δ 156.82, 142.68, 141.40, 135.61, 130.22, 129.45, 127.72, 126.74, 126.13, 124.03, 121.91, 110.52, 65.62, 60.03, 50.72, 47.37, 31.89, 29.75, 19.94(2C), 13.21(2C). ESI-HRMS: *m*/*z* [M+H]^+^calculated for C_22_H_31_NO_2_, 340.2277; Found: 340.2268. LCMS: *t*_R_ 9.97 min, purity, 95.1%.

1′-((2′-Fluoro-2-methyl-[1,1′-biphenyl]-3-yl)methyl)-[1,4′-bipiperidin]-4-ol (**A2**).

Light yellow powder. Yield, 89.3%. m.p. 110.0–113.0 °C. ^1^H NMR (300 MHz, DMSO-*d*_6_) δ 7.52–7.41 (m, 1H, ArH), 7.36–7.25 (m, 4H, ArH), 7.23 (d, *J* = 7.5 Hz, 1H, ArH), 7.11 (dd, *J* = 7.5, 1.6 Hz, 1H, ArH), 4.54 (s, 1H, CHOH), 3.46 (s, 3H, ArCH_2_N/ CHOH), 2.89 (d, *J* = 11.0 Hz, 2H, NCH_2_-piperidine), 2.83–2.69 (m, 2H, NCH_2_-piperidine), 2.36–2.15 (m, 3H, CHN, NCH_2_-piperidine), 2.12 (s, 3H, CH_3_), 1.99 (t, *J* = 10.7 Hz, 2H, NCH_2_-piperidine), 1.82–1.61 (m, 4H, CH_2_-piperidine), 1.57–1.18 (m, 4H, CH_2_-piperidine).^13^C NMR (75 MHz, DMSO-*d*_6_) δ 161.07, 157.84, 137.64, 136.18, 135.79, 132.11, 129.81, 129.14, 125.51, 124.96, 116.01, 115.72, 67.13, 61.95, 61.08, 53.49(2C), 47.13(2C), 35.25(2C), 28.32(2C), 16.09. ESI-HRMS: *m*/*z* [M+H]^+^calculated for C_24_H_32_FN_2_O, 383.2499 ; Found: 383.2495. LCMS: *t*_R_ 10.69 min, purity, 96.5%.

#### 3.1.13. The Synthesis of Compounds **A3**–**A8**

Compounds **A3**–**A8** were synthesized by the following procedure. A mixture of **19a** or **19b** (0.92 mmol), amide (1.11 mmol), and HAc (0.11 mL, 1.84 mmol) in DCM/MeOH (5 mL/1 mL) was stirred for 4 h at room temperature. NaBH(OAc)_3_ (2.30 mmol) was added to the mixture slowly at 0 °C and stirred for another 8 h. The reaction was adjusted to pH 7–8 with saturated NaHCO_3_ aqueous solution. The mixture was extracted with DCM (10 mL × 3) and washed with brine (10 mL × 3). The combined organic layer was dried over anhydrous Na_2_SO_4_, filtered, and concentrated in vacuum. The residue was purified by silica gel chromatography to afford the product.

1-((2″-Fluoro-3-methoxy-2′-methyl-[1,1′:3′,1″-terphenyl]-4-yl)methyl)piperidin-4-ol (**A3**).

White solid. Yield, 66.4%. m.p. 61.5–63.5 °C. ^1^H NMR (400 MHz, DMSO-*d*_6_) δ 7.52–7.43 (m, 1H, ArH), 7.41–7.33 (m, 3H, ArH), 7.32–7.26 (m, 3H, ArH), 7.21 (dd, *J* = 7.3, 1.9 Hz, 1H, ArH), 6.92 (d, *J* = 8.4 Hz, 2H, ArH), 4.57 (s, 1H, OH), 3.81 (s, 3H, OCH_3_), 3.47 (br, 3H, ArCH_2_/piperidine-CH), 2.75 (b r, 2H, piperidine-CH_2_), 2.11 (b r, 2H, piperidine-CH_2_), 2.01 (s, 3H, ArCH_3_), 1.79–1.63 (m, 2H, piperidine-CH_2_), 1.50–1.36 (m, 2H, piperidine-CH_2_). ^13^C NMR (75 MHz, DMSO-*d*_6_) δ 161.08, 157.85(F-C), 157.45(F-C), 142.66, 141.71, 136.56, 132.13(d), 132.08, 130.12(d), 129.93, 129.51, 129.29, 126.04, 125.25, 125.02(d), 121.37, 116.12, 115.83, 112.12, 66.71, 55.85 (2C), 55.73, 51.59, 34.82, 18.35 (2C). ESI-HRMS: *m*/*z* [M+H]^+^ calculated for C_26_H_28_FNO_2_, 406.2104; Found: 406.2177. LCMS: *t*_R_ 10.48 min, purity, 98.4%.

1-((6-(2′-Fluoro-2-methyl-[1,1′-biphenyl]-3-yl)-2-methoxypyridin-3-yl)methyl)piperidin-4-ol (**A4**).

White solid powder. Yield, 56.4%. m.p. 84.0–86.0 °C. ^1^H NMR (300 MHz, DMSO-*d*_6_) δ 7.76 (d, *J* = 7.5 Hz, 1H, ArH), 7.52 (dd, *J* = 7.6, 1.7 Hz, 1H, ArH), 7.42–7.28 (m, 5H, ArH), 7.24–7.16 (m, 1H, ArH), 7.09 (d, *J* = 7.3 Hz, 1H, ArH), 4.02 (s, 3H, OCH_3_), 3.77 (dt, *J* = 8.8, 4.5 Hz, 1H, CHOH), 3.61 (s, 2H, ArCH_2_N), 2.90 (dt, *J* = 10.8, 4.7 Hz, 2H, CH_2_-piperidine), 2.37–2.28 (m, 2H, CH_2_-piperidine), 2.27 (s, 3H, ArCH_3_), 2.05–1.96 (m, 2H, CH_2_-piperidine), 1.84 (br, 1H, OH) 1.78–1.68 (m, 2H, CH_2_-piperidine), 1.47–1.36 (m, 2H, CH_2_-piperidine). ^13^C NMR (75 MHz, Chloroform-*d*) δ 161.16, 155.99, 140.94, 138.50, 136.90, 134.92, 131.75, 130.08, 129.69, 129.58, 129.17, 129.06, 125.43, 124.00, 118.64, 117.24, 115.70, 115.40, 67.97, 55.53, 53.49 (2C), 51.19, 34.51 (2C),18.05. ESI-HRMS: *m*/*z* [M+H]^+^ calculated for C_26_H_29_FNO_2_, 407.2182; Found: 407.2137. LCMS: *t*_R_ 11.76 min, purity, 96.1%.

4-(((6-(2′-Fluoro-2-methyl-[1,1′-biphenyl]-3-yl)-2-methoxypyridin-3-yl)methyl)amino)cyclohexan-1-ol (**A5**).

White solid. Yield, 51.3%. m.p. 80.0–82.0 °C. ^1^H NMR (300 MHz, DMSO-*d*_6_) δ 7.82 (d, *J* = 7.5 Hz, 1H, ArH), 7.54–7.44 (m, 2H, ArH), 7.43–7.33 (m, 4H, ArH), 7.32–7.25 (m, 1H, ArH), 7.14 (d, *J* = 7.3 Hz, 1H, ArH), 4.52 (d, *J* = 4.5 Hz, 1H, OH), 3.90 (s, 3H, OCH_3_), 3.72 (s, 2H, CH_2_NH), 3.45–3.40 (m,1H, CHOH), 2.43–2.32 (m, 1H, NHCH), 2.13 (s, 3H, ArCH_3_), 1.94–1.78 (m, 4H, CH_2_-cyclohexane), 1.21–1.01 (m, 4H, CH_2_-cyclohexane). ^13^C NMR (75 MHz, DMSO-*d*_6_) δ 160.55, 157.88(F-C), 155.05(F-C), 141.03, 138.01, 136.75, 134.40, 132.12,130.12, 130.23, 129.95, 129.3(d), 126.05, 125.04, 122.11, 117.48, 116.11, 115.82, 69.29, 55.91, 53.59, 44.53, 34.32(2C), 31.26(2C), 18.17. ESI-HRMS: *m*/*z* [M+H]^+^ calculated for C_26_H_30_FN_2_O_2_, 421.2291; Found: 421.2284. LCMS: *t*_R_ 9.46 min, purity, 95.1%.

1-((6-(2′-Fluoro-2-methyl-[1,1′-biphenyl]-3-yl)-2-methoxypyridin-3-yl)methyl)piperidine-3-carboxamide (**A6**).

White solid powder. Yield, 41.0%. m.p. 88.0–91.0 °C. ^1^H NMR (300 MHz, DMSO-*d*_6_) δ 7.78 (d, *J* = 7.5 Hz, 1H, ArH), 7.55–7.44 (m, 2H, ArH), 7.42–7.33 (m, 4H, ArH, 1/2CONH_2_), 7.32–7.26 (m, 2H, ArH), 7.16 (d, *J* = 7.5 Hz, 1H), 6.82 (br, 1H, 1/2CONH_2_), 3.91 (s, 3H, OCH_3_), 3.50 (s, 2H, ArCH_2_), 2.89–2.71 (m, 2H, piperidine-CH_2_), 2.45–2.33 (m, 1H, piperidine-CHCO), 2.23–2.00 (m, 5H, ArCH_3_, piperidine-CH_2_), 1.84–1.62 (m, 2H, piperidine-CH_2_), 1.56–1.36 (m, 2H, piperidine-CH_2_). ^13^C NMR (75 MHz, DMSO-*d*_6_) δ 176.04, 161.00, 157.8(C-F), 155.51(C-F), 140.95, 139.15, 136.76, 134.41, 132.14, 130.28, 129.97, 129.39, 129.16, 126.07, 125.09, 119.21, 117.53, 115.9(d), 56.57, 56.05, 53.86, 53.65, 42.77, 27.54, 24.76, 18.19. ESI-HRMS: *m*/*z* [M+H]^+^ calculated for C_26_H_29_FN_3_O_2_, 434.2244; Found: 434.2238. LCMS: *t*_R_ 12.04 min, purity, 95.6%.

1-((6-(2′-Fluoro-2-methyl-[1,1′-biphenyl]-3-yl)-2-methoxypyridin-3-yl)methyl)piperidine-4-carboxamide (**A7**).

White solid powder. Yield, 44.7%. m.p. 194.0–196.0 °C. ^1^H NMR (300 MHz, DMSO-*d*_6_) δ 7.80 (d, *J* = 7.5 Hz, 1H, ArH), 7.53–7.45 (m, 2H, ArH), 7.43–7.34 (m, 3H, ArH), 7.33–7.26 (m, 2H, ArH), 7.25 (s, 1H, 1/2CONH_2_), 7.17 (d, *J* = 7.5 Hz, 1H, ArH), 6.75 (s, 1H, 1/2CONH_2_), 3.91 (s, 3H, OCH_3_), 3.54 (s, 2H, ArCH_2_), 2.91 (m, 2H, piperidine-CH_2_), 2.18–2.12 (s, 3H, ArCH_3_), 2.11–1.99 (m, 2H, piperidine-CH_2_), 1.78–1.54 (m, 4H, piperidine-CH_2_). ^13^C NMR (75 MHz, Chloroform-*d*) δ 177.63, 161.37, 161.16,158.16(C-F) 156.27(C-F), 140.83, 138.79, 136.90, 134.90, 131.76, 130.12, 129.74, 129.66, 129.52, 129.17(d), 125.43, 124.01, 117.29, 115.53(d), 55.64, 53.51(2C), 53.03, 42.38, 28.70(2C), 18.04. ESI-HRMS: *m*/*z* [M+H]^+^ calculated for C_26_H_29_FN_3_O_2_, 434.2244; Found: 434.2243. LCMS: *t*_R_ 10.05 min, purity, 96.3%.

1-((6-(2′-Fluoro-2-methyl-[1,1′-biphenyl]-3-yl)-2-methoxypyridin-3-yl)methyl)piperidine-4-carboxylic acid (**A8**).

White solid powder. Yield, 19.9%. m.p. 218.0–220.0 °C. ^1^H NMR (300 MHz, DMSO-*d*_6_) δ 12.27 (Br, 1H, COOH), 7.78 (d, *J* = 7.3 Hz, 1H, ArH), 7.53–7.44 (m, 2H, ArH), 7.43–7.32 (m, 4H, ArH), 7.32–7.24 (m, 1H, ArH), 7.15 (d, *J* = 7.3 Hz, 1H, ArH), 3.90 (s, 3H, OCH_3_), 3.49 (s, 2H, ArCH_2_N), 2.83 (d, *J* = 12.0 Hz, 2H, piperidine-CH_2_), 2.32–2.16 (m, 1H, CHCOOH), 2.17–2.03 (m, 5H, ArCH_3_/ piperidine-CH_2_), 1.83 (d, *J* = 13.0 Hz, 2H, piperidine-CH_2_), 1.68–1.54 (m, 2H, piperidine-CH_2_). ^13^C NMR (75 MHz, DMSO-*d*_6_) δ 176.78, 160.90, 157.85(C-F), 155.41(C-F), 140.94, 138.98, 136.75, 134.40, 132.16, 130.28, 130.16, 129.96, 129.35, 129.14, 126.08, 125.07(d), 119.20, 117.59, 115.97(d), 55.66, 53.65(2C), 53.16, 40.76, 28.62(2C),18.19. ESI-HRMS: *m*/*z* [M+H]^+^ calculated for C_26_H_28_FN_2_O_3_, 435.2084; Found: 435.2806. LCMS: *t*_R_ 6.52 min, purity, 99.1%.

#### 3.1.14. The Synthesis of Compounds **A9**–**A18**

Compounds **A9**–**A18** were synthesized by the following procedure. A mixture of aldehyde (0.92 mmol), β-alanine amide hydrochloride (1.11 mmol), and Et_3_N (1.84 mmol) in DCM/MeOH (5 mL/1 mL) was stirred for 12 h at room temperature. NaBH(OAc)_3_ (2.30 mmol) was added to the mixture slowly at 0 °C and stirred for another 8 h. The reaction was adjusted to pH 7–8 with saturated NaHCO_3_ aqueous solution. The mixture was extracted with DCM (10 mL × 3) and washed with brine (10 mL × 3). The combined organic layer was dried over anhydrous Na_2_SO_4_, filtered, and concentrated in vacuum. The residue was purified by silica gel chromatography to afford the product.

3-(((6-(2′-Fluoro-2-methyl-[1,1′-biphenyl]-3-yl)-2-methoxypyridin-3-yl)methyl)amino)propenamide (**A9**)

White solid powder. Yield, 54.1%. m.p. 218.0–222.0 °C. ^1^H NMR (300 MHz, DMSO-*d*_6_) δ 7.81 (d, *J* = 7.5 Hz, 1H, ArH), 7.52–7.45 (m, 2H, ArH), 7.45–7.33 (m, 5H, ArH, 1/2CONH_2_), 7.33–7.26 (m, 1H, ArH), 7.15 (d, *J* = 7.3 Hz, 1H, ArH), 6.82 (s, 1H, 1/2CONH_2_), 3.92 (s, 3H, OCH_3_), 3.72 (s, 2H, ArCH_2_), 2.77 (t, *J* = 6.8 Hz, 2H, NHCH_2_), 2.29 (t, *J* = 6.8 Hz, 2H, CH_2_CO), 2.14 (s, 3H, ArCH_3_). ^13^C NMR (75 MHz, DMSO-*d*_6_) δ 172.08, 161.02, 158.13(F-C), 157.85(F-C), 141.56, 140.48, 136.87, 134.42, 132.12, 130.69,130.34, 130.24, 129.97, 129.01(d), 126.21, 125.11, 117.70, 115.85, 113.26, 54.12, 44.95, 43.43, 30.87, 18.16. ESI-HRMS: *m*/*z* [M+H]^+^ calculated for C_23_H_25_FN_3_O_2_, 394.1931; Found: 394.1929. LCMS: *t*_R_ 8.19 min, purity, 96.7%.

3-(((6-(2,2′-Dimethyl-[1,1′-biphenyl]-3-yl)-2-methoxypyridin-3-yl)methyl)amino) propenamide (**A10**).

White solid powder. Yield, 55.5%. m.p. 70.0–73.0 °C. ^1^H NMR (400 MHz, DMSO-*d*_6_) δ 7.77 (d, *J* = 7.5 Hz, 1H, ArH), 7.42 (dd, *J* = 7.6, 1.6 Hz, 2H, ArH), 7.36–7.25 (m, 4H, ArH, 1/2CONH_2_), 7.17–7.10 (m, 3H, ArH), 6.82 (s, 1H, 1/2CONH_2_), 3.89 (s, 3H, OCH_3_), 3.88 (s, 1H, NH), 3.70 (s, 2H, ArCH_2_), 2.74 (t, *J* = 6.8 Hz, 2H, NHCH_2_CH_2_), 2.26 (t, *J* = 6.8 Hz, 2H, NHCH_2_CH_2_), 2.05 (s, 3H, ArCH_3_), 2.00 (s, 3H, ArCH_3_). ^13^C NMR (75 MHz, DMSO-*d*_6_) δ 174.03, 160.68, 155.56, 142.51, 141.86, 140.88, 138.20, 135.65, 133.61, 130.29, 129.58, 129.37, 129.18, 127.78, 126.25, 125.92, 120.84, 117.49, 53.58, 47.04, 45.50, 35.68, 20.10, 18.03. ESI-HRMS: *m*/*z* [M+H]^+^ calculated for C_24_H_28_N_3_O_2_, 390.2182; Found: 390.2183. LCMS: *t*_R_ 9.04 min, purity, 95.1%.

3-(((6-(2′-Chloro-2-methyl-[1,1′-biphenyl]-3-yl)-2-methoxypyridin-3-yl)methyl)amino)propenamide (**A11**).

White solid powder. Yield, 52.9%. m.p. 49.0–51.0 °C. ^1^H NMR (300 MHz, DMSO-*d*_6_) δ 7.81 (d, *J* = 7.5 Hz, 1H, ArH), 7.58–7.54 (m, 1H, ArH), 7.50 (s, 1H, 1/2CONH_2_), 7.46–7.40 (m, 3H, ArH), 7.40–7.31 (m, 2H, ArH), 7.24–7.02 (m, 2H, ArH), 6.89 (s, 1H, 1/2CONH_2_), 3.91 (s, 3H, OCH_3_), 3.74 (s, 2H, ArCH_2_), 2.80 (t, *J* = 6.8 Hz, 2H, NHCH_2_CH_2_), 2.33 (t, *J* = 6.7 Hz, 2H, NHCH_2_CH_2_), 2.07 (s, 3H, ArCH_3_). ^13^C NMR (75 MHz, DMSO-*d*_6_) δ 174.15, 160.70, 155.26, 140.83, 140.69, 140.38, 138.15, 134.20, 133.99, 132.82, 131.76, 129.87, 129.71, 129.59, 127.74, 125.94, 121.13, 117.52, 53.57, 47.11, 45.57, 35.80, 17.99. ESI-HRMS: *m*/*z* [M+H]^+^ calculated for C_23_H_25_ClN_3_O_2_, 410.1635; Found: 410.1633. LCMS: *t*_R_ 9.02 min, purity, 98.7%.

3-(((2-Methoxy-6-(2-methyl-2′-(trifluoromethyl)-[1,1′-biphenyl]-3-yl)pyridin-3-yl)methyl)amino)propenamide (**A12**).

White solid powder. Yield, 57.5%. m.p. 50.5–53.5 °C. ^1^H NMR (300 MHz, DMSO-*d*_6_) δ 7.85 (t, *J* = 7.5 Hz, 2H, ArH), 7.74 (t, *J* = 7.2 Hz, 1H, ArH), 7.63 (t, *J* = 7.7 Hz, 1H, ArH), 7.50 (s, 1H, 1/2CONH_2_), 7.46 (d, *J* = 7.7Hz, 1H, ArH), 7.40 (d, *J* = 7.3 Hz, 1H, ArH), 7.33 (t, *J* = 7.7 Hz, 1H, ArH), 7.15 (dd, *J* = 7.6, 5.2 Hz, 2H, ArH), 6.90 (s, 1H, 1/2CONH_2_), 3.90 (s, 3H, OCH_3_), 3.82 (s, 2H, ArCH_2_), 2.85 (t, *J* = 6.9 Hz, 2H, NHCH_2_CH_2_), 2.36 (t, *J* = 6.8 Hz, 2H, NHCH_2_CH_2_), 1.98 (s, 3H, ArCH_3_). ^13^C NMR (75 MHz, DMSO-*d*_6_) δ 173.58, 160.77, 155.86, 140.71, 140.43, 140.03, 138.98, 133.93, 132.84, 132.31, 129.91, 129.65, 128.58, 128.16(β-CF_3_), 127.77(β-CF_3_), 127.39(β-CF_3_), 127.00 (β-CF_3_), 126.47(CF_3_), 126.39, 125.26, 122.75(CF_3_), 120.61(CF_3_), 119.24, 117.59, 53.68, 46.60, 45.06, 34.75, 18.53. ESI-HRMS: *m*/*z* [M+H]^+^ calculated for C_24_H_25_F_3_N_3_O_2_, 444.1899; Found: 444.1894. LCMS: *t*_R_ 6.68 min, purity, 95.9%.

3-(((2-Methoxy-6-(2-methyl-[1,1′-biphenyl]-3-yl)pyridin-3-yl)methyl)amino)propenamide (**A13**).

White solid powder. Yield, 50.3%. m.p. 127.0–128.0 °C. ^1^H NMR (400 MHz, DMSO-*d*_6_) δ 7.78 (d, *J* = 7.4 Hz, 1H, ArH), 7.50–7.43 (m, 2H, ArH), 7.43–7.31 (m, 6H, ArH, 1/2CONH_2_), 7.24 (dd, *J* = 7.4, 1.7 Hz, 1H, ArH), 7.12 (d, *J* = 7.4 Hz, 1H, ArH), 6.81 (s, 1H, 1/2CONH_2_), 3.90 (s, 3H, OCH_3_), 3.71 (s, 2H, ArCH_2_), 2.74 (t, *J* = 6.8 Hz, 2H, NHCH_2_CH_2_), 2.26 (t, *J* = 6.8 Hz, 2H, NHCH_2_CH_2_), 2.18 (s, 3H, ArCH_3_). ^13^C NMR (100 MHz, Chloroform-*d*) δ 175.15, 161.09, 156.99, 143.21, 142.21, 140.93, 138.21, 133.65, 129.94, 129.42(2C), 128.91, 128.08(2C), 126.84, 125.44, 119.35, 117.15, 53.52, 48.30, 44.85, 35.16, 18.64. ESI-HRMS: *m*/*z* [M+H]^+^ calculated for C_23_H_26_N_3_O_2_, 376.2025; Found: 376.2022. LCMS: *t*_R_ 8.51 min, purity, 95.6%.

3-(((6-(3′-Fluoro-2-methyl-[1,1′-biphenyl]-3-yl)-2-methoxypyridin-3-yl)methyl)amino)propenamide (**A14**)

White solid powder. Yield, 48.6%. m.p. 48.0–51.0 °C. ^1^H NMR (300 MHz, DMSO-*d*_6_) δ 7.80 (d, *J* = 7.5 Hz, 1H, ArH), 7.58–7.48 (m, 1H, ArH), 7.48–7.41 (m, 3H, ArH, 1/2CONH_2_), 7.33–7.18 (m, 4H, ArH), 7.14 (d, *J* = 7.3 Hz, 1H, ArH), 6.81 (s, 1H, 1/2CONH_2_), 3.92 (s, 3H, OCH_3_), 3.71 (s, 2H, ArCH_2_), 2.76 (t, *J* = 6.9 Hz, 2H, NHCH_2_), 2.28 (t, *J* = 6.8 Hz, 2H, CH_2_CONH_2_), 2.21 (s, 3H, ArCH_3_). ^13^C NMR (75 MHz, DMSO-*d*_6_) δ 172.31, 164.00, 161.02, 160.77, 158.03, 144.37, 141.78, 141.33, 140.85, 133.33, 130.78, 130.19, 129.67, 126.24, 117.71, 116.33, 114.26, 113.69, 54.11, 45.81, 43.61, 31.17, 18.77. ESI-HRMS: *m*/*z* [M+H]^+^ calculated for C_23_H_25_FN_3_O_2_, 394.1931; Found: 394.1926. LCMS: *t*_R_ 4.95 min, purity, 96.1%.

3-(((6-(4′-Fluoro-2-methyl-[1,1′-biphenyl]-3-yl)-2-methoxypyridin-3-yl)methyl)amino)propenamide (**A15**)

White solid powder. Yield, 49.3%. m.p. 120.0–122.0 °C. ^1^H NMR (300 MHz, DMSO-*d*_6_) δ 7.82 (d, *J* = 7.5 Hz, 1H, ArH), 7.47–7.42 (m, 4H, ArH, 1/2CONH_2_), 7.40–7.25 (m, 4H, ArH), 7.15 (d, *J* = 7.5 Hz, 1H, ArH), 6.85 (s, 1H, 1/2CONH_2_), 3.93 (s, 3H, OCH_3_), 3.76 (s, 2H, ArCH_2_), 2.80 (t, *J* = 6.8 Hz, 2H, NHCH_2_CH_2_), 2.31 (t, *J* = 6.8 Hz, 2H, NHCH_2_CH_2_), 2.19 (s, 3H, ArCH_3_). ^13^C NMR (100 MHz, Chloroform-*d*) δ 175.34, 163.17(F-C), 161.08(F-C), 160.17, 156.78, 142.14, 141.07, 138.18(2C), 133.72, 130.96(2C), 129.93, 129.07, 125.50, 119.67, 117.11, 115.09, 114.85, 53.50, 48.27, 44.93, 35.32, 18.61. ESI-HRMS: *m*/*z* [M+H]^+^ calculated for C_23_H_25_FN_3_O_2_, 394.1931; Found: 394.1931. LCMS: *t*_R_ 8.47 min, purity, 95.6%.

3-(((6-(2′,4′-Difluoro-2-methyl-[1,1′-biphenyl]-3-yl)-2-methoxypyridin-3-yl)methyl)amino)propenamide (**A16**)

White solid powder. Yield, 43.9%. m.p. 113.0–115.0 °C. ^1^H NMR (300 MHz, DMSO-*d*_6_) δ 7.78 (d, *J* = 7.4 Hz, 1H, ArH), 7.49–7.43 (m, 1H, ArH), 7.43–7.39 (m, 2H, ArH), 7.39–7.33 (m, 2H, ArH), 7.27–7.16 (m, 2H, ArH, 1/2CONH_2_), 7.12 (d, *J* = 7.3 Hz, 1H, ArH), 6.80 (s, 1H, 1/2CONH_2_), 3.89 (s, 3H, OCH_3_), 3.64 (s, 2H, ArCH_2_), 2.74 (t, *J* = 6.8 Hz, 2H, NHCH_2_CH_2_CO), 2.26 (t, *J* = 6.8 Hz, 2H, NHCH_2_CH_2_CO), 2.10 (s, 3H, ArCH_3_). ^13^C NMR (100 MHz, DMSO-*d*_6_) δ 174.01, 160.71, 155.38, 141.04, 138.31, 135.85, 134.58, 133.03(d, 2C), 130.38, 130.10, 126.07, 125.59(d), 120.88, 117.46, 112.21(d), 104.38(d, 2C), 53.58, 47.00, 45.49, 35.60, 18.09. ESI-HRMS: *m*/*z* [M+H]^+^ calculated for C_23_H_24_F_2_N_3_O_2_, 412.1837; Found: 412.1831. LCMS: *t*_R_ 8.92 min, purity, 98.7%.

3-(((6-(4′-Chloro-2′-fluoro-2-methyl-[1,1′-biphenyl]-3-yl)-2-methoxypyridin-3-yl)methyl)amino)propenamide (**A17**)

White solid powder. Yield, 46.9%. m.p. 122.0–124.0 °C. ^1^H NMR (300 MHz, DMSO-*d*_6_) δ 7.80 (d, *J* = 7.5 Hz, 1H, ArH), 7.56 (d, *J* = 9.4 Hz, 1H, ArH), 7.50–7.37 (m, 5H, ArH,1/2CONH_2_), 7.25 (d, *J* = 7.5 Hz, 1H, ArH), 7.13 (d, *J* = 7.3 Hz, 1H, ArH), 6.83 (s, 1H, 1/2CONH_2_), 3.89 (s, 3H, OCH_3_), 3.73 (s, 2H, ArCH_2_), 2.77 (t, *J* = 5.9 Hz, 2H, NHCH_2_CH_2_), 2.28 (t, *J* = 6.2 Hz, 2H, NHCH_2_CH_2_), 2.11 (s, 3H, ArCH_3_). ^13^C NMR (75 MHz, DMSO-*d*_6_) δ 173.95, 161.05, 160.70, 157.77(F-C), 155.20(F-C), 141.08, 138.23, 135.61, 134.43, 133.69, 133.55, 133.37(d), 130.27, 128.33(d), 126.19, 125.35(d), 121.18, 117.51, 116.66(d), 53.63, 47.04, 45.55, 35.76, 18.15. ESI-HRMS: *m*/*z* [M+H]^+^ calculated for C_23_H_24_ClFN_3_O_2_, 428.1541; Found: 428.1537. LCMS: *t*_R_ 9.62 min, purity, 95.2%.

3-(((6-(2′-Fluoro-2-methyl-4′-(trifluoromethyl)-[1,1′-biphenyl]-3-yl)-2-methoxypyridin-3-yl)methyl)amino)propenamide (**A18**)

White solid powder. Yield, 50.9%. m.p. 137.0–139.0 °C. ^1^H NMR (300 MHz, DMSO-*d*_6_) δ 7.87–7.76 (m, 2H, ArH), 7.71 (dd, *J* = 8.5, 1.8 Hz, 1H, ArH), 7.64 (t, *J* = 7.6 Hz, 1H, ArH), 7.50 (dd, *J* = 7.7, 1.6 Hz, 1H, ArH), 7.45–7.37 (m, 2H, ArH, 1/2CONH_2_), 7.30 (dd, *J* = 7.6, 1.6 Hz, 1H, ArH), 7.13 (d, *J* = 7.3 Hz, 1H, ArH), 6.80 (s, 1H, 1/2CONH_2_), 3.89 (s, 3H, OCH_3_), 3.69(s, 2H, ArCH_2_), 2.74 (t, *J* = 6.8 Hz, 2H, NHCH_2_CH_2_), 2.26 (t, *J* = 6.8 Hz, 2H, NHCH_2_CH_2_), 2.12 (s, 3H, ArCH_3_). ^13^C NMR (100 MHz, Chloroform-*d*) δ 175.29, 161.15, 160.63(F-C),158.16(F-C), 156.22, 140.95, 138.15, 135.48, 134.68, 133.49(d), 132.47(d), 131.51(dd), 130.23, 127.44(CF_3_), 125.69, 124.72(CF_3_), 122.04(CF_3_), 121.03, 119.95,119.33(CF_3_), 117.17, 113.13(d), 53.52, 48.33, 44.90, 35.35, 18.00. ESI-HRMS: *m*/*z* [M+H]^+^ calculated for C_24_H_24_F_4_N_3_O_2_, 462.1805; Found: 462.1799. LCMS: *t*_R_ 9.72 min, purity, 96.8%.

### 3.2. Pharmacology

#### 3.2.1. HTRF-Based PD-1/PD-L1 Binding Assay

The PD-1/ PD-L1 binding assay kits (Cisbio, Codolet, France, Cat. no. 64ICP01PEG) were purchased from Cisbio. The experiments were performed according to the manufacturer’s guidelines. The HTRF assay used Perkin Elmer EnVision to read the fluorescence intensity at 665 nm and 615 nm and calculate the HTRF ratio (665 nm emission/615 nm emission). The IC_50_ values were calculated by four-parameter logistic curve in Graphpad prism.

#### 3.2.2. In Vitro Non-Specific Cytotoxicity Assay

The MDA-MB-231 and Jurkat cell line was provided by the Chinese Academy of Sciences Cell Bank. The cytotoxicity of the test compounds was determined using the MTT assay. Briefly, MDA-MB-231 and Jurkat cells were incubated at 37 °C in a humidified 5% CO_2_ incubator for 24 h in 96-microwell plates. Then, 100 μL of culture medium containing the test compounds at different concentrations was added to each well and incubated at 37 °C for another 48 h. The MTT was added and incubated for another 4 h, and the optical density was detected with a microplate reader at 570 nm. The IC_50_ values were calculated according to the dose-dependent curves.

#### 3.2.3. Binding Affinity Assay

SPR analysis was carried out according to the method described by a previous article [23]. The kinetic analysis for the interaction of small molecules with hPD-L1 was performed on a Biacore T200 (GE Healthcare Bio-sciences, Uppsala, Sweden) using Series S Sensor Chip Protein A (GE Healthcare Bio-sciences, Uppsala, Sweden, catalog # 29139131-AA). Briefly, Fc-hPD-L1 (Acro Biosystems, Cambridge, MA, USA, catalog #PD1-H5258) was diluted using 1 × PBS-P^+^ buffer (phosphate buffer 1×, GE Healthcare Bio-sciences, catalog # 28-9950-84, enriched with 0.05% surfactant P20) containing 5% DMSO (Sigma-Aldrich, St. Louis, MO, America, catalog # D4540) and captured as the ligand (i.e., the target of small molecules) on protein A chip surface following Biacore T200 build-in protocol for kinetic/affinity analysis at a concentration of 10 μg/mL. The contact time and flow rates were set as 120 s and 10 μL/min for the ligand capture. Each experiment was performed using a pair of flow cells, one with ligand captured and the other kept as reference. A concentration gradient of analytes was freshly prepared in 1× PBS-P^+^ buffer with 5% DMSO with at least five concentrations, and the concentration range was optimized according to different analytes. Analytes could flow through both ligand-captured flow cells and reference flow cells at the same rate (30 μL/min) and contact time (90 s). Solvent correction was included to avoid the impact of DMSO on surface plasmon effect during binding analysis. Extra washing of the flow system using 50% DMSO in 1×PBS-P^+^ buffer was allowed to control carry over effects. For the regeneration of surface after each cycle (one-time ligand capture and binding of analyte at a single concentration) was carried out using 10 mM glycine pH 1.5 (GE Healthcare Bio-sciences, Uppsala, Sweden, catalog #BR-1003-54) at a flow rate of 30 μL/min for 30 s. Kinetic analysis was performed at 25 °C. Maximum binding response (R_max_) and equilibrium dissociation rate constant (*K*_D_) values were calculated using Biacore T200 Evaluation software following the 1:1 binding model.

#### 3.2.4. IFN-γ Production Effect in T Cells Function Assay

The tumor/T cells coculture assay was conducted by ChemPartner [19]. Briefly, Hep3B cells were engineered to stably express OS-8 (anti-CD3 single chain variable fragment) and human PD-L1 (hPD-L1). Fresh PBMCs were isolated from a healthy donor by density gradient centrifugation. CD3^+^ T cells were isolated from fresh PBMCs by EasySep Human T Cells Isolation Kit (negative selection, STEMCELL Technologies, Vancouver, Canada). Hep3B-OS8- hPD-L1 cells were harvested and treated with 10 μg/mL mitomycin C at 37 °C for 1.5 h and washed 4 times with PBS. Hep3B-OS8-hPD-L1 and T cells (2.5 × 10^4^ in 50 μL and 5 × 10^4^ in 100 μL complete media, respectively) were added to the 96-well plates, followed by the addition of 4× final concentration of test compound in 50 μL complete media. The supernatants (150 μL) were harvested after 72 h of coculture to determine IFN-γ levels by ELISA.

### 3.3. Molecular Modelling

Molecular docking simulation was performed in the Schrödinger suite [28]. The molecules were washed and processed, the 3D conformation of small molecules were prepared by *LigPrep* module. The X-ray crystallographic structure of PD-L1 was obtained from the RCSB Protein Data Bank (PDB ID: 5J89). The protein was prepared to remove the water molecules and add hydrogens and minimized using *Protein Preparation Wizard* module. Then, the binding site was defined using the original ligand in the complex by *Receptor Grid Generation* module. The *Glide Docking* module in standard precision (SP) was used to investigate the interactions between compounds and PD-L1. Other parameters were set as the default. All the figures illustrating the molecular docking results were visualized and generated using the Maestro or Pymol software.

### 3.4. Water Solubility Studies

The aqueous solubility was estimated by HPLC. The HPLC analysis was performed on a Shimadzu LC-20AT machine with a BDS Hypersil C18 column, and the column temperature was at 28 °C. Mobile phase B (100% MeOH) and mobile phase A (100% water) were used in a gradient elution program (0 min: 30% (B), 2 min: 50% (B), 8 min: 95% (B), 14 min: 95% (B), 18 min: 70% (B), 20 min: 30% (B)) with a flow rate of 1.0 mL/min at 254 nM. Methanol was used to configure an accurate compounds concentration to calculate the standard curve. The excess test compound was dissolved in PBS (KH_2_PO_4_ 0.24 g + KCl 0.2 g + NaCl 8 g + Na_2_HPO_4_·12H_2_O 3.58 g + 1 L water) and then analyzed by HPLC.

## 4. Conclusions

The blockade of the PD-1/PD-L1 interaction by small molecules has been highly anticipated as a promising alternative or complementary therapeutic to mAbs in the field of cancer immunotherapy. Currently, the field of developing small-molecule inhibitors of PD-1/PD-L1 interaction is intensively explored and makes great progress. Moreover, several small molecules, derived from the skeleton of BMS-202, have entered clinical trials to treat advanced solid tumors. Although several co-crystal complexes of inhibitors binding to PD-L1 dimer have been reported, which provided a definite guide in the rational design of PD-L1 small molecular inhibitors, the highly hydrophobic PD-1/PD-L1 binding interface remain a challenge for designing anti-PD-1/PD-L1 small-molecule inhibitors.

In this research, a novel series of (2-methyl-[1,1′-biphenyl]-3-yl) pyridine derivatives were designed and synthesized as inhibitors targeting PD-1/PD-L1 pathways based on structural simplification strategy. The representative compound **A9** presents significantly improved potency in biochemical assay compared with BMS-202. Notably, the bioactivity of **A9** is equivalent to that of BMS-1058, but possesses lower MW, PSA, and higher LE value. Subsequently, we utilized SPR assay to measure the binding affinity and kinetics of **A9** to hPD-L1 (*K*_D_: 3.64 nM), and the results show that **A9** possesses the feature of fast association and slow dissociation rate. Furthermore, small-molecule inhibitor **A9** (5 μM) and mAbs Keytruda (5 μg/mL) show comparable promoting effect on production of INF-γ in a dose-dependent manner in Hep3B/OS-8/hPD-L1 and CD3-positive T cells co-culture assay, implying that **A9** may reverse hPD-1/hPD-L1 pathway-mediated immunosuppression. Taken together, these results suggest that **A9** is a promising inhibitor of PD-1/PD-L1 interaction and is worthy for further study.

## Figures and Tables

**Table 1:**
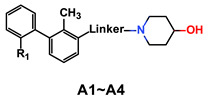
Activities of compounds **A1**–**A4** in inhibition of the PD-1/PD-L1 interaction.

Compd.	R_1_	Linker	Inhibitory Rate (%)		IC_50_ (nM) ^a^	MW	ALogP ^b^	PSA ^b^	LE ^c^
			1 μM	0.1 μM					
**A1**	CH_3_	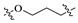	90	56	113.6	339.22	2.38	33.90	0.38
**A2**	F	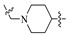	6.7	8.5	N.D.	382.24	2.09	27.91	ND
**A3**	F	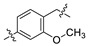	96.2	74.5	18.4	405.21	5.05	32.70	0.35
**A4**	F	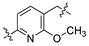	98.0	71.0	21.9	406.21	4.86	45.59	0.36
**BMS1058**					0.48 ^d^	753.33	4.04	132.41	0.24
**BMS202**					18.0	419.22	4.04	72.48	0.33

^a^ The data were generated from two independent experiments. ^b^ Estimated physicochemical parameters by Discovery Studio [29]. ^c^ The results were calculated by the equation: LE = −1.35 logIC_50_/Nheavy atoms, Nheavy atoms is the number of non-H atoms. ^d^ This IC_50_ value is taken from the patent [18].

**Table 2:**
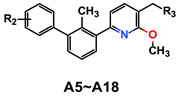
Activities of compounds **A5**–**A18** in inhibition of the PD-1/PD-L1 interaction.

Compd.	R_2_	R_3_	IC_50_ (nM) ^a^	MW	ALogP ^b^	PSA ^b^	LE ^c^
**A5**	2-F	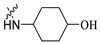	14.3	420.22	4.29	58.96	0.35
**A6**	2-F	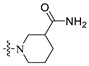	121.3	433.22	5.04	68.44	0.30
**A7**	2-F	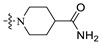	10.7	433.22	3.35	69.65	0.34
**A8**	2-F	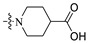	20.6	434.20	2.50	66.69	0.33
**A9**	2-F	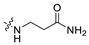	0.93	393.19	2.77	81.81	0.43
**A10**	2-CH_3_	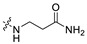	10.54	389.21	3.05	81.81	0.38
**A11**	2-Cl	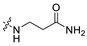	6.18	409.16	3.23	81.81	0.39
**A12**	2-CF_3_	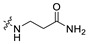	51.5	443.18	3.50	81.81	0.31
**A13**	H	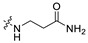	1.94	375.19	2.56	81.81	0.43
**A14**	3-F	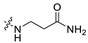	1.75	393.19	2.77	81.81	0.41
**A15**	4-F	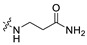	0.97	393.19	2.77	81.81	0.43
**A16**	2,4-di-F	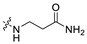	60.0	411.18	2.97	81.81	0.33
**A17**	4-Cl-2-F	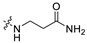	866.0	427.15	3.43	81.81	0.28
**A18**	4-CF_3_-2-F	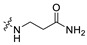	790.9	461.17	3.71	81.81	0.25

^a^ The data were generated from two independent experiments. ^b^ Estimated physicochemical parameters by Discovery Studio [29]. ^c^ The results were calculated by the equation: LE = −1.35 logIC_50_/N_heavy atoms_, N_heavy atoms_ is the number of non-H atoms.

**Table 3:** Cytotoxic of inhibitors of the PD-1/PD-L1 interaction.

Compound	EC_50_ (μM)/MDA-MB-231 Toxicity	EC_50_ (μM)/Jurkat Toxicity
**A9**	11.1	7.6
**A11**	7.1	5.5
**A13**	>10	7.73
**A14**	>10	>10
**A15**	10.2	5.5

## Data Availability

The article contains complete data used to support the findings of this study.

## Supplementary Materials

The following are available online, Table S1: The Kinetic Parameters and Binding Affinities of compounds **A7**, **A9**, **A11** and **A15** with hPD-L1.

## Sample Availability

Samples of the compounds described in this paper are available from the authors.
