# Supplementary material for: Discovery of Novel Small-Molecule Inhibitors of PD-1/PD-L1 Interaction via Structural Simplification Strategy
Source: Molecules. 2021 Jun 2;26(11):3347. doi: 10.3390/molecules26113347 (PMC8199613; doi:10.3390/molecules26113347)
Supplement: Supplementary file 1 [file molecules-26-03347-s001.zip › molecules-1227347-supplementary.pdf]

Supporting Information

Discovery of novel small-molecule inhibitors of PD-1/PD-L1 interaction via structural simplification strategy

Table of Contents

I. Supplementary Tables S3

Table S1 ....S3

II. Spectroscopic data.. S4

III. HPLC Spectroscopic data for A9 .... S Error! Bookmark not defined.

I. Supplementary Table

Table S1. The Kinetic Parameters and Binding Affinities of compounds with hPD-L1.

Compound	K_a ($M^{-1}s^{-1}$)	K_d (s^{-1})	K_D (M)	LE[a]
A7	7.89×10^{5}	6.38×10^{-3}	8.09×10^{-9}	0.35
A9	5.38×10^{5}	1.96×10^{-3}	3.64×10^{-9}	0.40
A11	5.76×10^{5}	3.68×10^{-3}	6.39×10^{-9}	0.39
A15	5.05×10^{5}	1.01×10^{-2}	1.99×10^{-8}	0.36

[a] The result is calculated by the equation: LE = -1.35log $K_D/N_{heavy\ atoms}$, $N_{heavy\ atoms}$ is the number of non-H atoms.

II. Spectroscopic data of compounds A1–18

^{1}H NMR Spectra for Compound **A1**

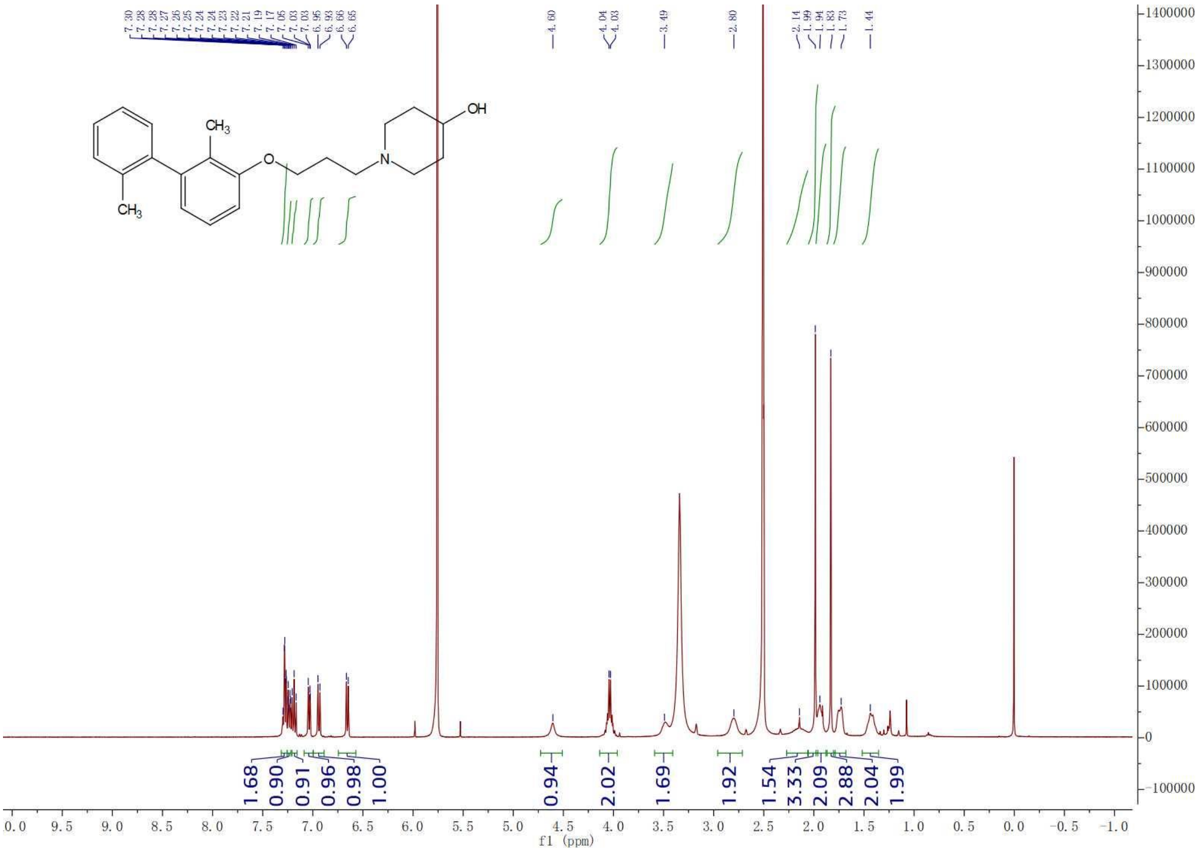

LCMS Spectra for Compound **A1**

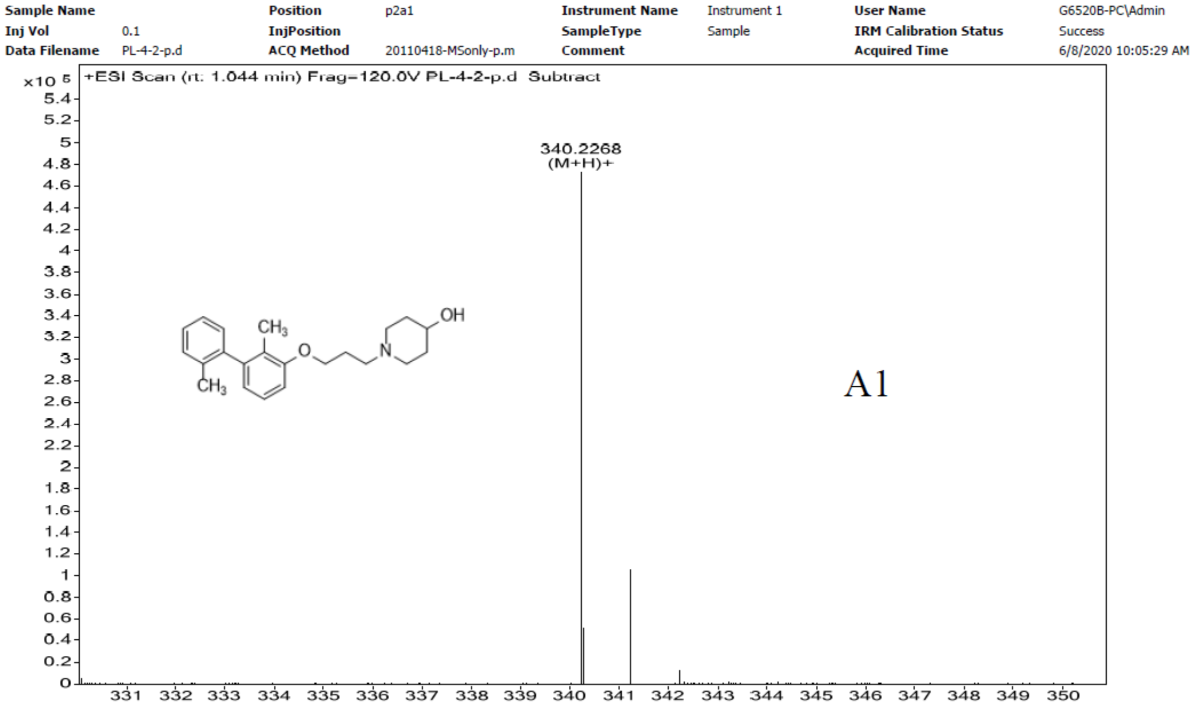

^{13}C NMR Spectra for Compound **A1**

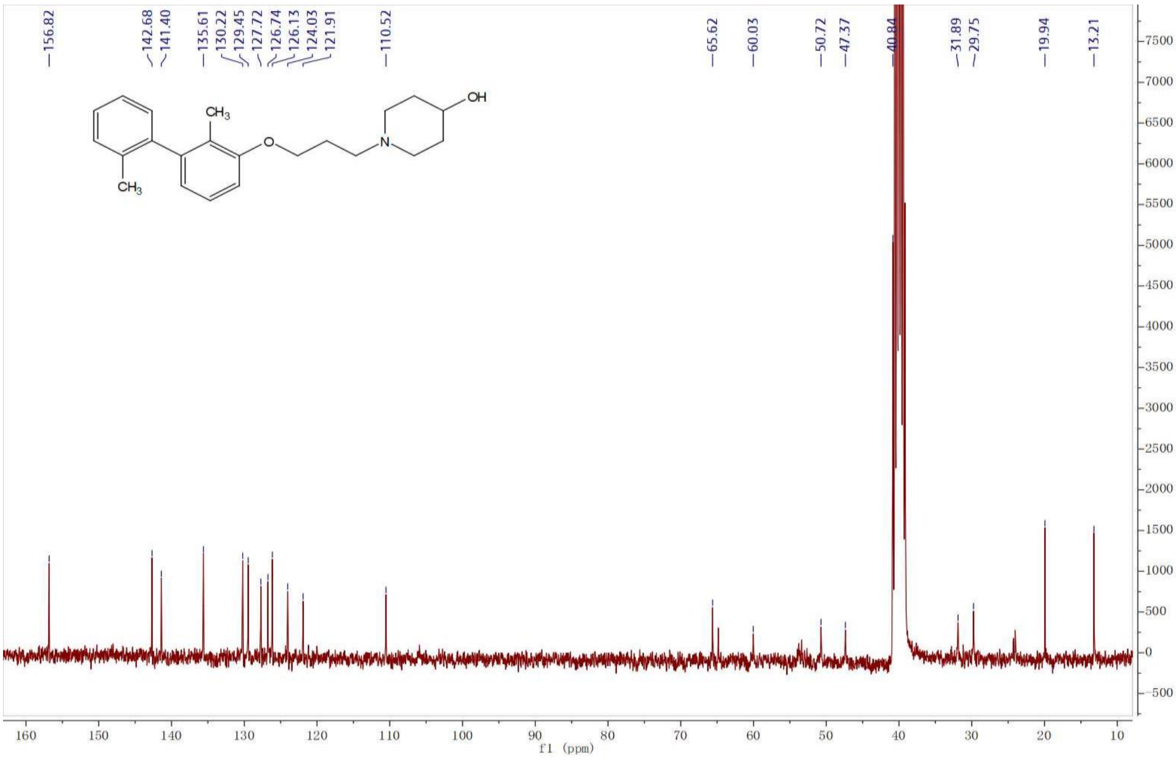

^{1}H NMR Spectra for Compound **A2**

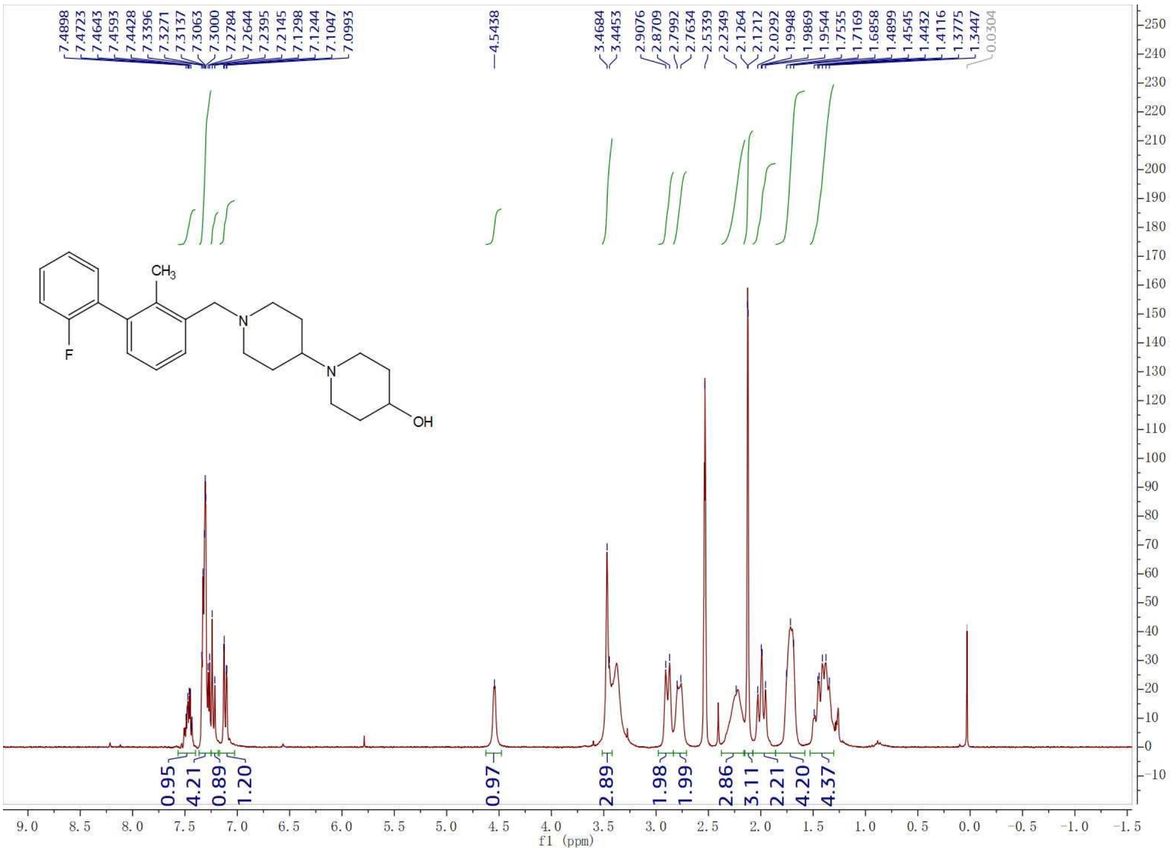

LCMS Spectra for Compound **A2**

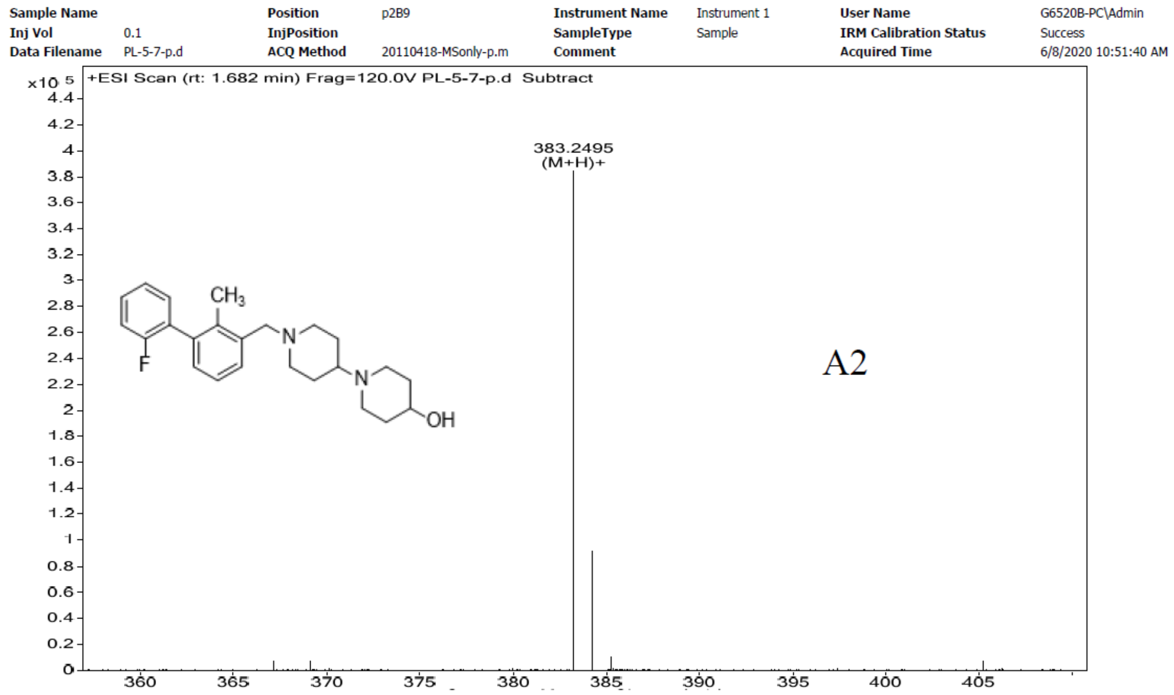

^{13}C NMR Spectra for Compound **A2**

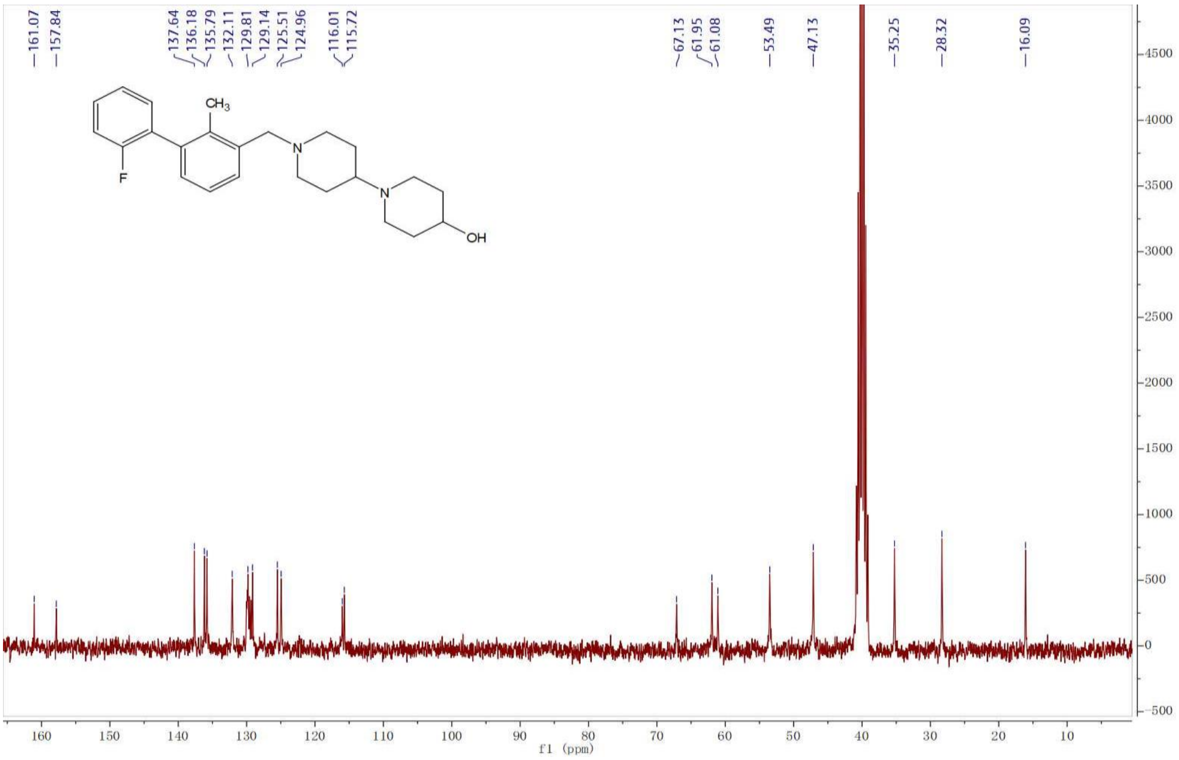

^{1}H NMR Spectra for Compound **A3**

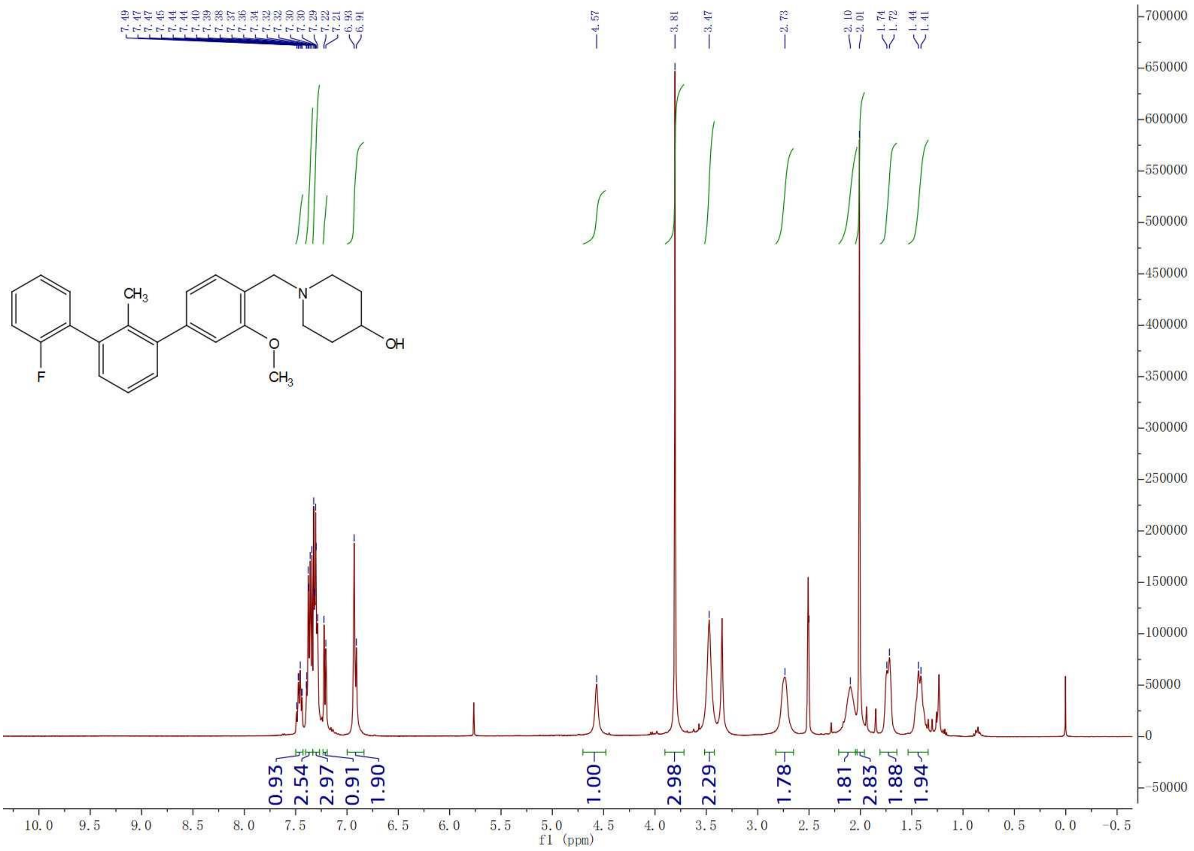

LCMS Spectra for Compound **A3**

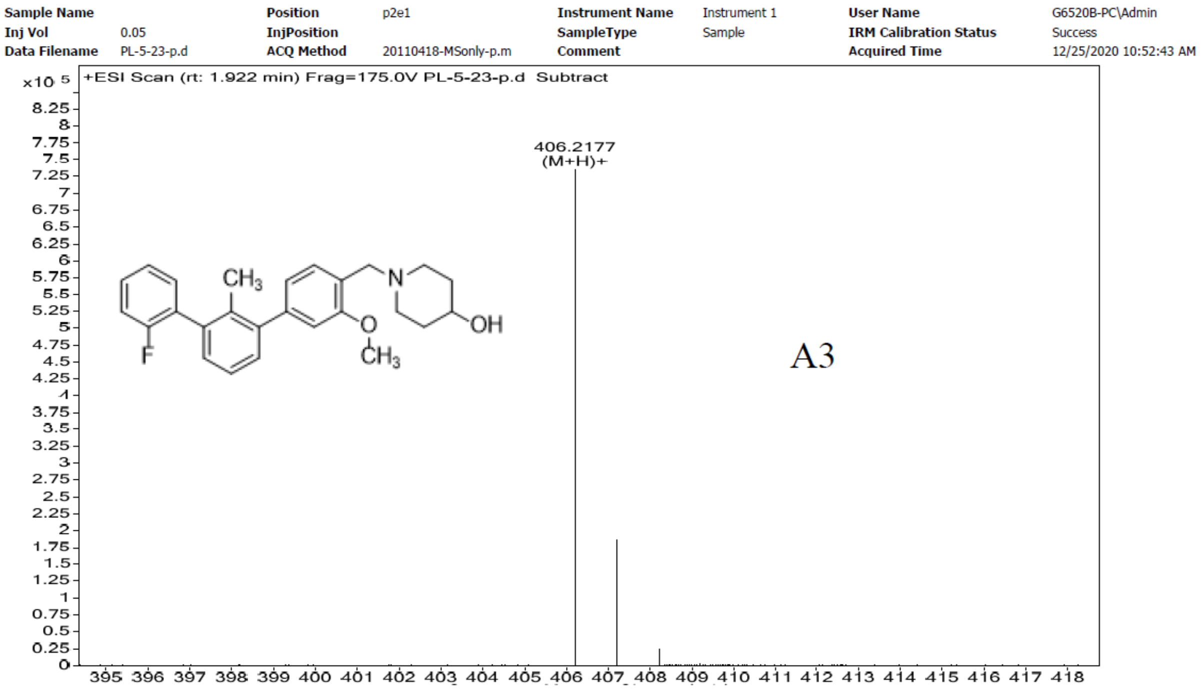

^{13}C NMR Spectra for Compound **A3**

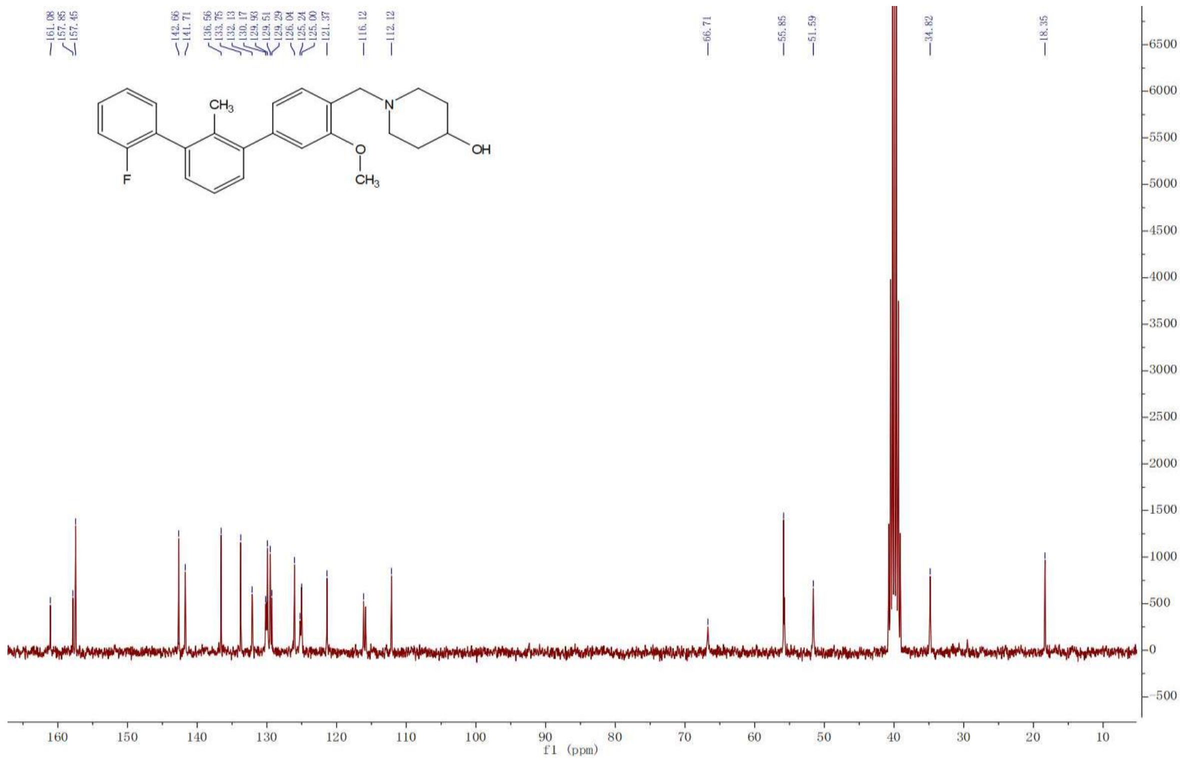

^{1}H NMR Spectra for Compound **A4**

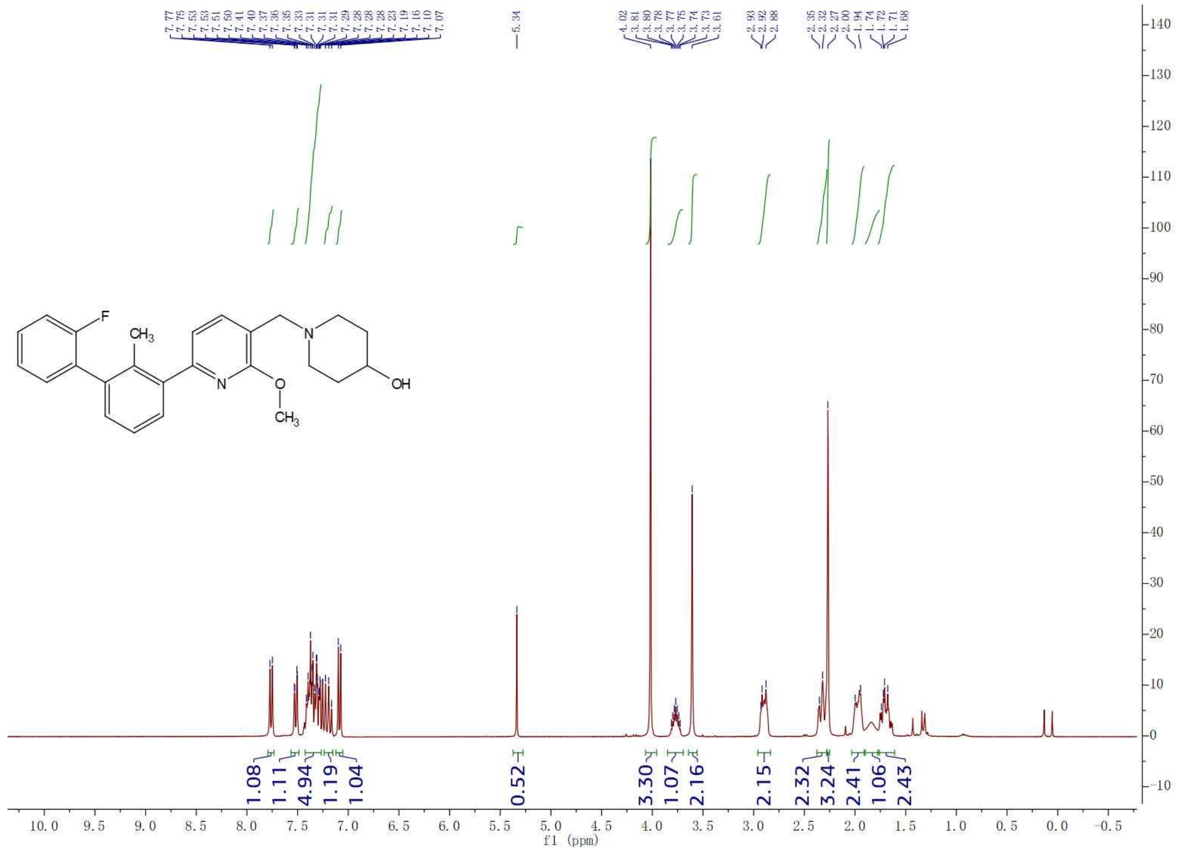

LCMS Spectra for Compound **A4**

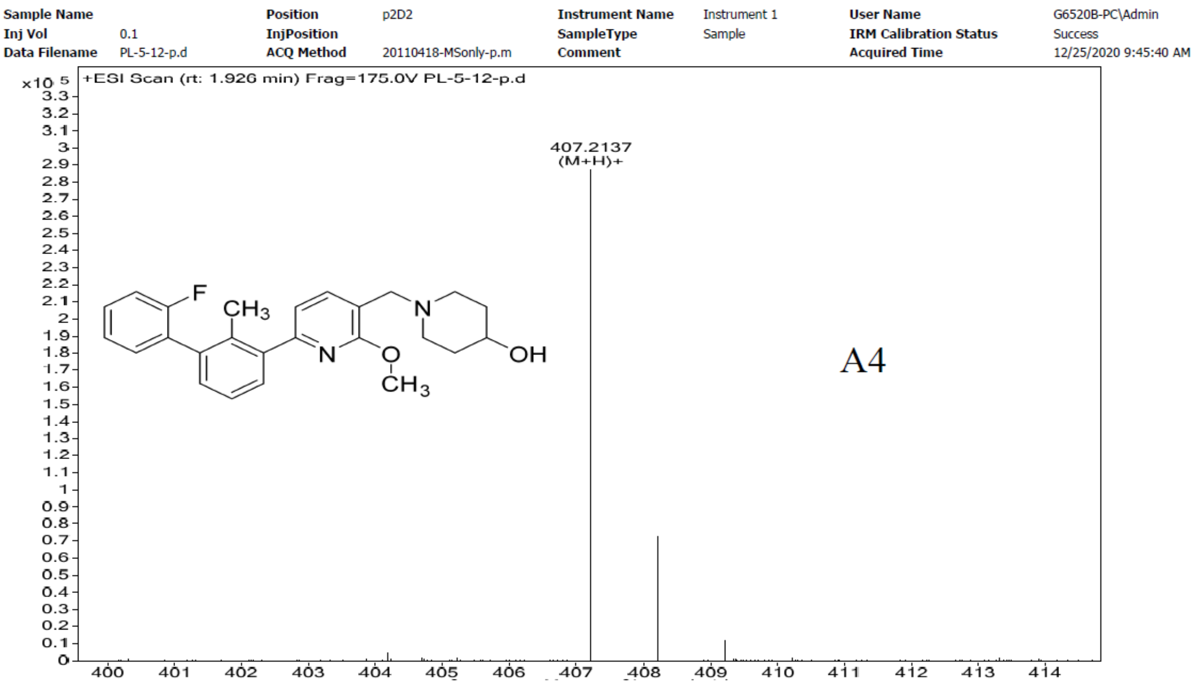

^{13}C NMR Spectra for Compound **A4**

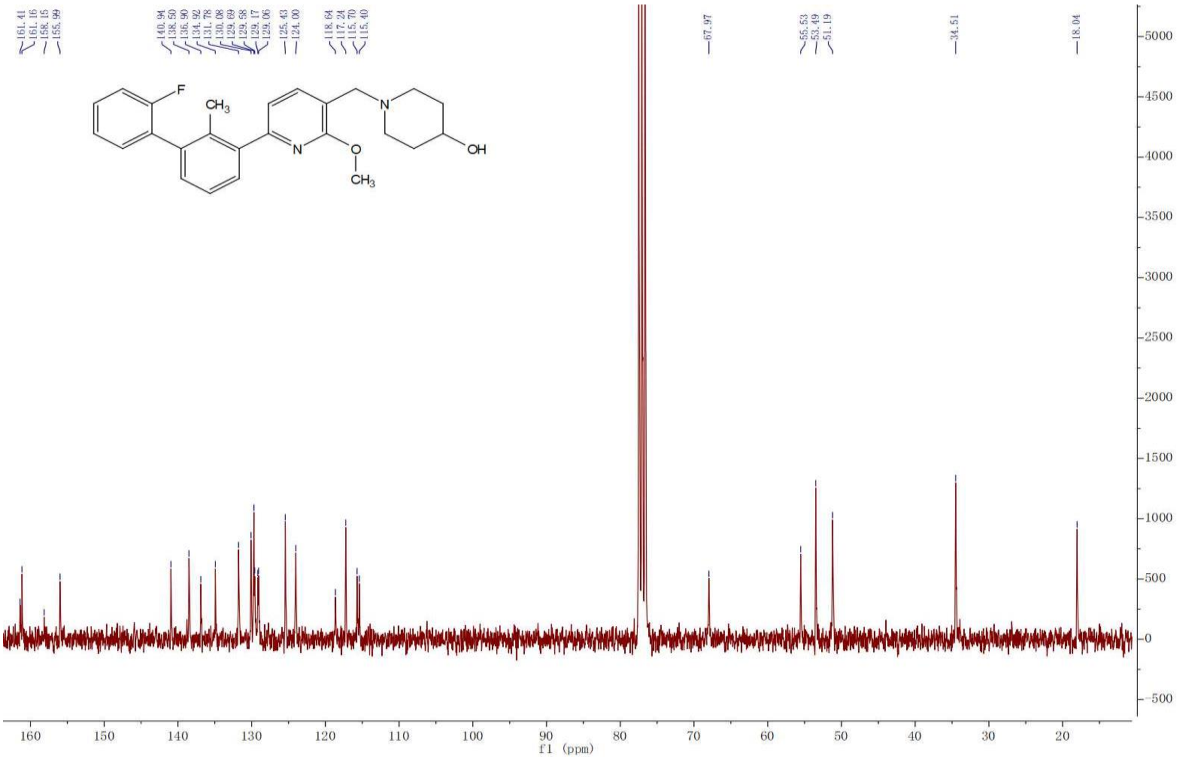

^{1}H NMR Spectra for Compound **A5**

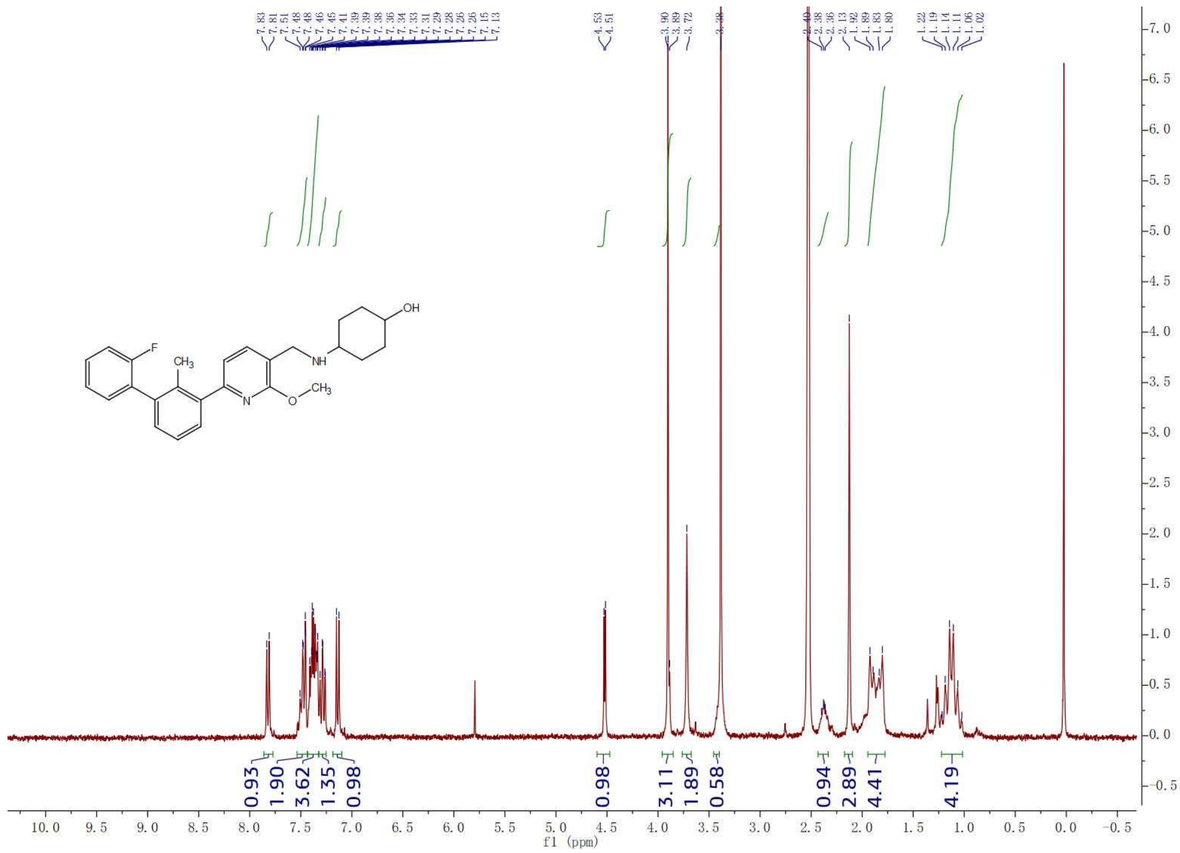

LCMS Spectra for Compound **A5**

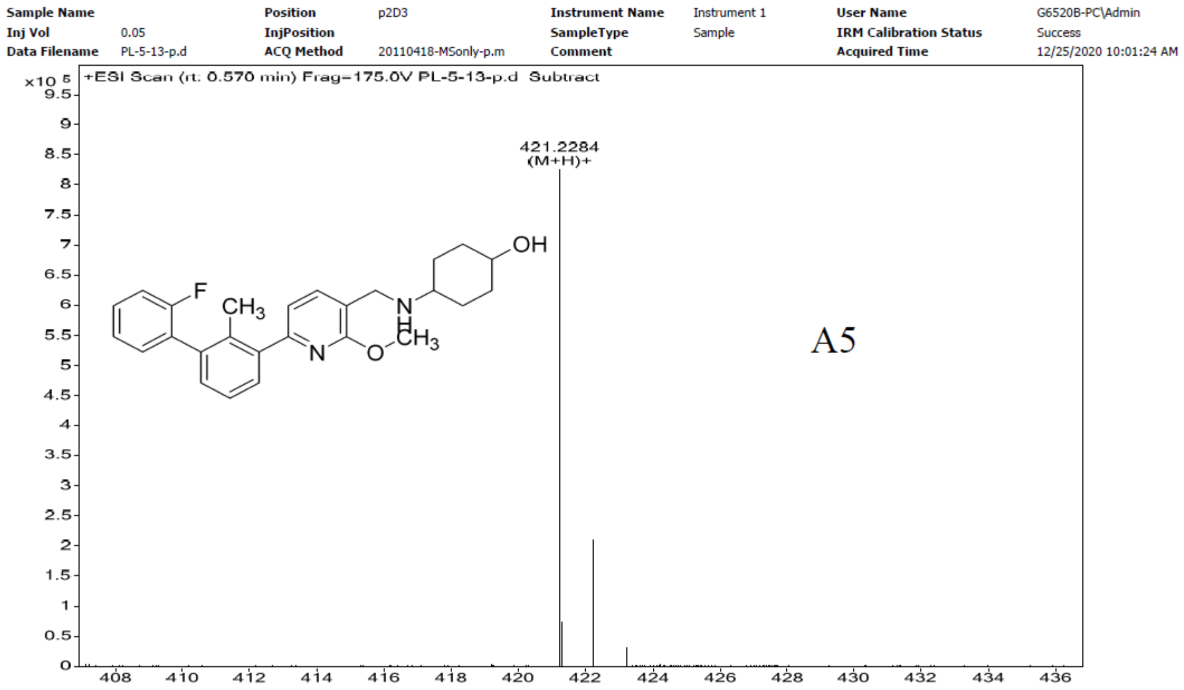

^{13}C NMR Spectra for Compound **A5**

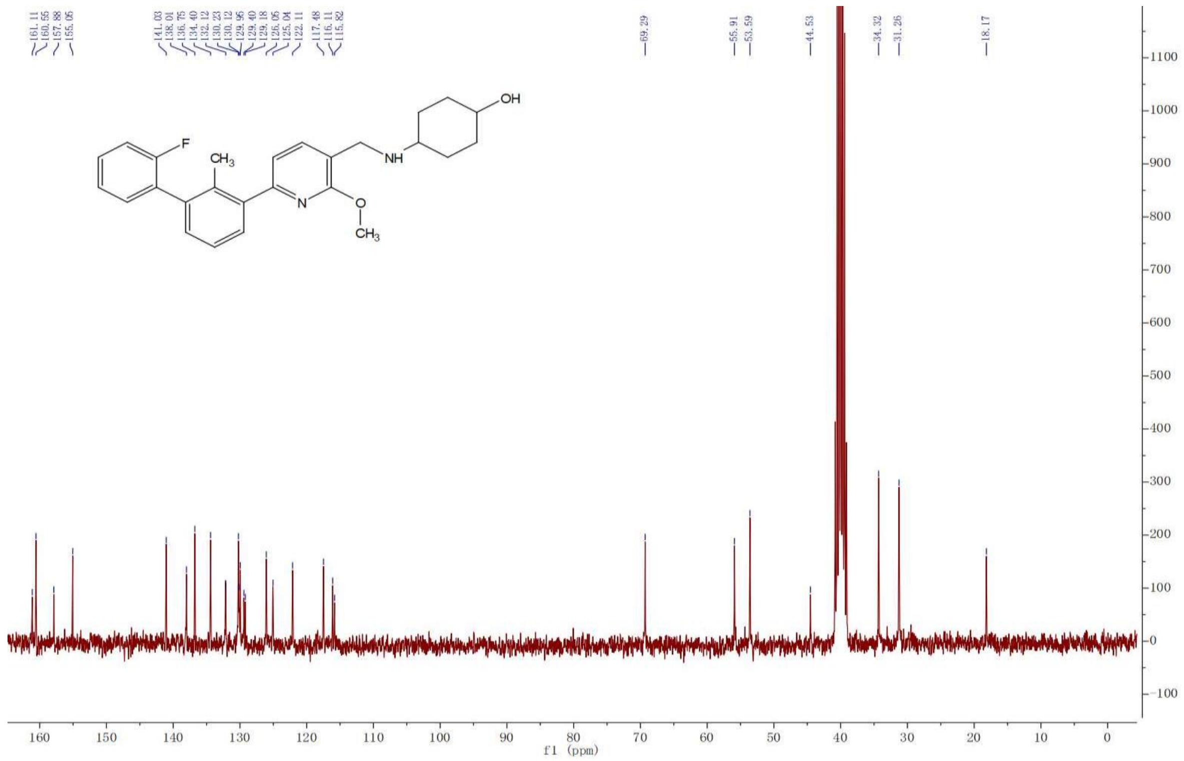

^{1}H NMR Spectra for Compound **A6**

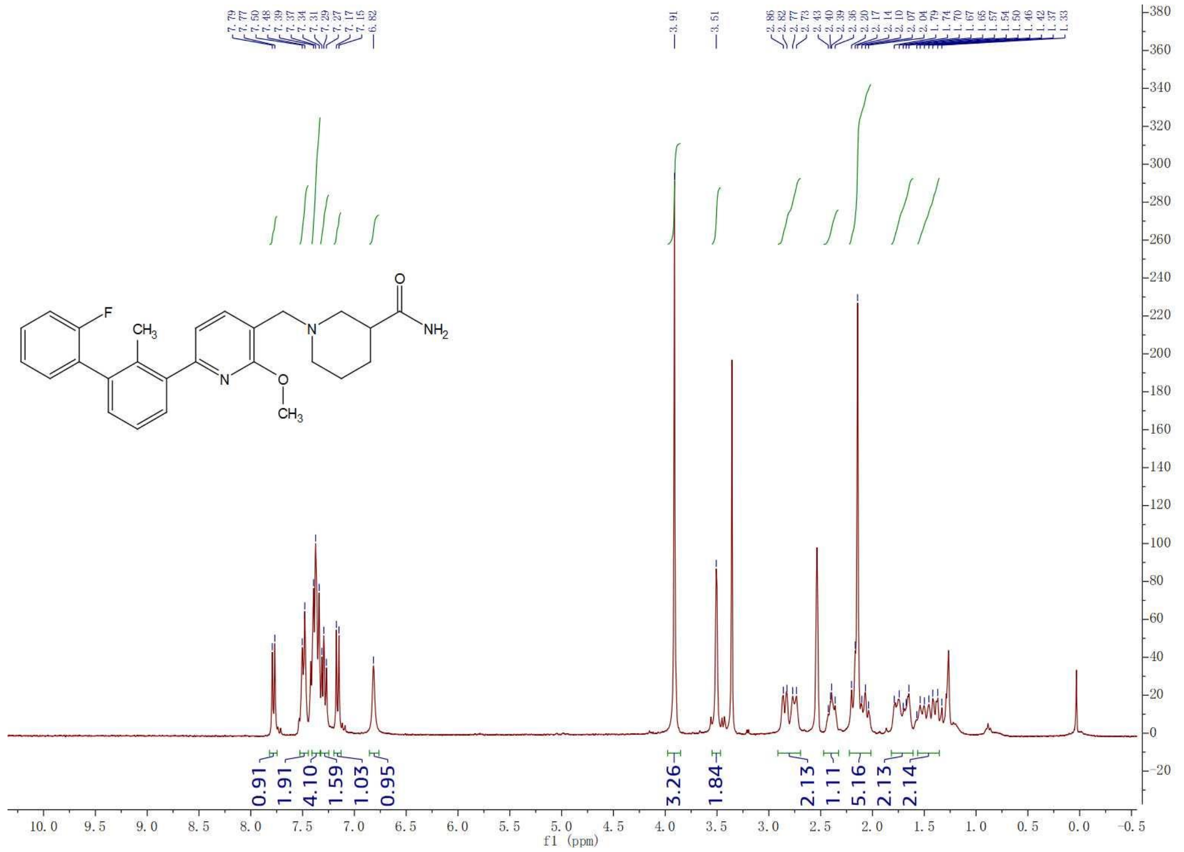

LCMS Spectra for Compound **A6**

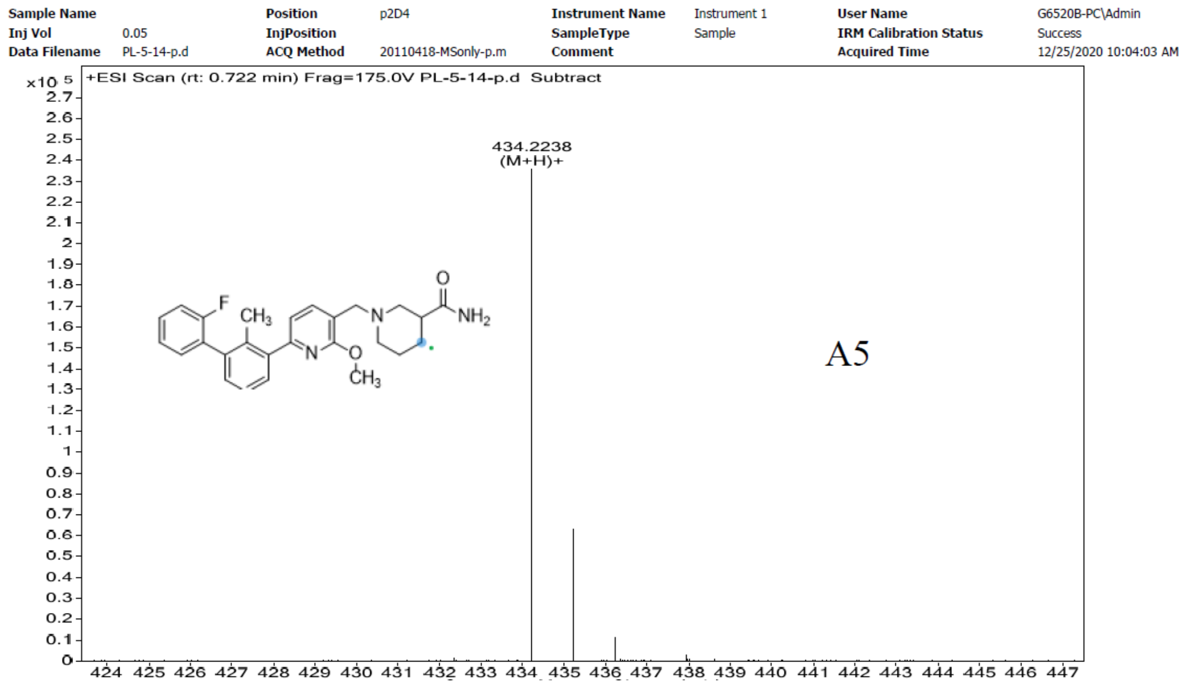

^{13}C NMR Spectra for Compound **A6**

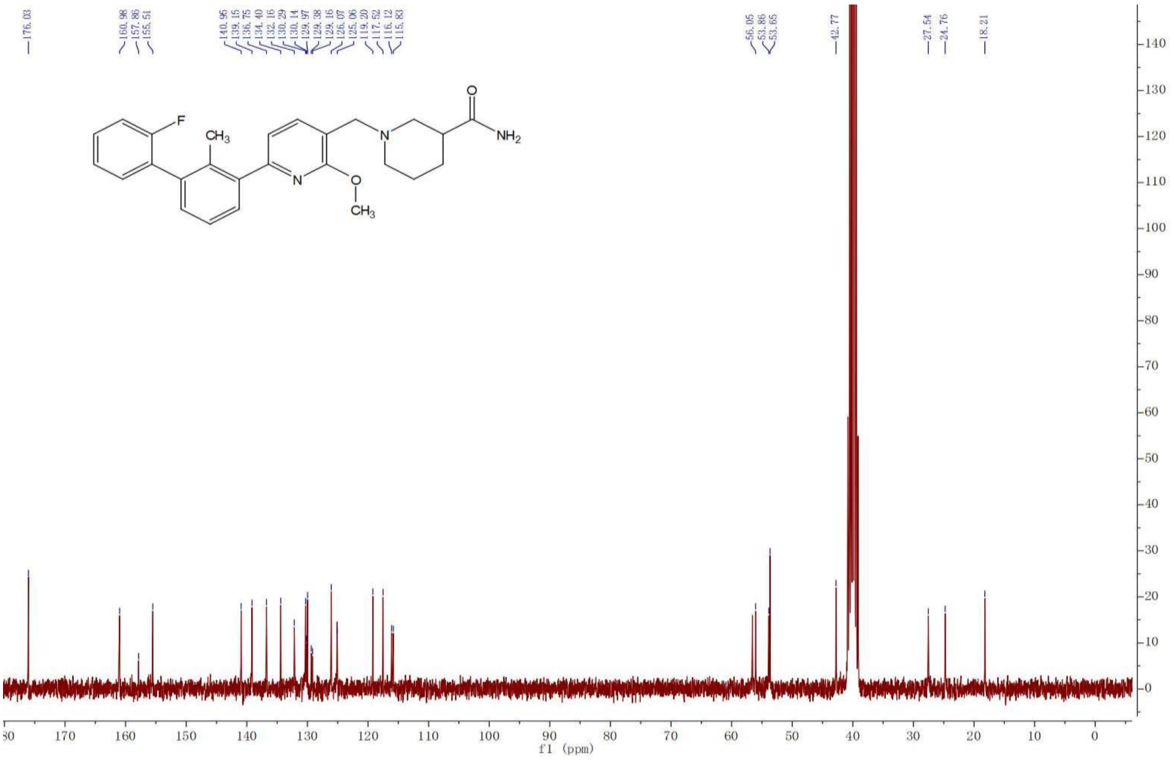

^{1}H NMR Spectra for Compound **A7**

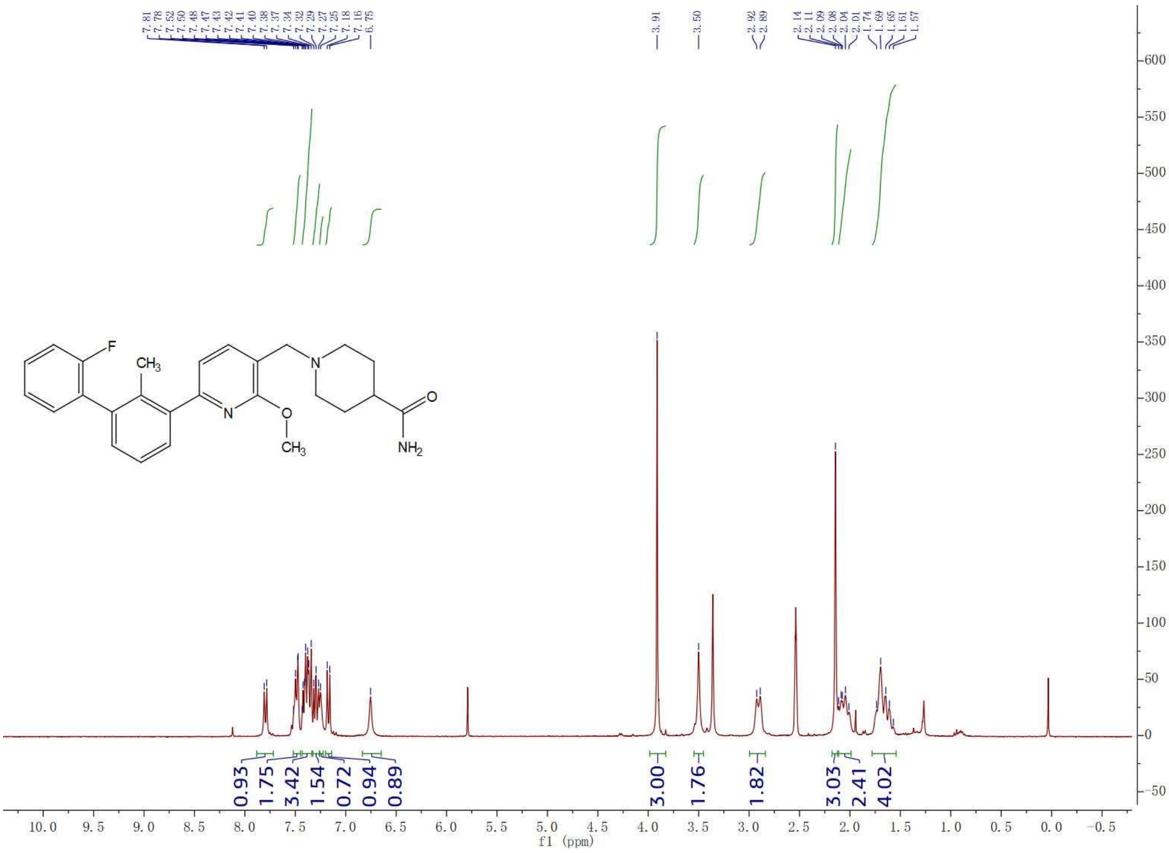

LCMS Spectra for Compound **A7**

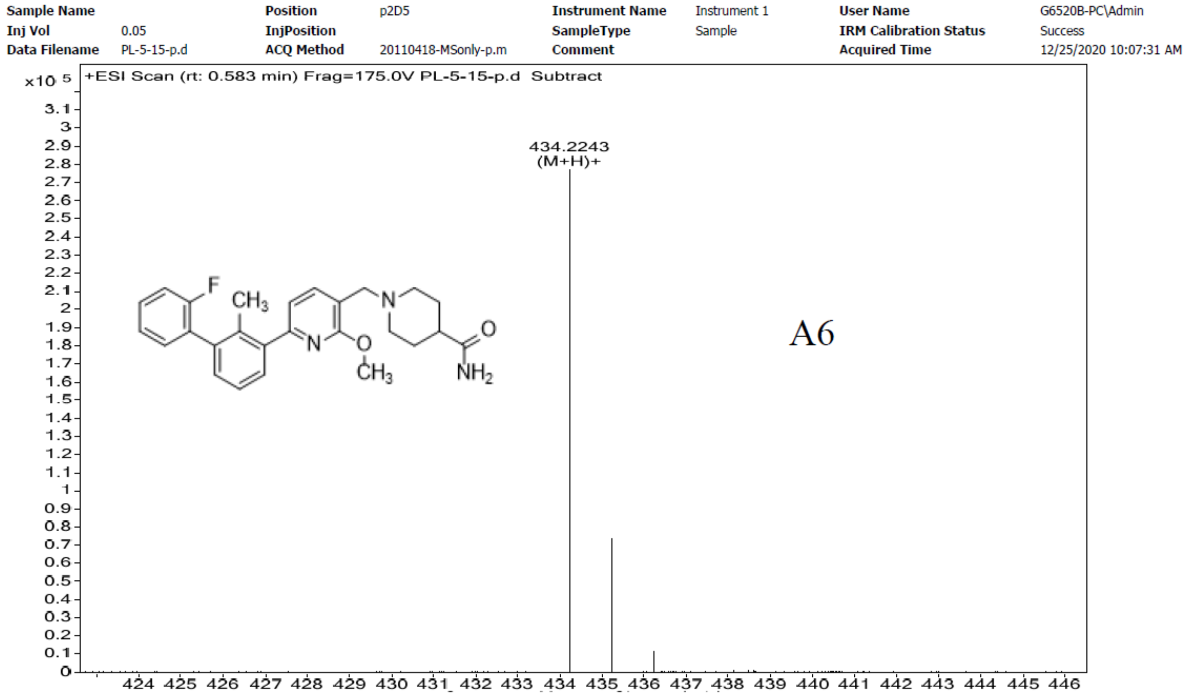

^{13}C NMR Spectra for Compound **A7**

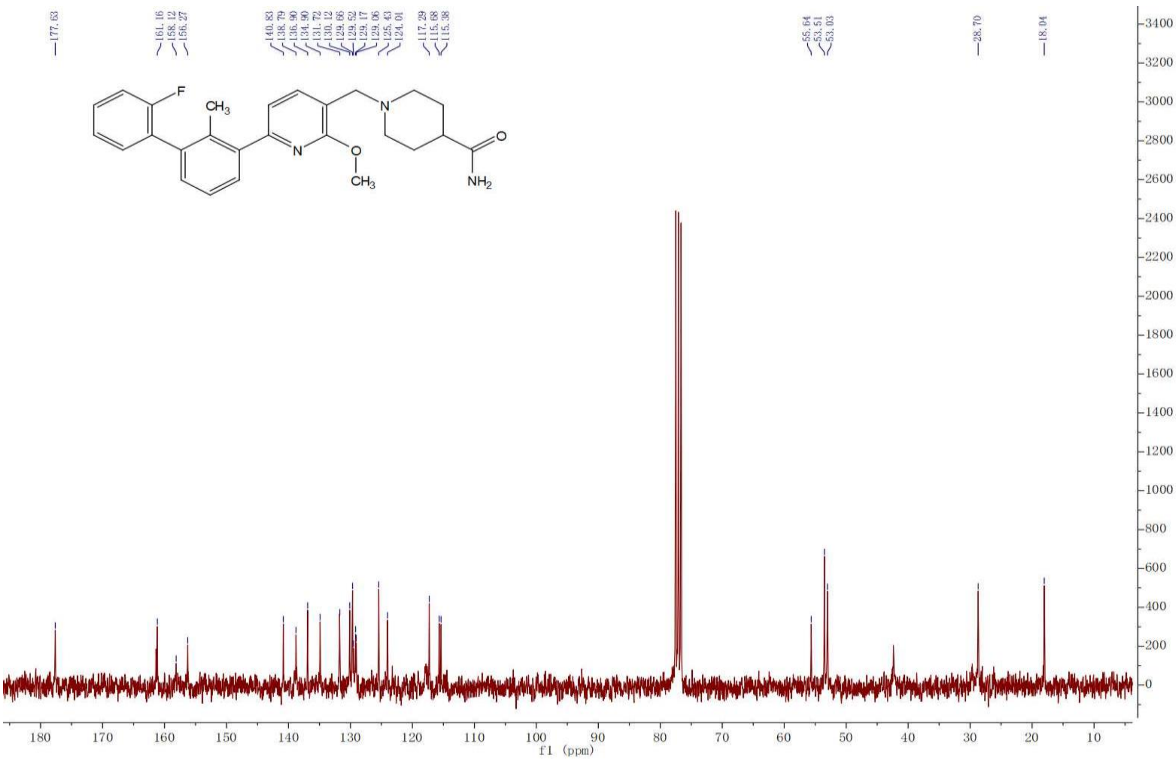

^{1}H NMR Spectra for Compound **A8**

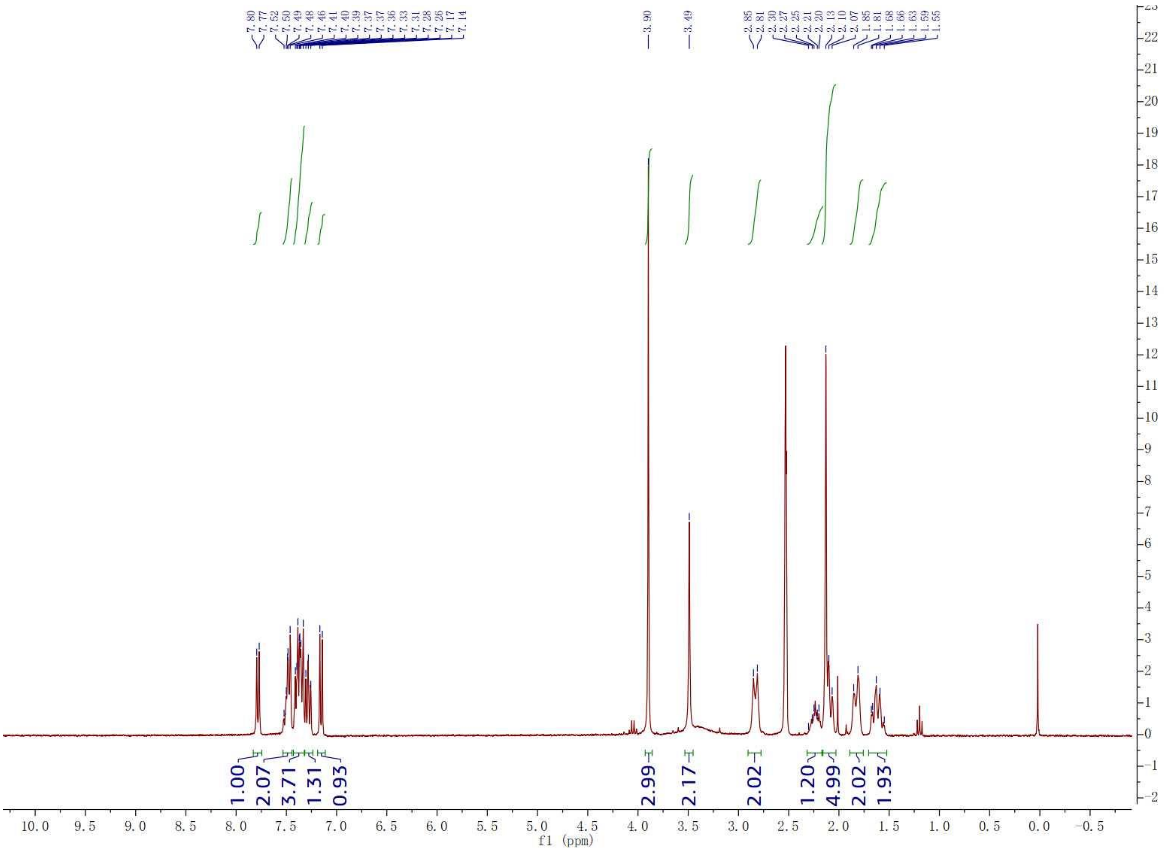

LCMS Spectra for Compound **A8**

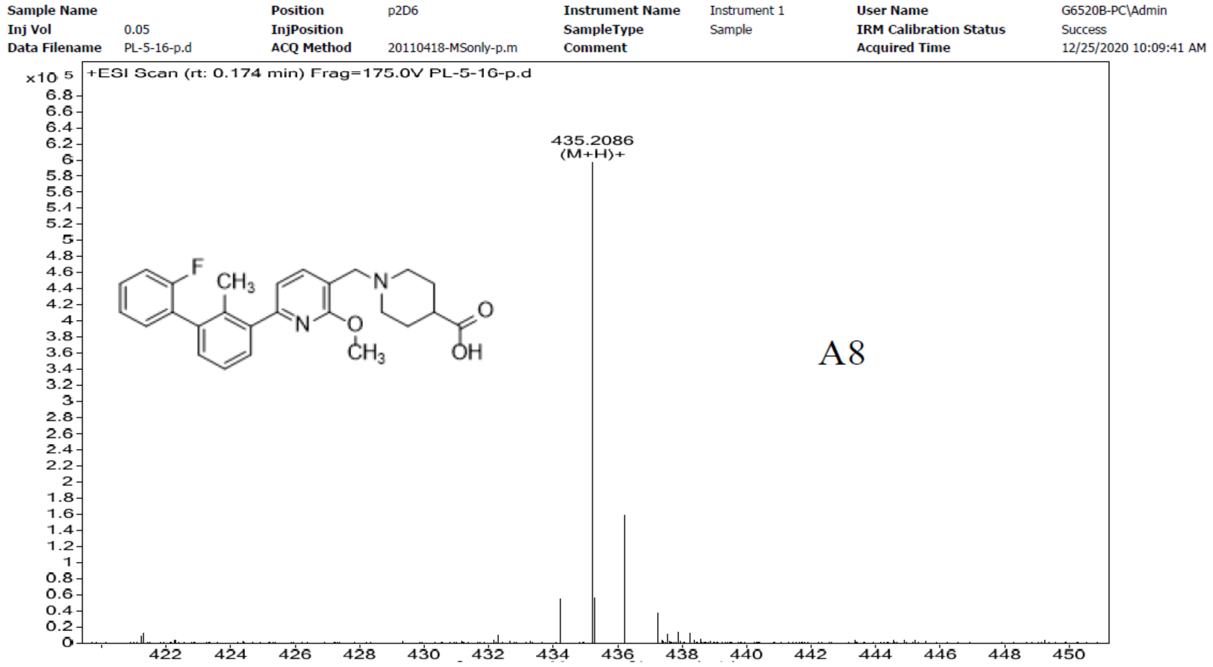

^{13}C NMR Spectra for Compound **A8**

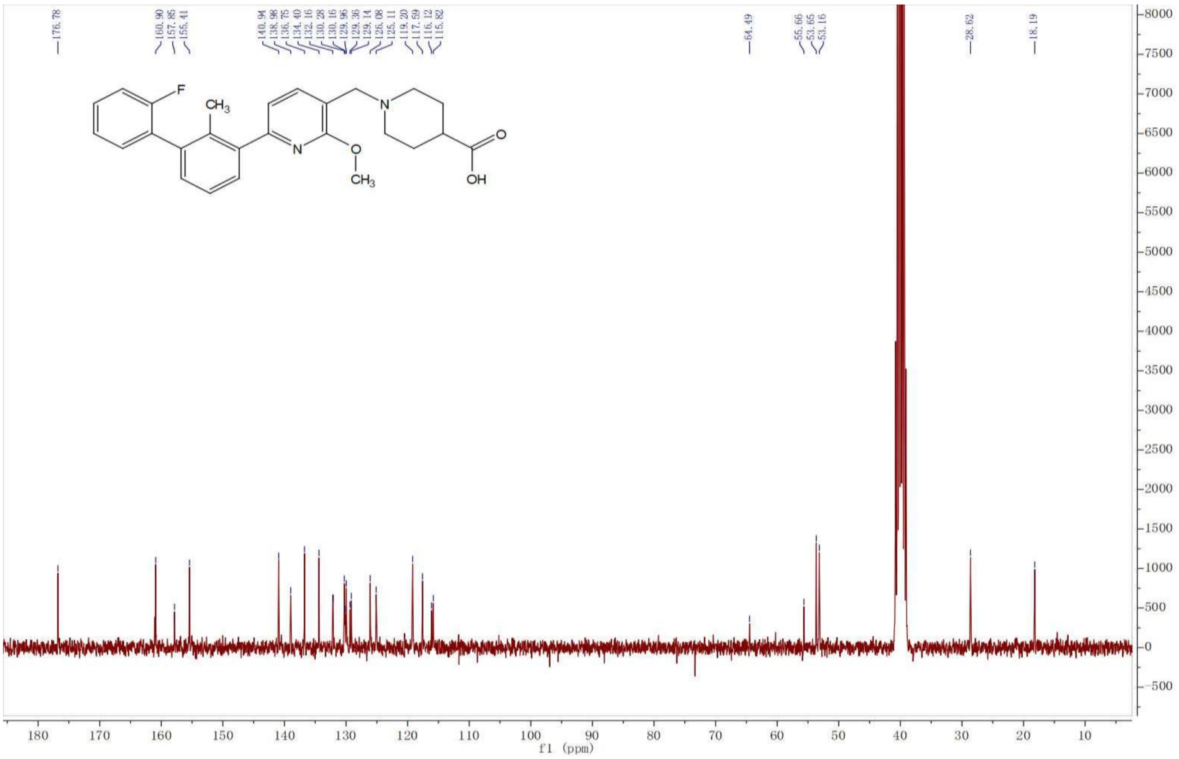

^{1}H NMR Spectra for Compound **A9**

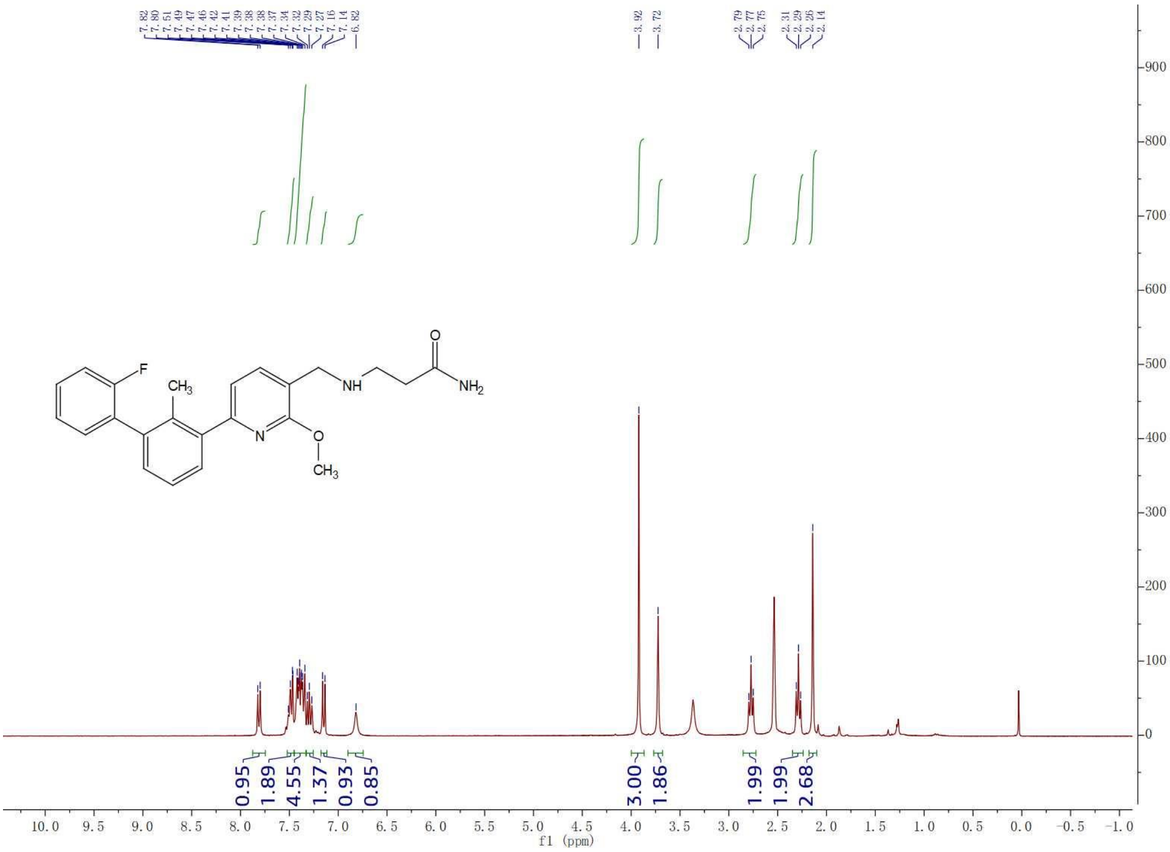

LCMS Spectra for Compound **A9**

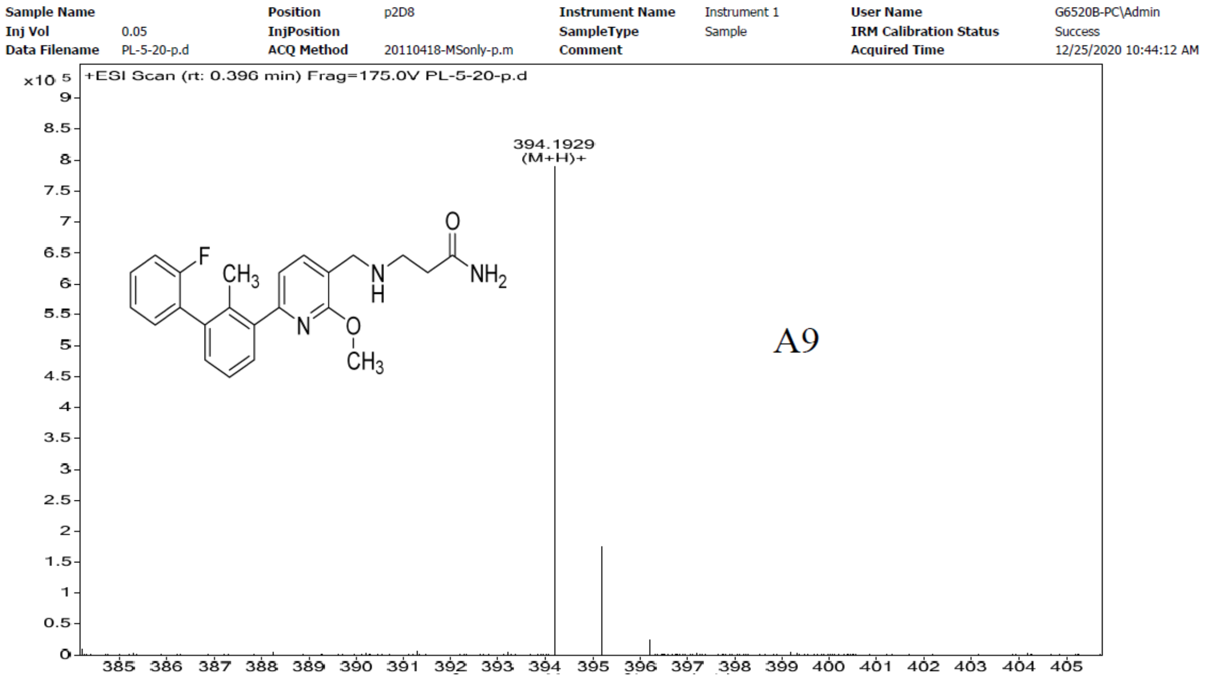

^{13}C NMR Spectra for Compound **A9**

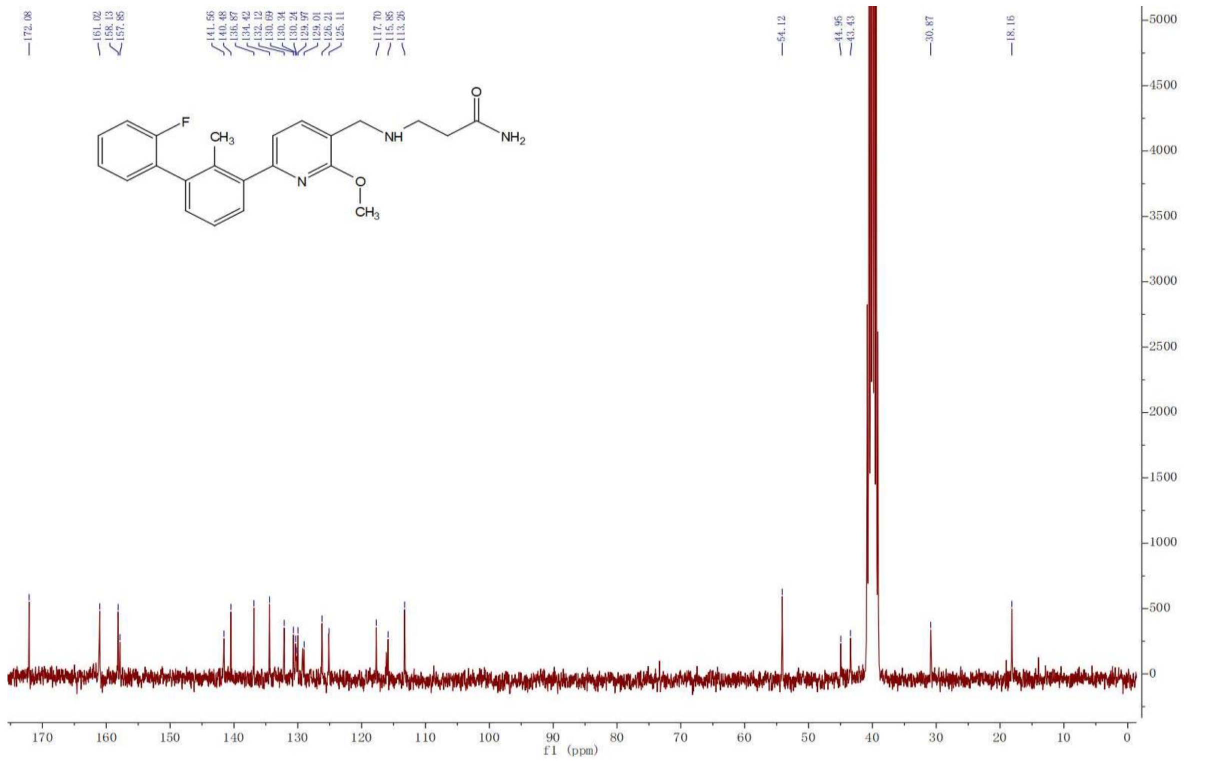

^{1}H NMR Spectra for Compound **A10**

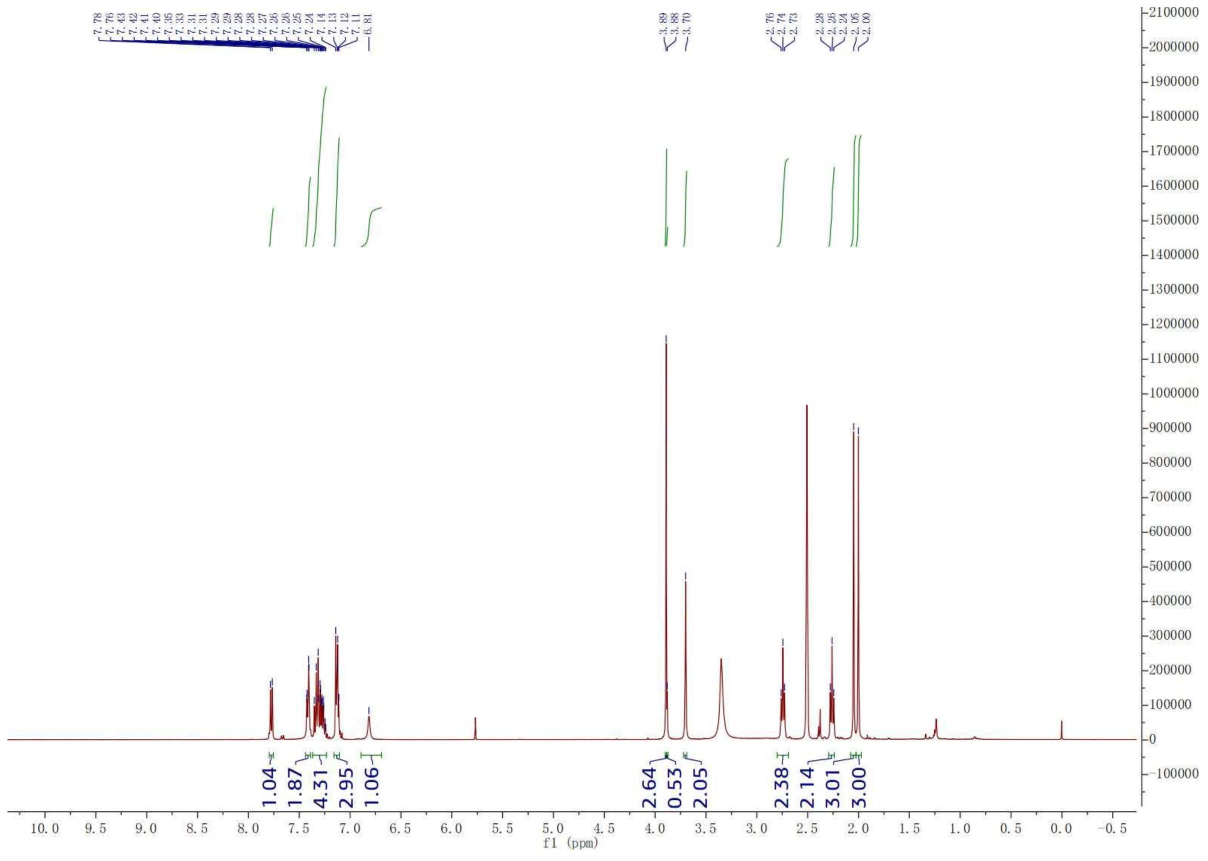

LCMS Spectra for Compound **A10**

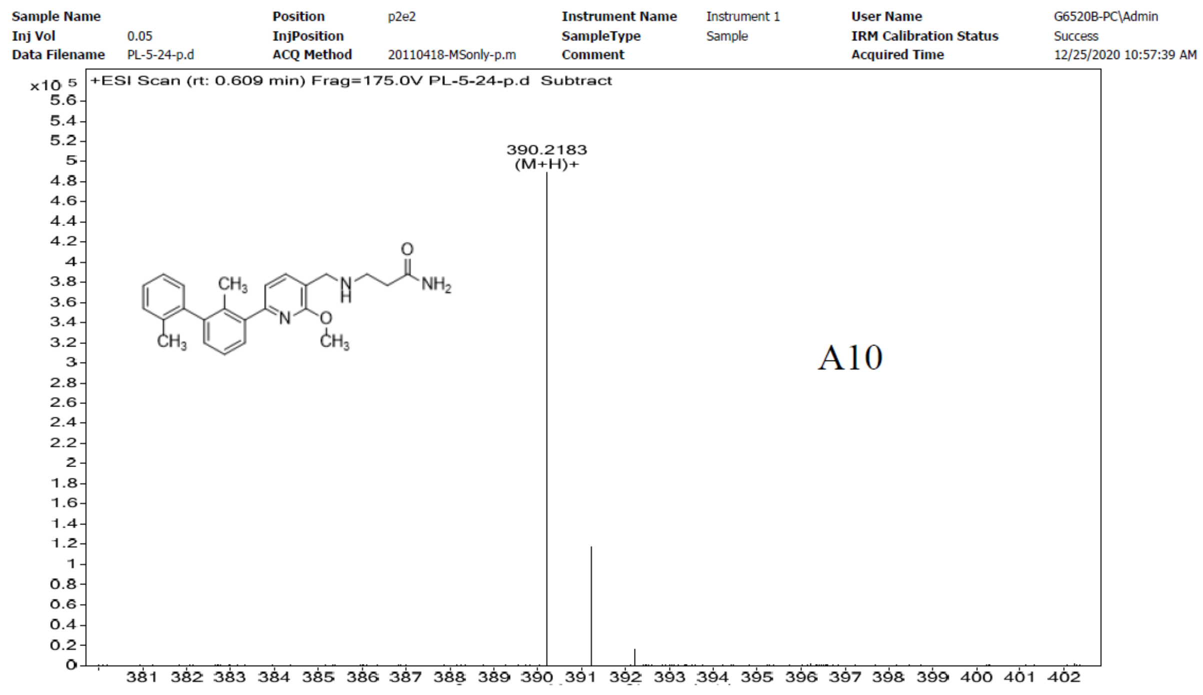

^{13}C NMR Spectra for Compound **A10**

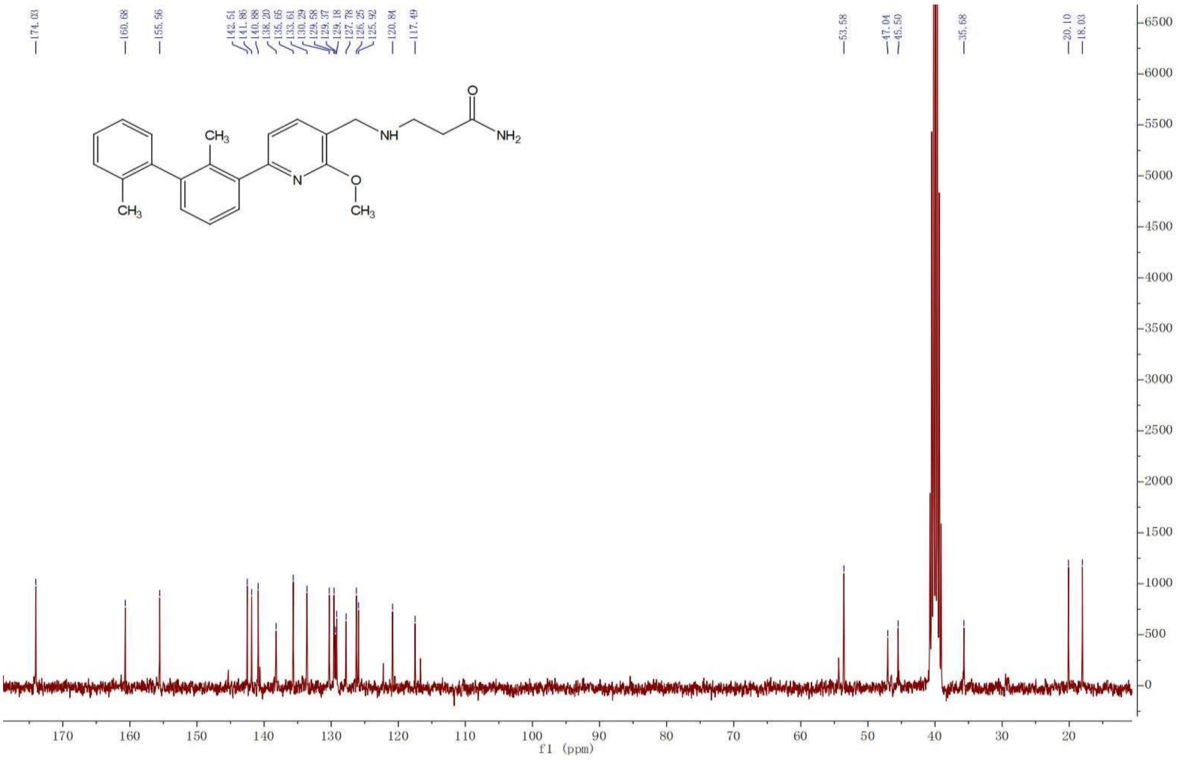

^{1}H NMR Spectra for Compound **A11**

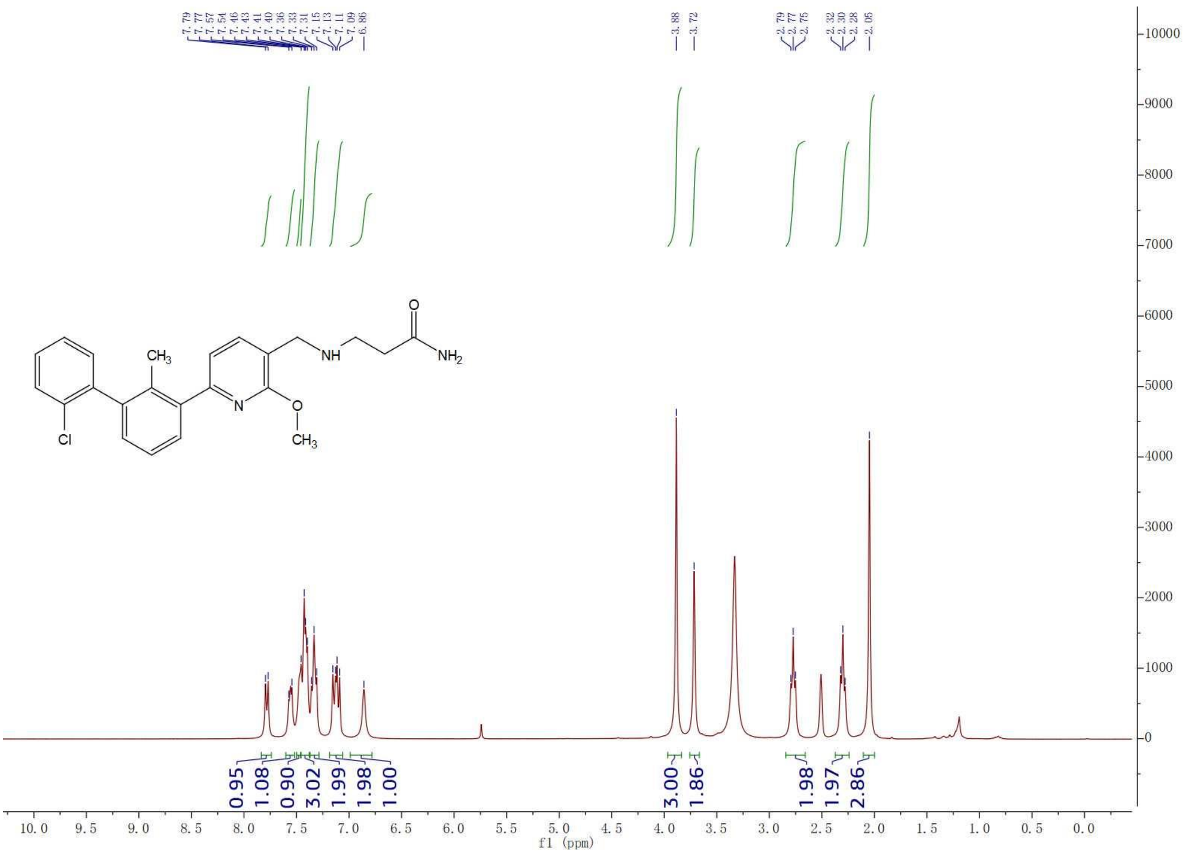

LCMS Spectra for Compound **A11**

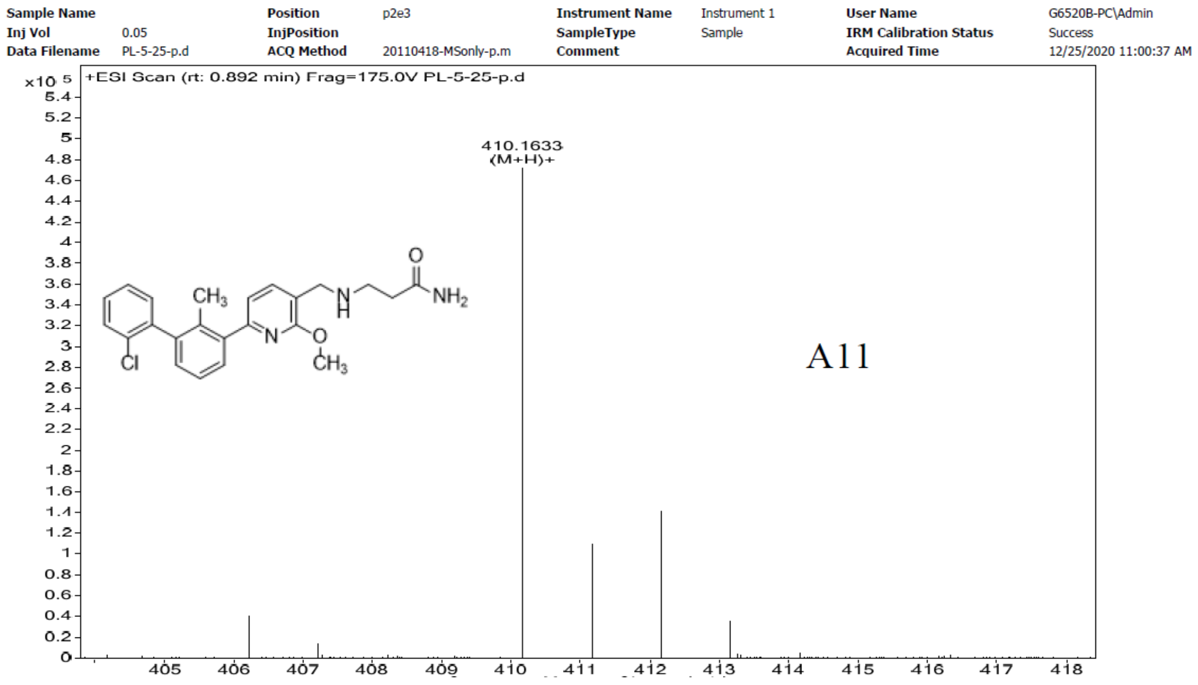

^{13}C NMR Spectra for Compound **A11**

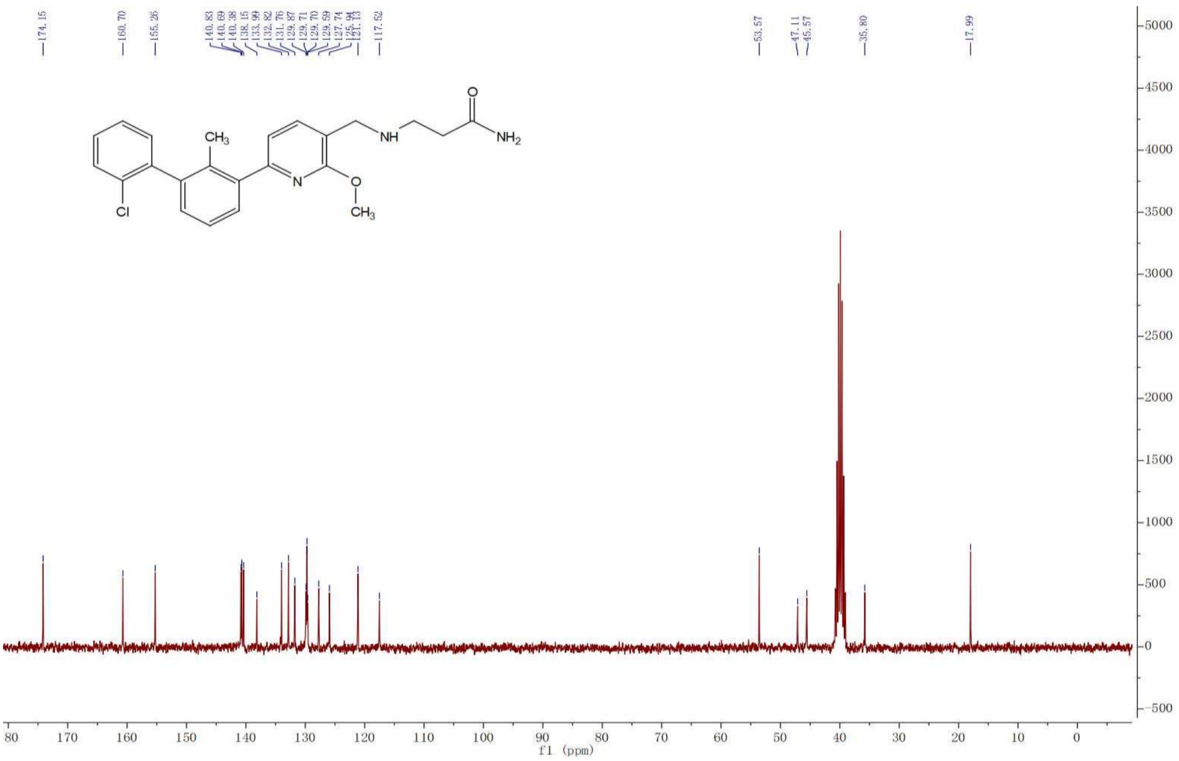

^{1}H NMR Spectra for Compound **A12**

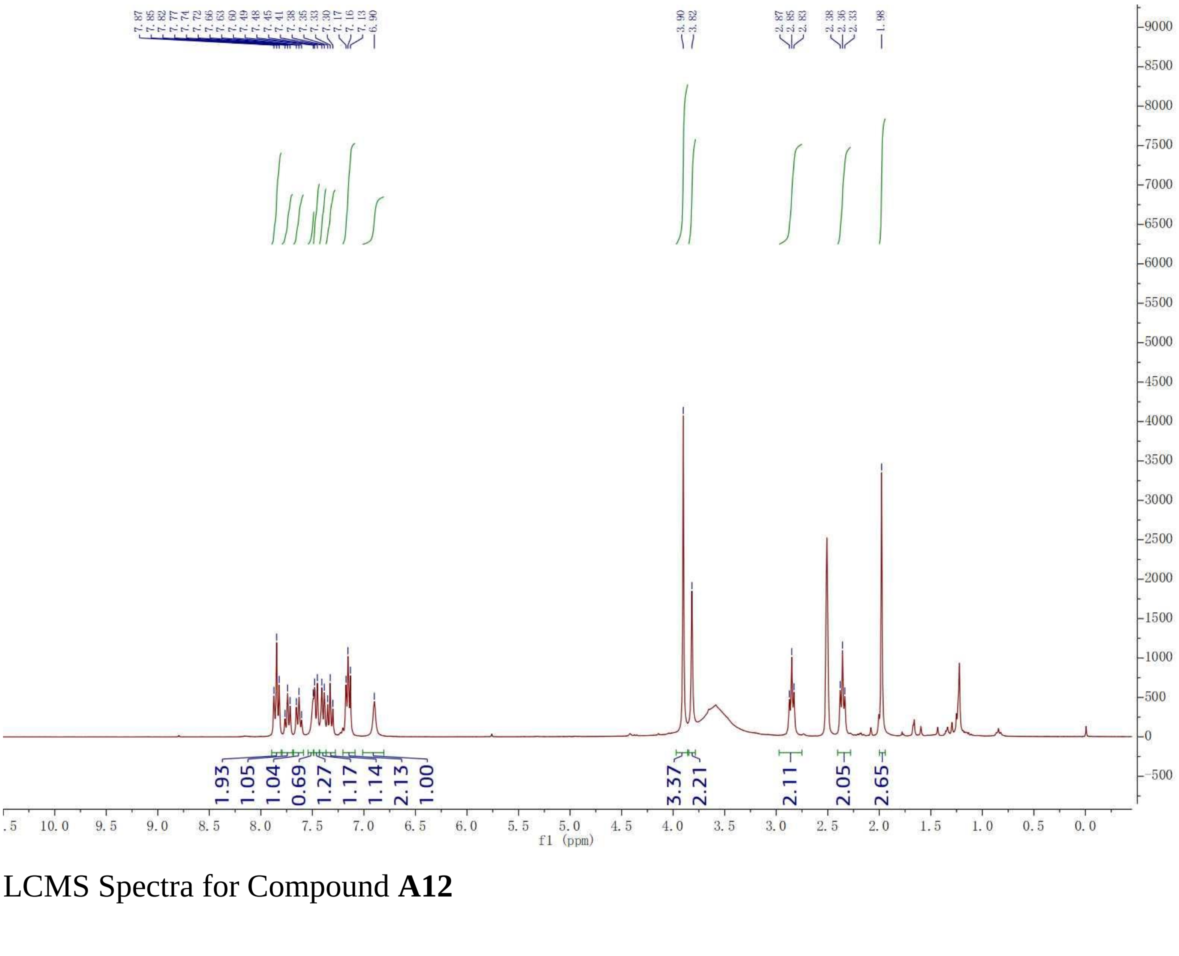

LCMS Spectra for Compound **A12**

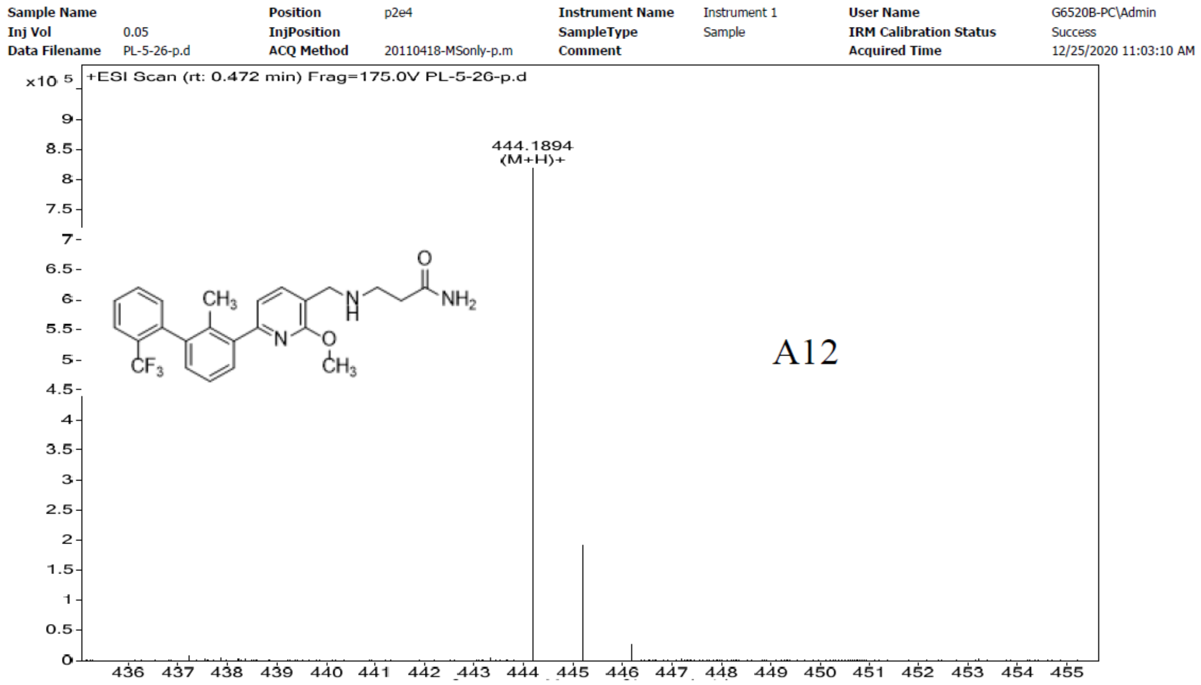

^{13}C NMR Spectra for Compound **A12**

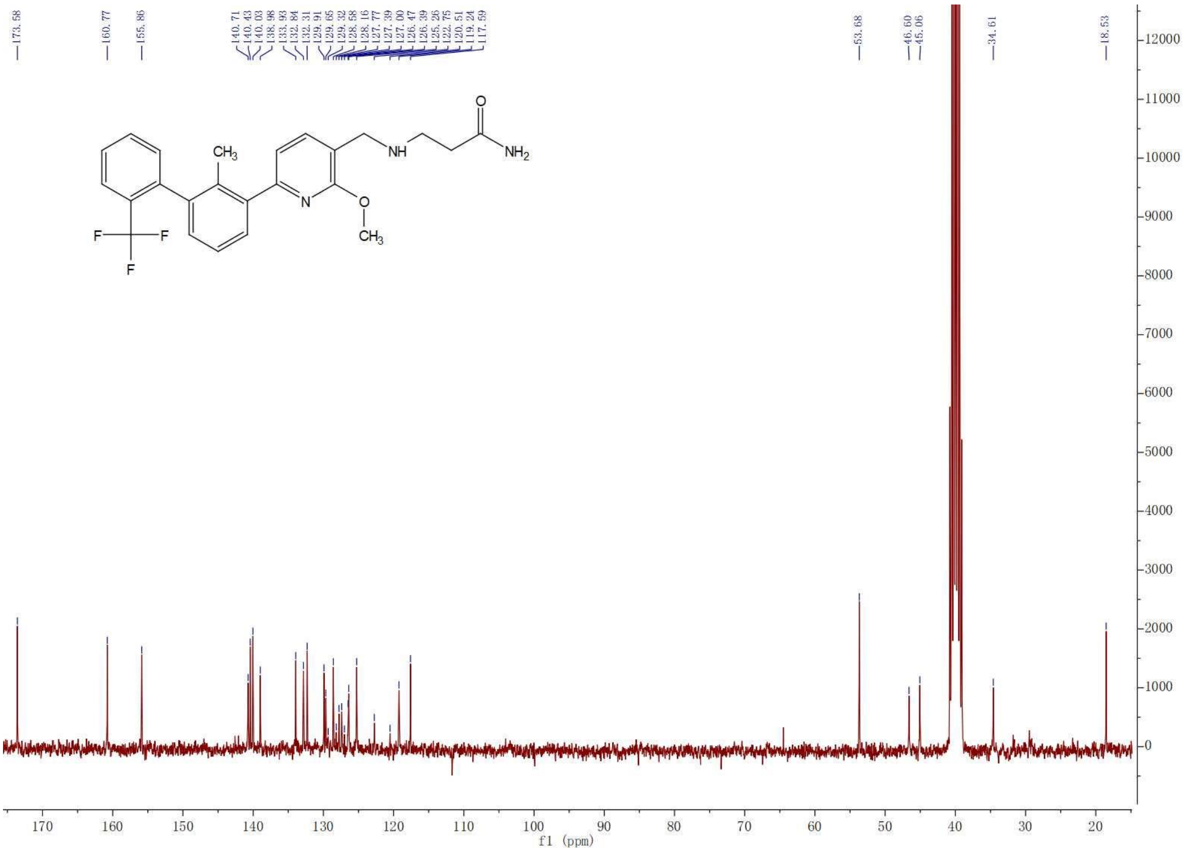

^{1}H NMR Spectra for Compound **A13**

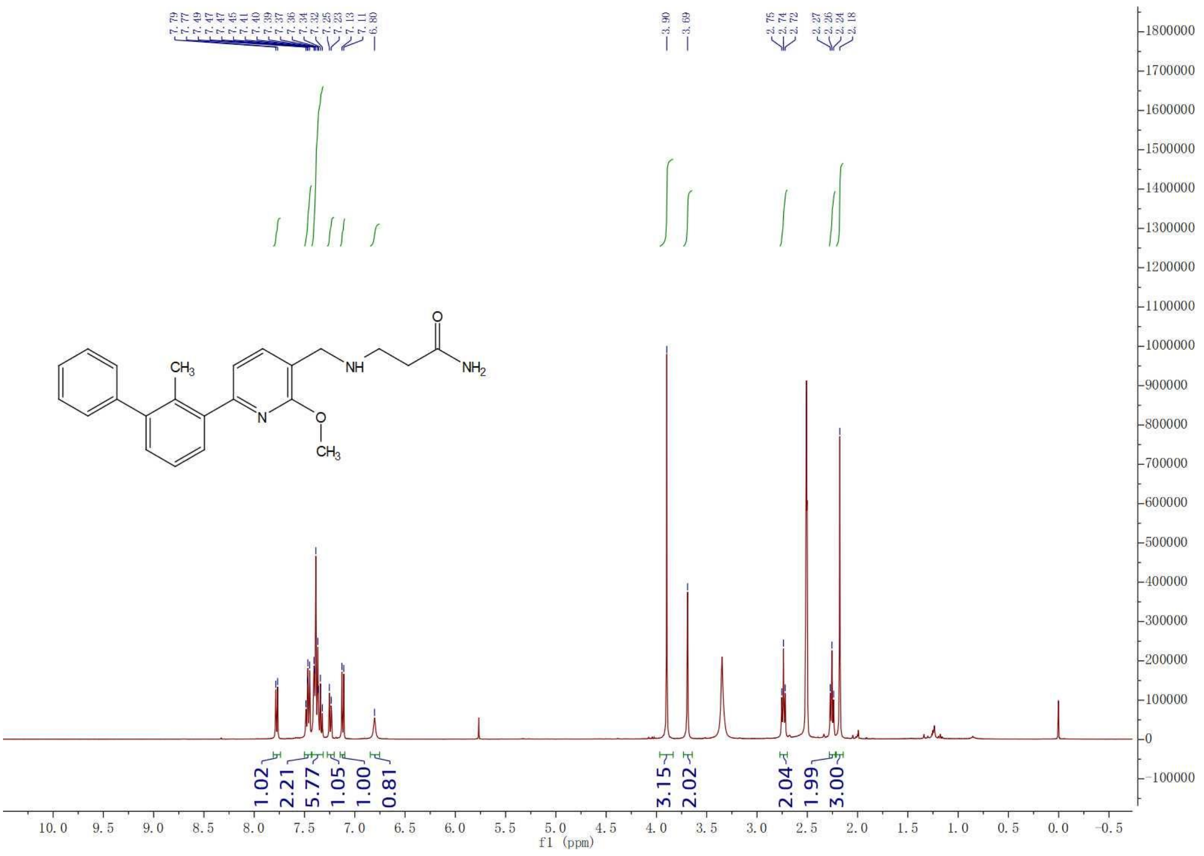

LCMS Spectra for Compound **A13**

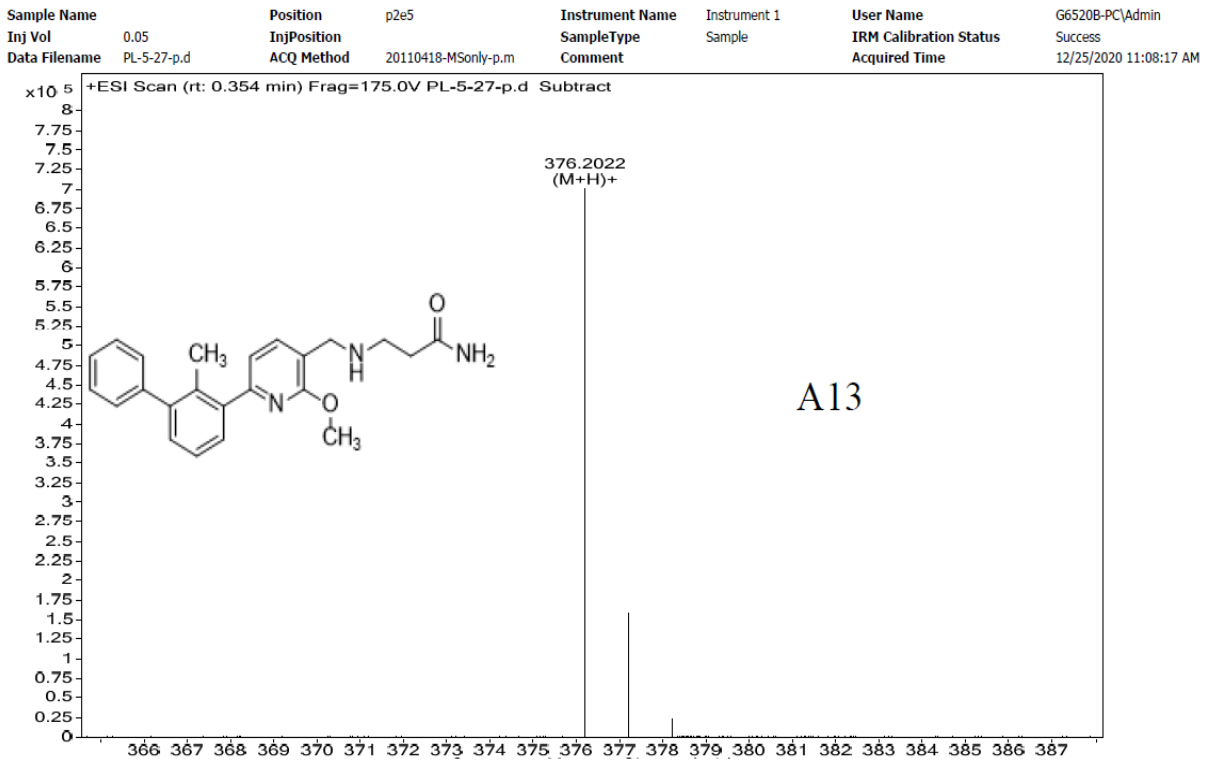

^{13}C NMR Spectra for Compound **A13**

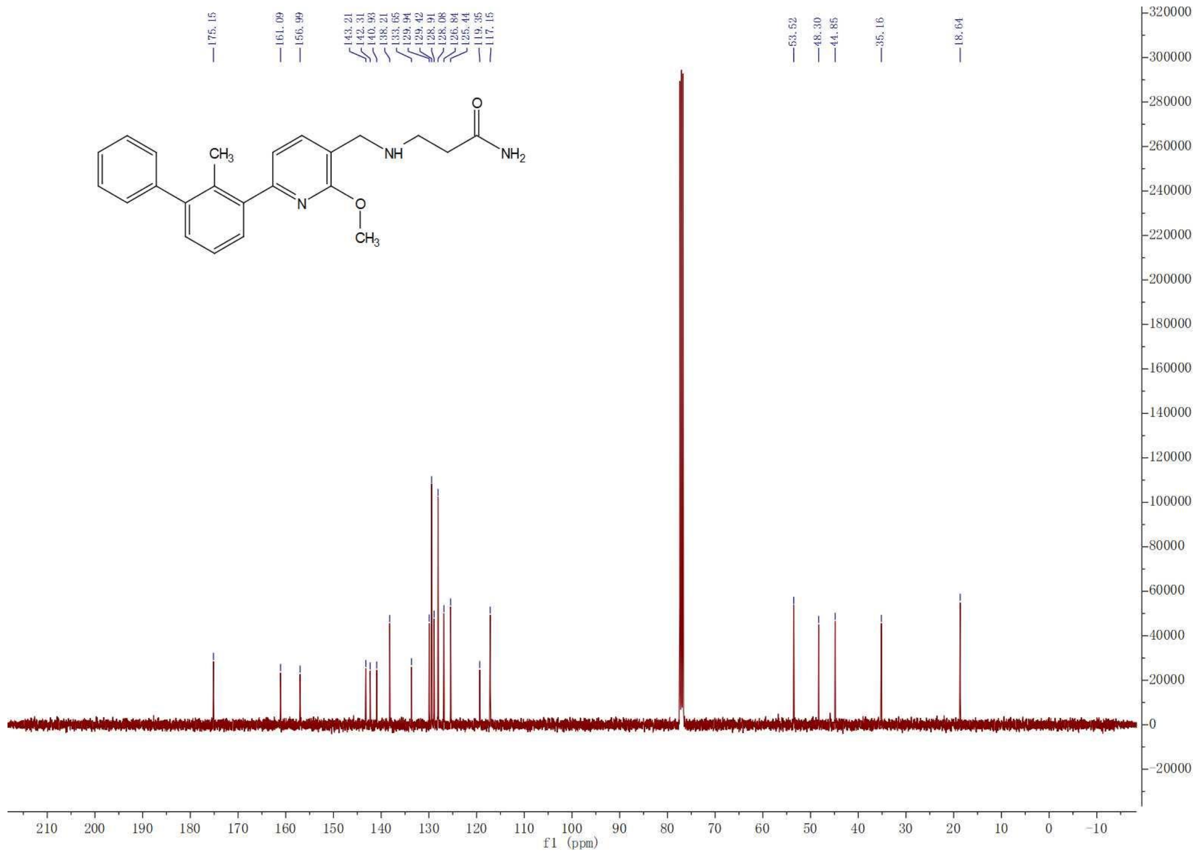

^{1}H NMR Spectra for Compound **A14**

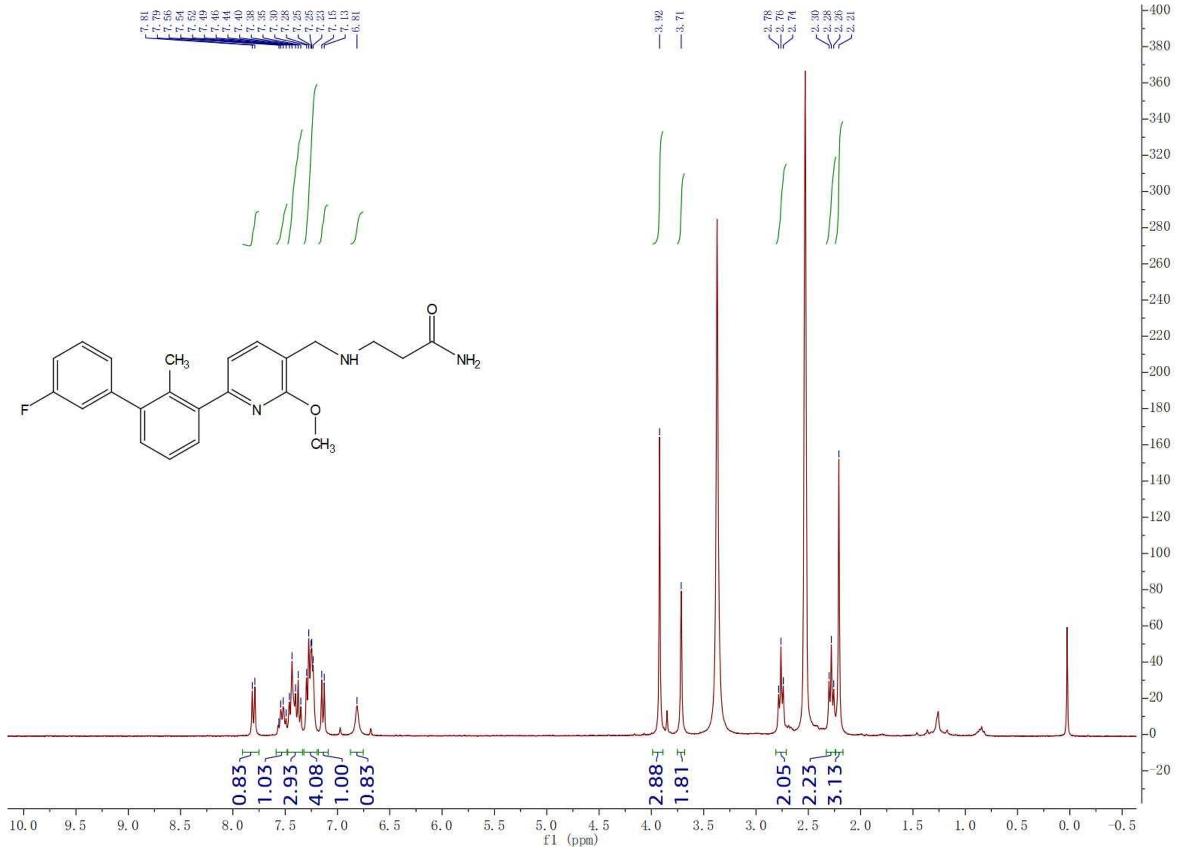

LCMS Spectra for Compound **A14**

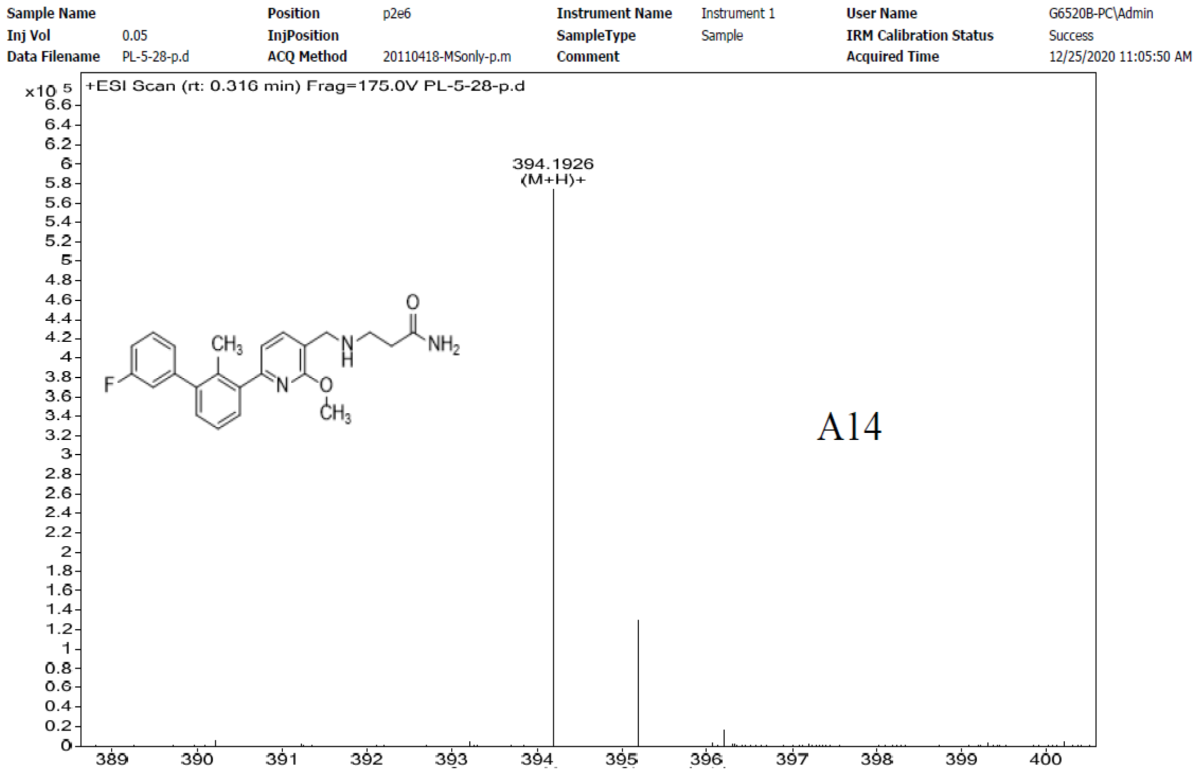

^{13}C NMR Spectra for Compound **A14**

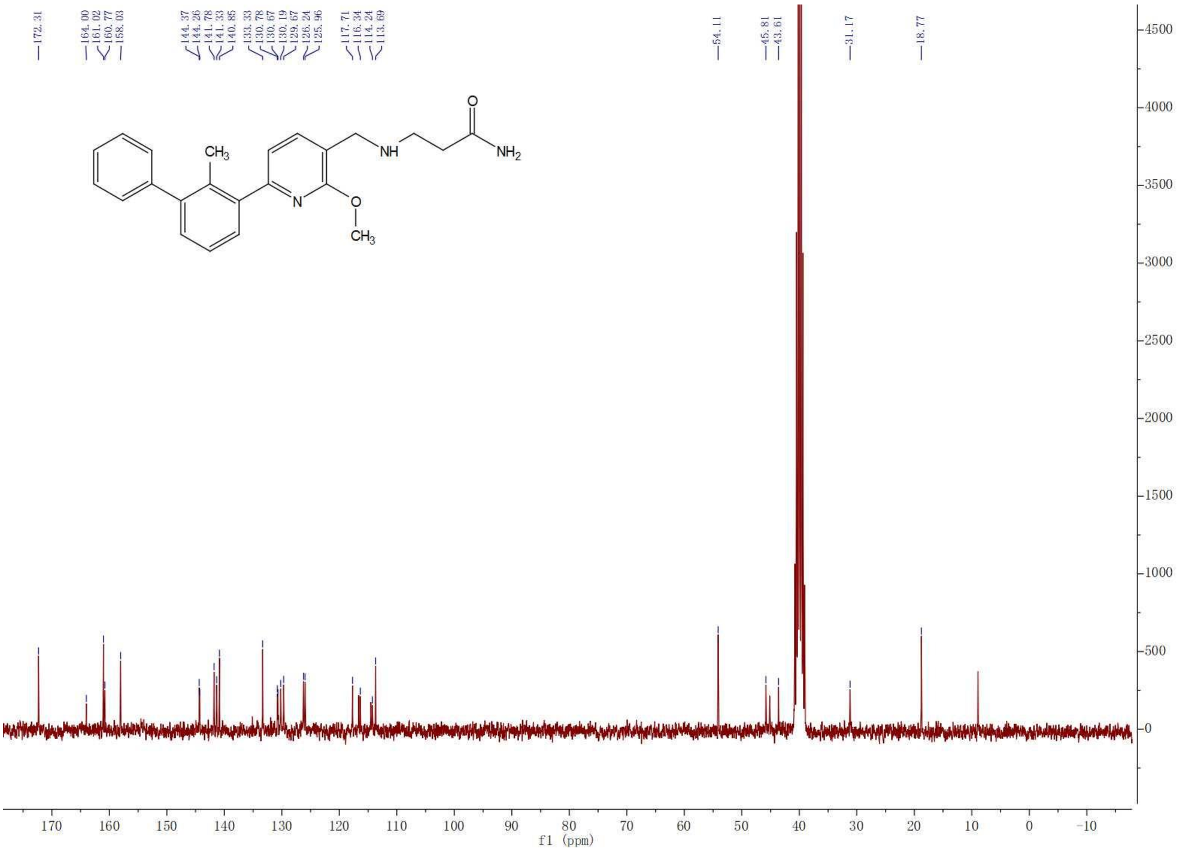

^{1}H NMR Spectra for Compound **A15**

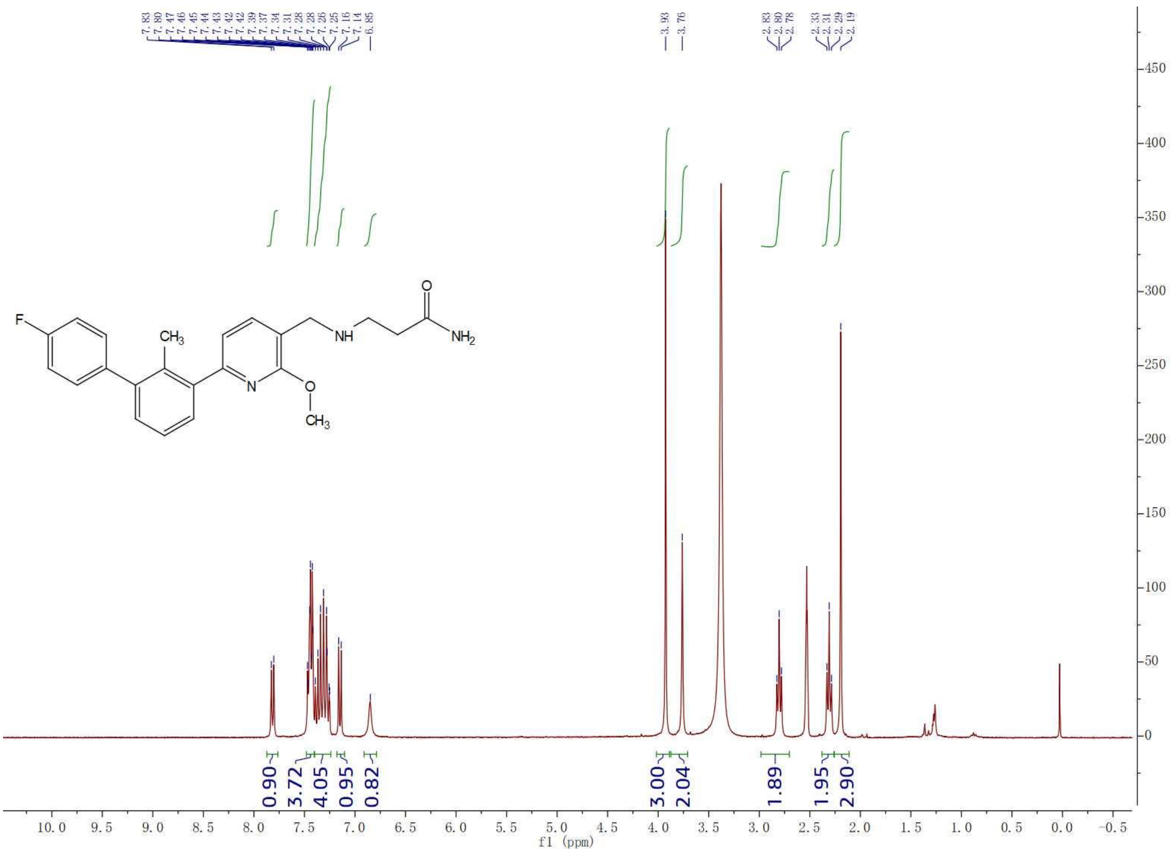

LCMS Spectra for Compound **A15**

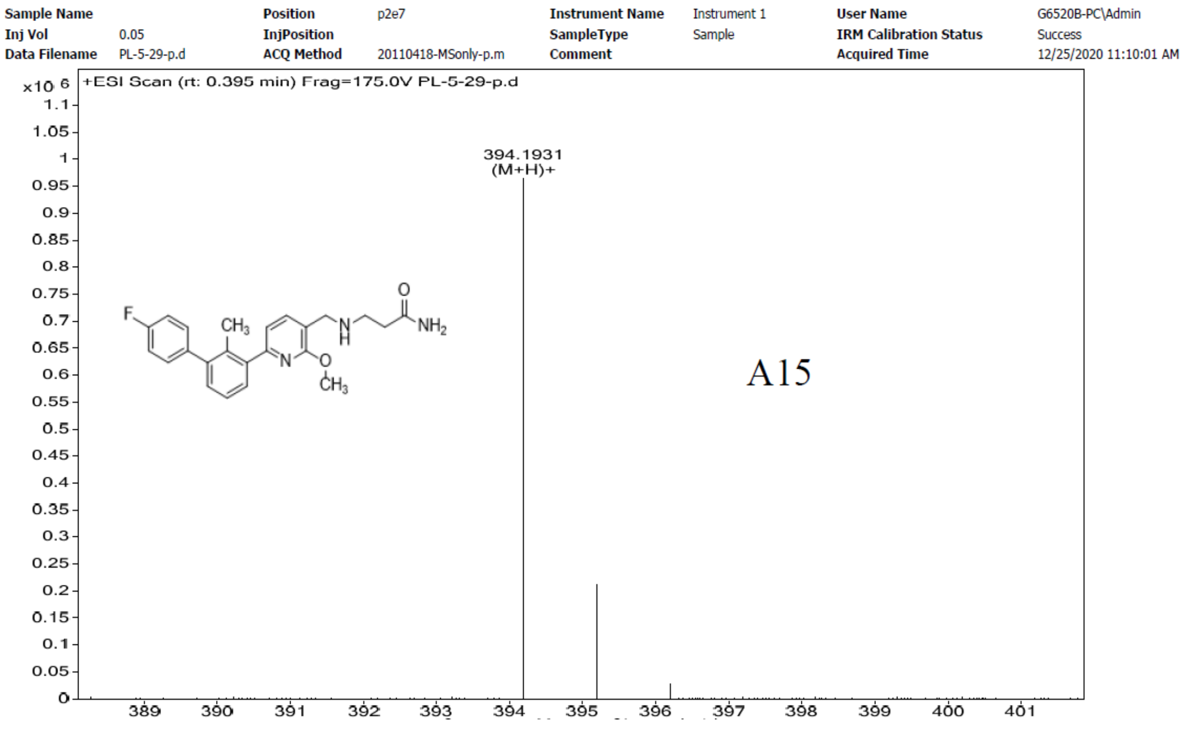

^{13}C NMR Spectra for Compound **A15**

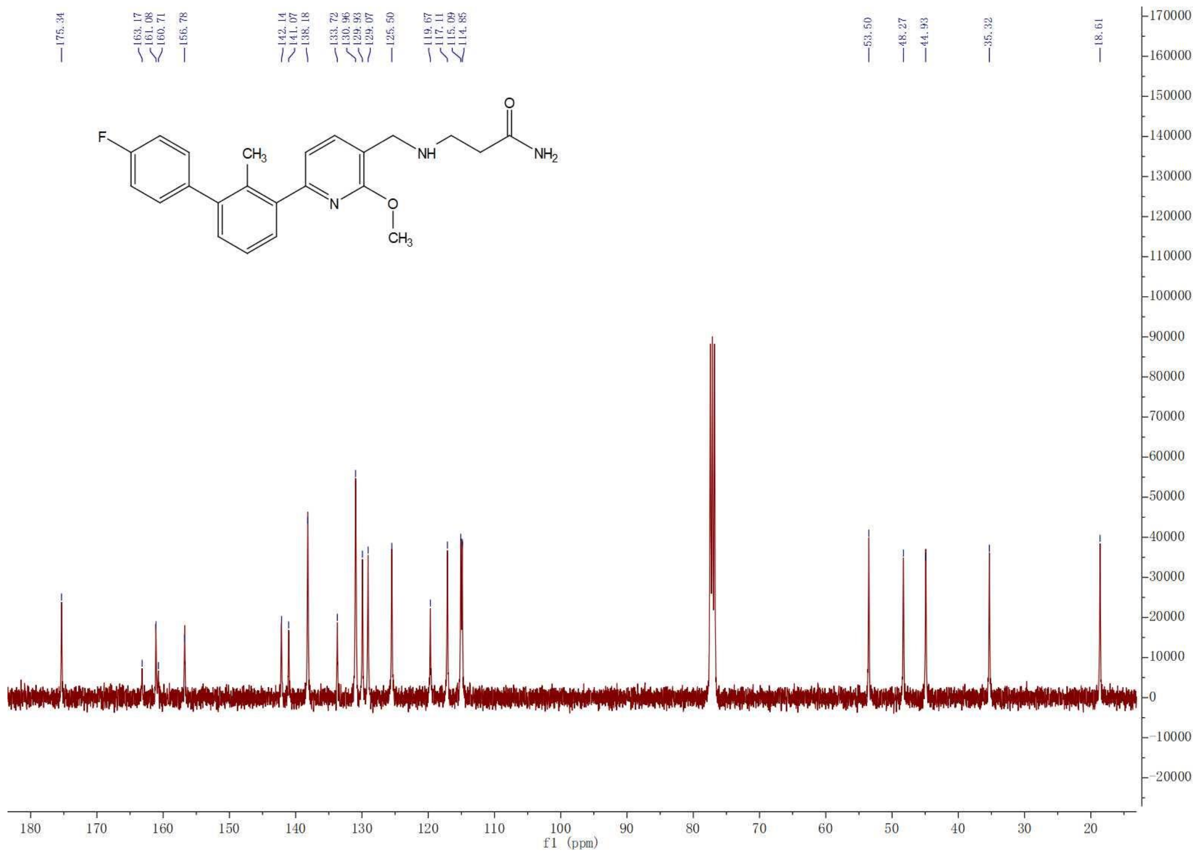

^{1}H NMR Spectra for Compound **A16**

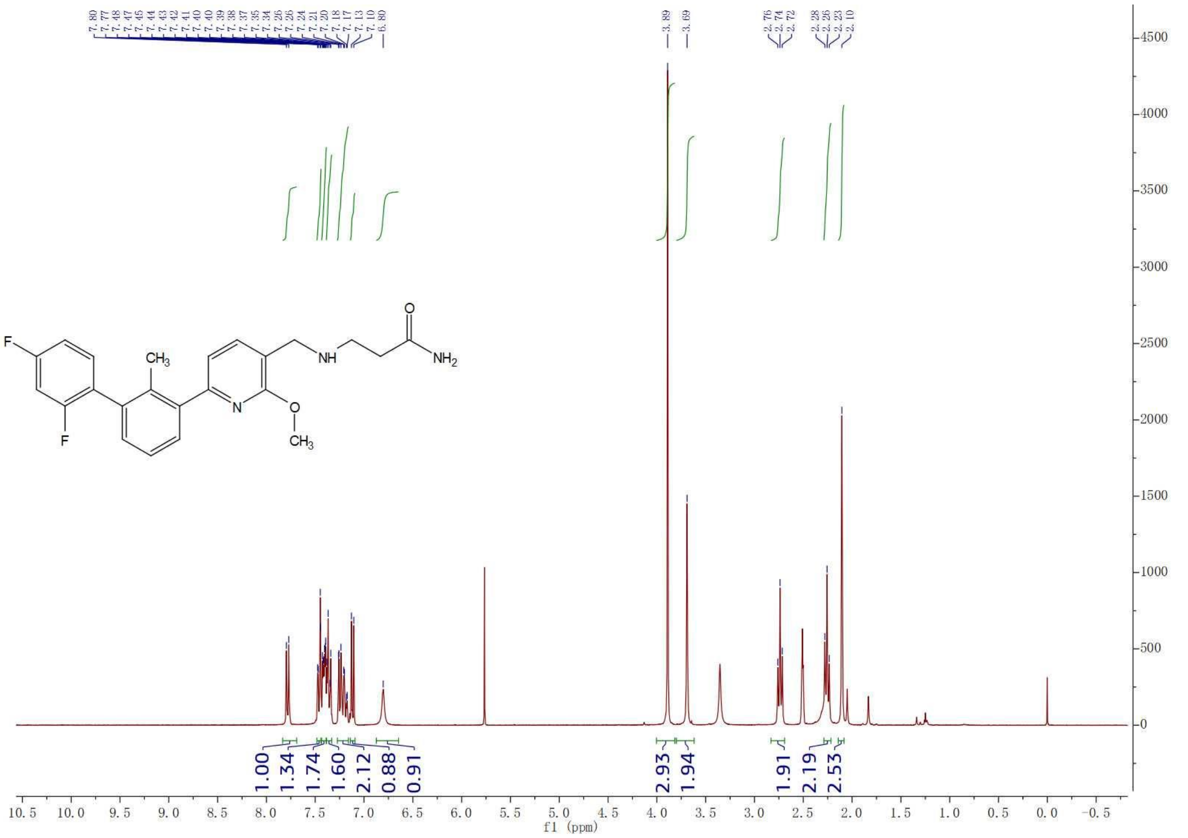

LCMS Spectra for Compound **A16**

**Sample Name**		**Position**	p2e8	**Instrument Name**	Instrument 1	**User Name**	G6520B-PC\Admin
**Inj Vol**	0.05	**InjPosition**		**SampleType**	Sample	**IRM Calibration Status**	Success
**Data Filename**	PL-5-30-p.d	**ACQ Method**	20110418-MSonly-p.m	**Comment**		**Acquired Time**	12/25/2020 11:12:06 AM

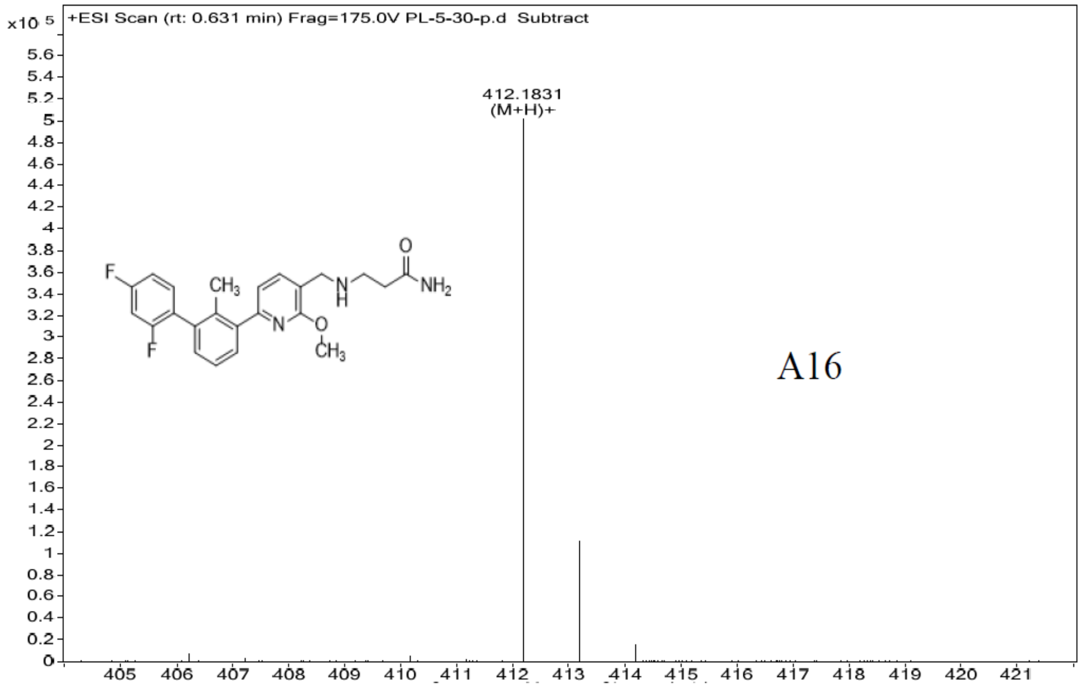

^{13}C NMR Spectra for Compound **A16**

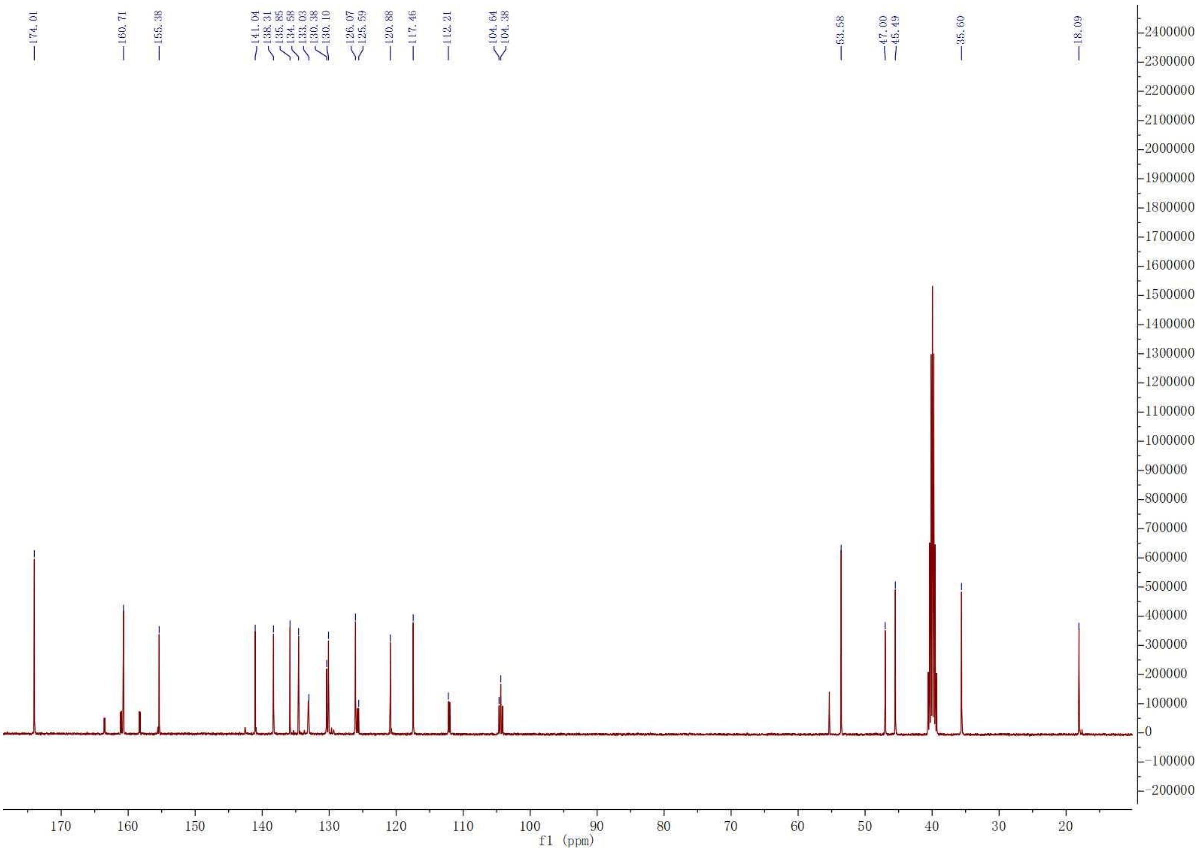

^{1}H NMR Spectra for Compound **A17**

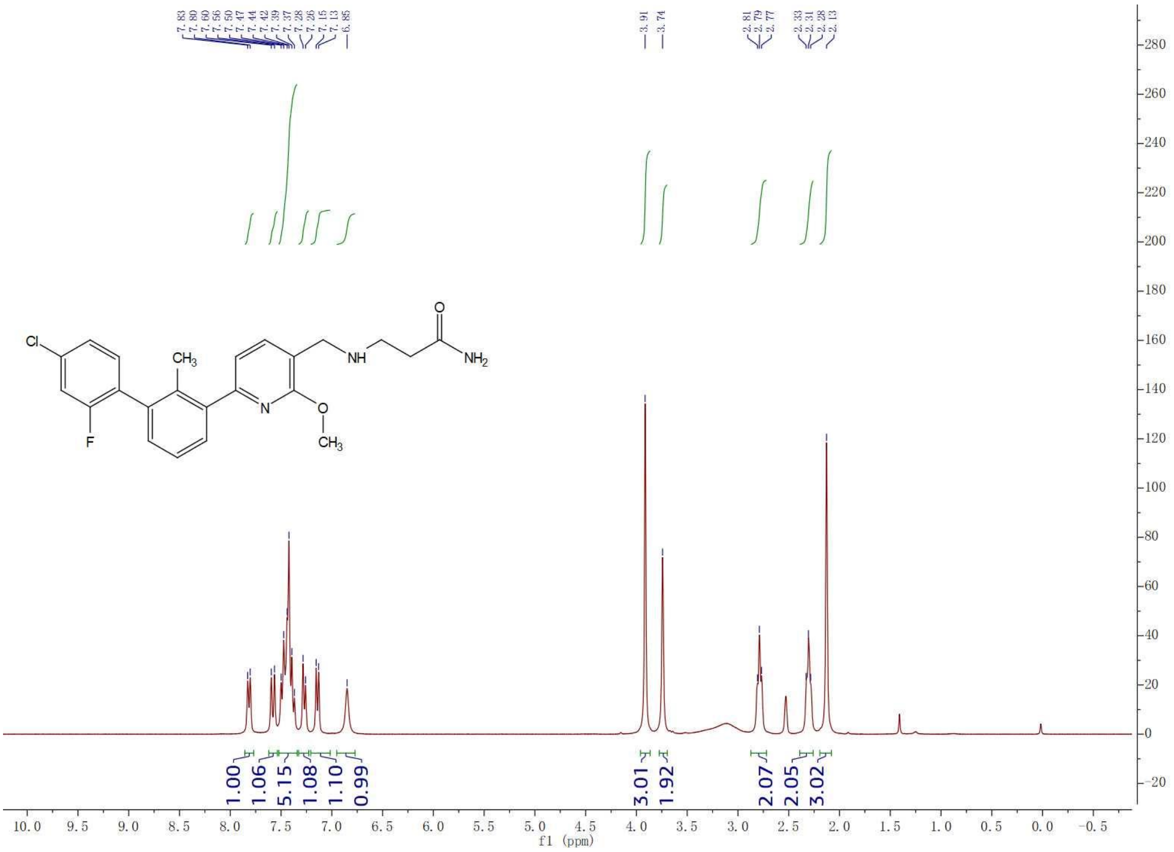

LCMS Spectra for Compound **A17**

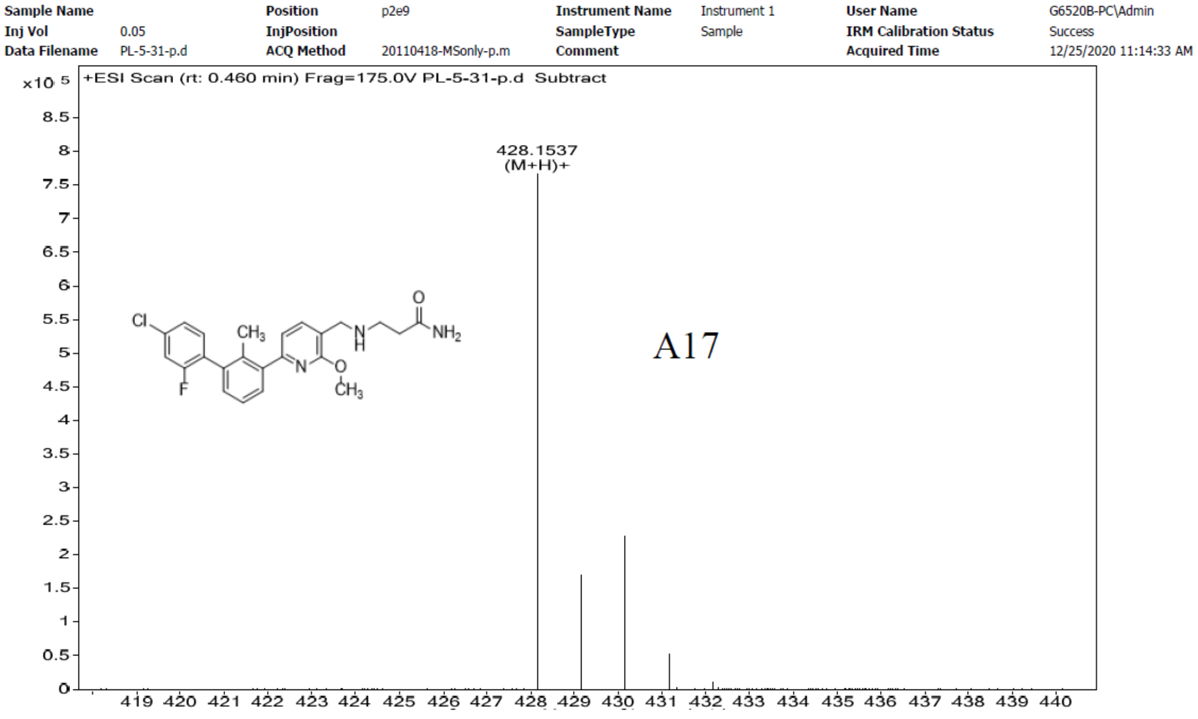

^{13}C NMR Spectra for Compound **A17**

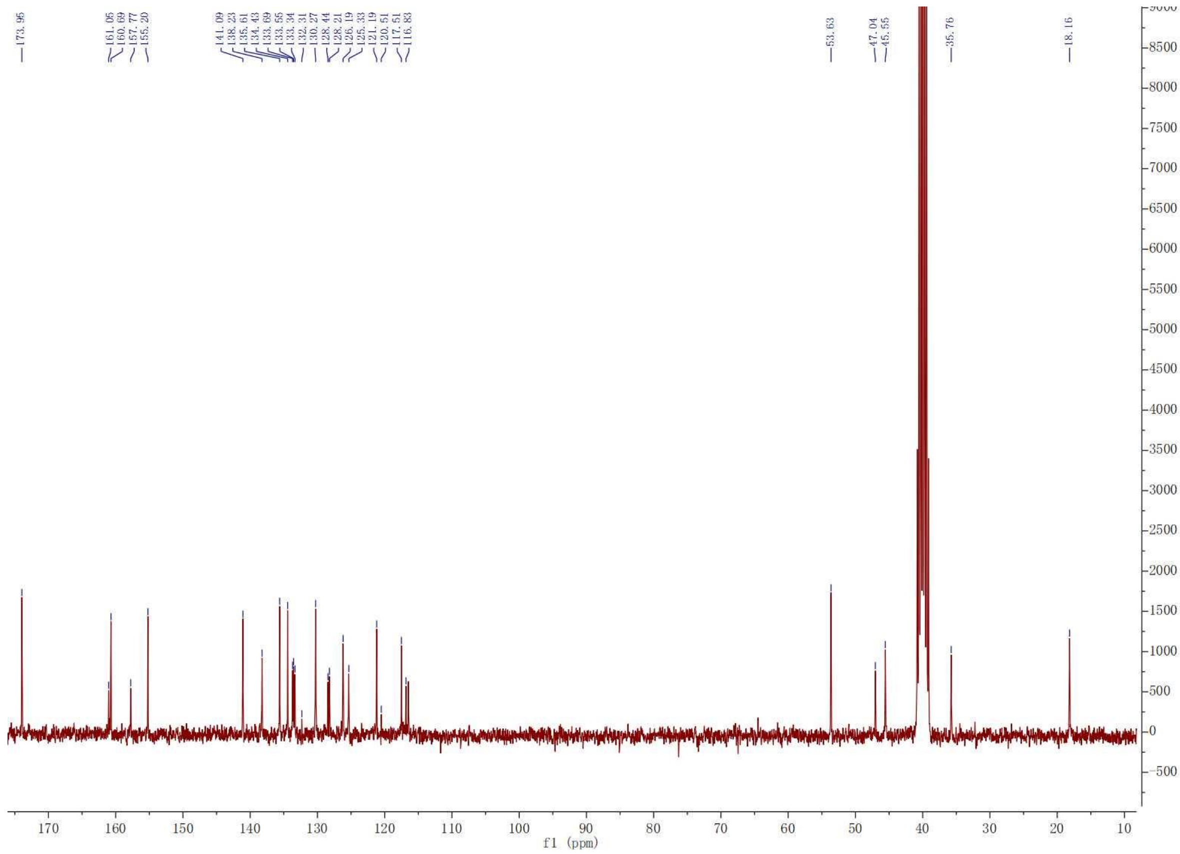

^{1}H NMR Spectra for Compound **A18**

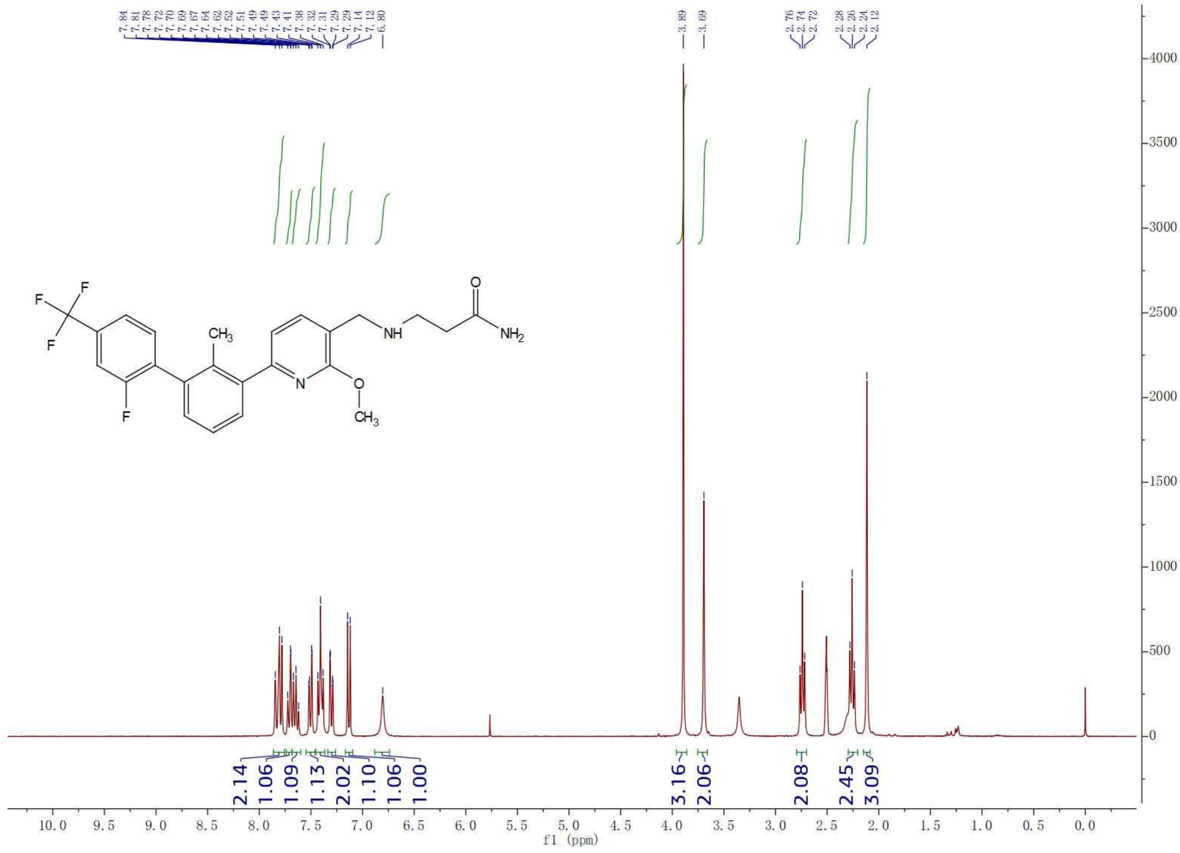

LCMS Spectra for Compound **A18**

**Sample Name**		**Position**	p2f1	**Instrument Name**	Instrument 1	**User Name**	G6520B-PC\Admin
**Inj Vol**	0.05	**InjPosition**		**SampleType**	Sample	**IRM Calibration Status**	Success
**Data Filename**	PL-5-32-p.d	**ACQ Method**	20110418-MSonly-p.m	**Comment**		**Acquired Time**	12/25/2020 11:16:54 AM

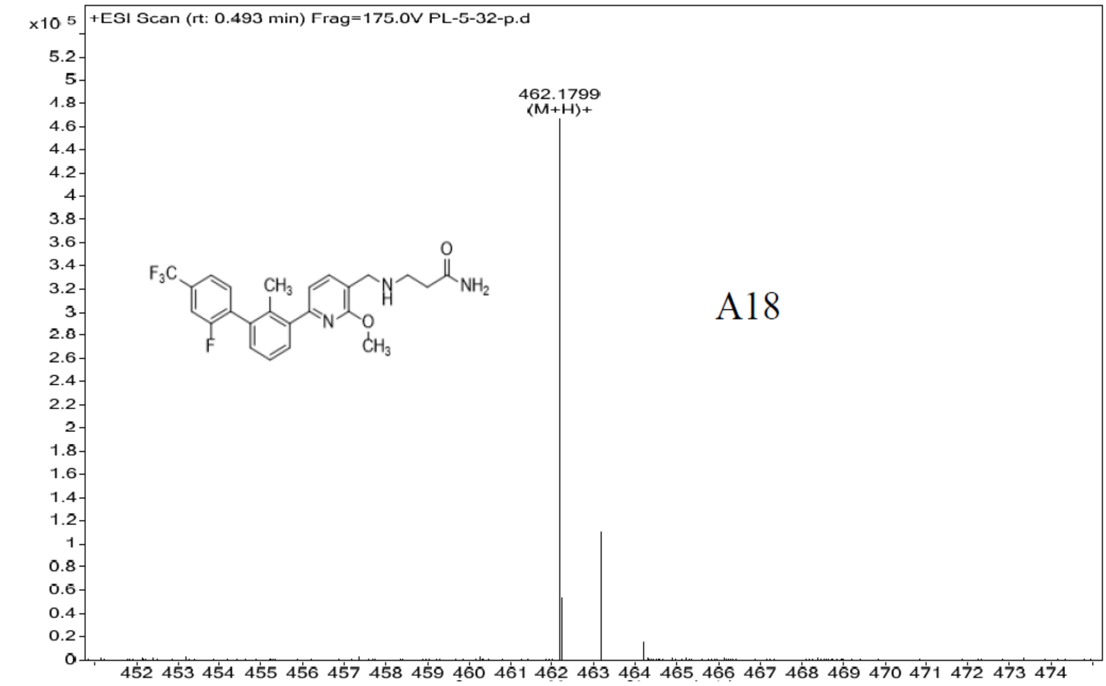

^{13}C NMR Spectra for Compound **A18**

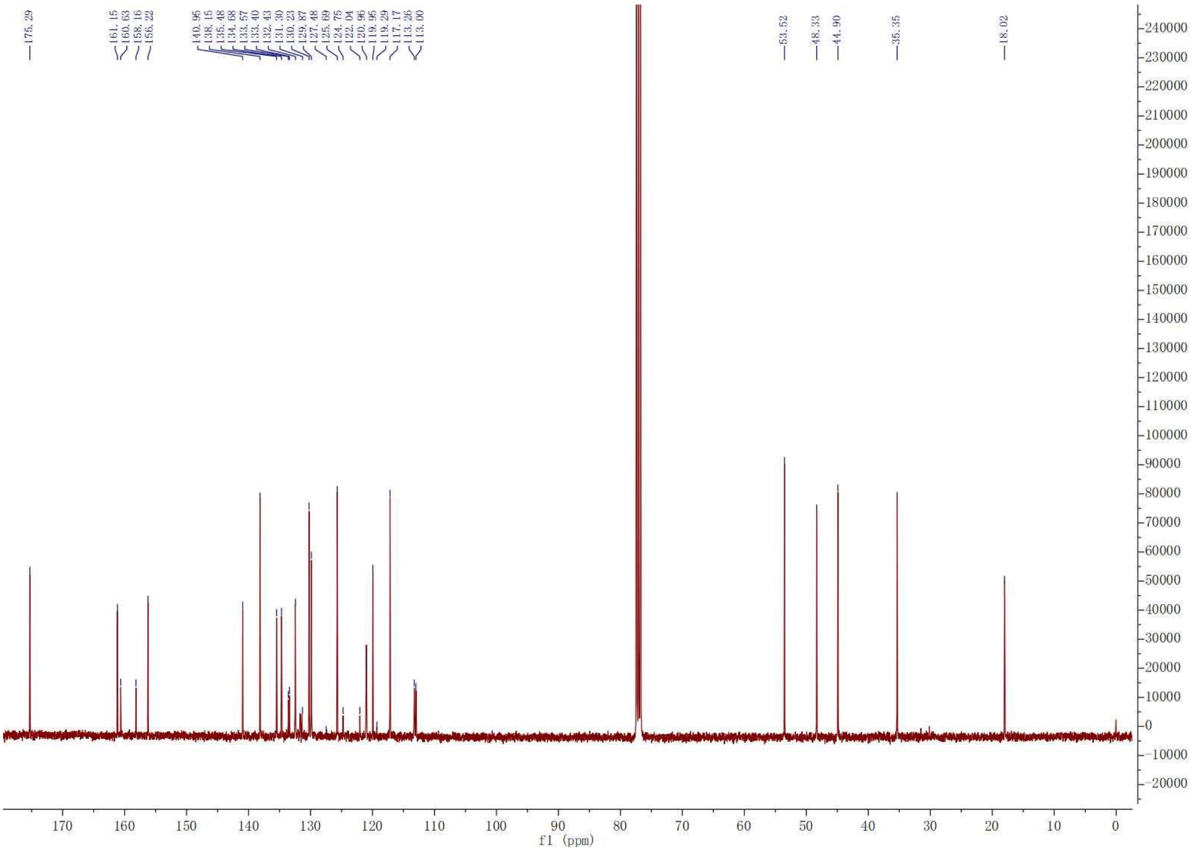

III. HPLC Spectroscopic data for A9

SHIMADZU LabSolutions 分析报告

<样品信息>

样品名	: A9		
样品ID	: A9		
数据文件名	: A9.lcd		
方法文件名	: 通用方法.lcm		
批处理文件名	: 123156.lcb		
样品瓶号	: 1-16	样品类型	: 未知
进样体积	: 10 uL		
分析日期	: 2021/1/14 15:34:51	分析者	: System Administrator
处理日期	: 2021/1/14 16:01:59	处理者	: System Administrator

<色谱图>

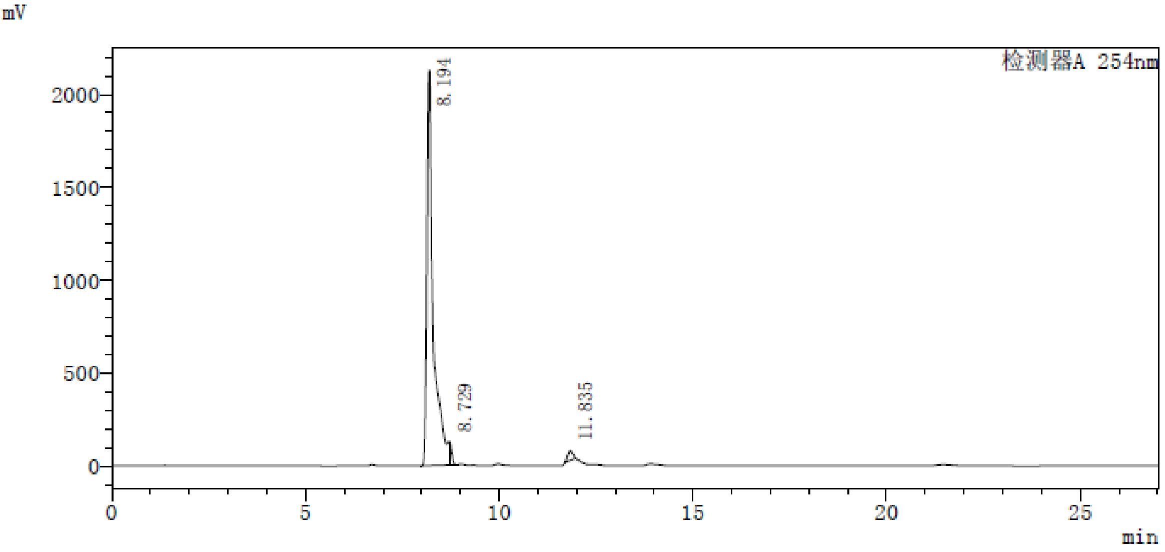

<峰表>

检测器A 254nm

峰号	保留时间	面积	高度	浓度	浓度单位	标记	化合物名
1	8.194	24072269	2131681	96.638		M	
2	8.729	382207	121915	1.534		V M	
3	11.835	455384	49034	1.828		M	
总计		24909860	2302630				
